# Over supplementation with vitamin B12 alters microbe-host interactions in the gut leading to accelerated *Citrobacter rodentium *colonization and pathogenesis in mice

**DOI:** 10.1186/s40168-023-01461-w

**Published:** 2023-02-03

**Authors:** Andrew J. Forgie, Deanna M. Pepin, Tingting Ju, Stephanie Tollenaar, Consolato M. Sergi, Samantha Gruenheid, Benjamin P. Willing

**Affiliations:** 1 Department of Agricultural, Food and Nutritional Science, University of Alberta, Edmonton, Alberta T6G 2P5 Canada; 2 Division of Anatomic Pathology, Children’s Hospital of Eastern Ontario (CHEO), Ottawa, Ontario Canada; 3 Faculty of Medicine and Health Sciences, McGill University, Montreal, Quebec, Canada

**Keywords:** Cobalamin, Infection, Symbiosis, Microbiota, Health, Gastrointestinal

## Abstract

**Background:**

Vitamin B12 supplements typically contain doses that far exceed the recommended daily amount, and high exposures are generally considered safe. Competitive and syntrophic interactions for B12 exist between microbes in the gut. Yet, to what extent excessive levels contribute to the activities of the gut microbiota remains unclear. The objective of this study was to evaluate the effect of B12 on microbial ecology using a B12 supplemented mouse model with *Citrobacter rodentium*, a mouse-specific pathogen. Mice were fed a standard chow diet and received either water or water supplemented with B12 (cyanocobalamin: ~120 μg/day), which equates to approximately 25 mg in humans. Infection severity was determined by body weight, pathogen load, and histopathologic scoring. Host biomarkers of inflammation were assessed in the colon before and after the pathogen challenge.

**Results:**

Cyanocobalamin supplementation enhanced pathogen colonization at day 1 (*P* < 0.05) and day 3 (*P* < 0.01) postinfection. The impact of B12 on gut microbial communities, although minor, was distinct and attributed to the changes in the *Lachnospiraceae* populations and reduced alpha diversity. Cyanocobalamin treatment disrupted the activity of the low-abundance community members of the gut microbiota. It enhanced the amount of interleukin-12 p40 subunit protein (IL12/23p40; *P* < 0.001) and interleukin-17a (IL-17A; *P* < 0.05) in the colon of naïve mice. This immune phenotype was microbe dependent, and the response varied based on the baseline microbiota. The cecal metatranscriptome revealed that excessive cyanocobalamin decreased the expression of glucose utilizing genes by *C. rodentium*, a metabolic attribute previously associated with pathogen virulence.

**Conclusions:**

Oral vitamin B12 supplementation promoted *C. rodentium* colonization in mice by altering the activities of the *Lachnospiraceae* populations in the gut. A lower abundance of select *Lachnospiraceae* species correlated to higher p40 subunit levels, while the detection of *Parasutterella* exacerbated inflammatory markers in the colon of naïve mice. The B12-induced change in gut ecology enhanced the ability of *C. rodentium* colonization by impacting key microbe-host interactions that help with pathogen exclusion. This research provides insight into how B12 impacts the gut microbiota and highlights potential consequences of disrupting microbial B12 competition/sharing through over-supplementation.

**Graphical Abstract:**

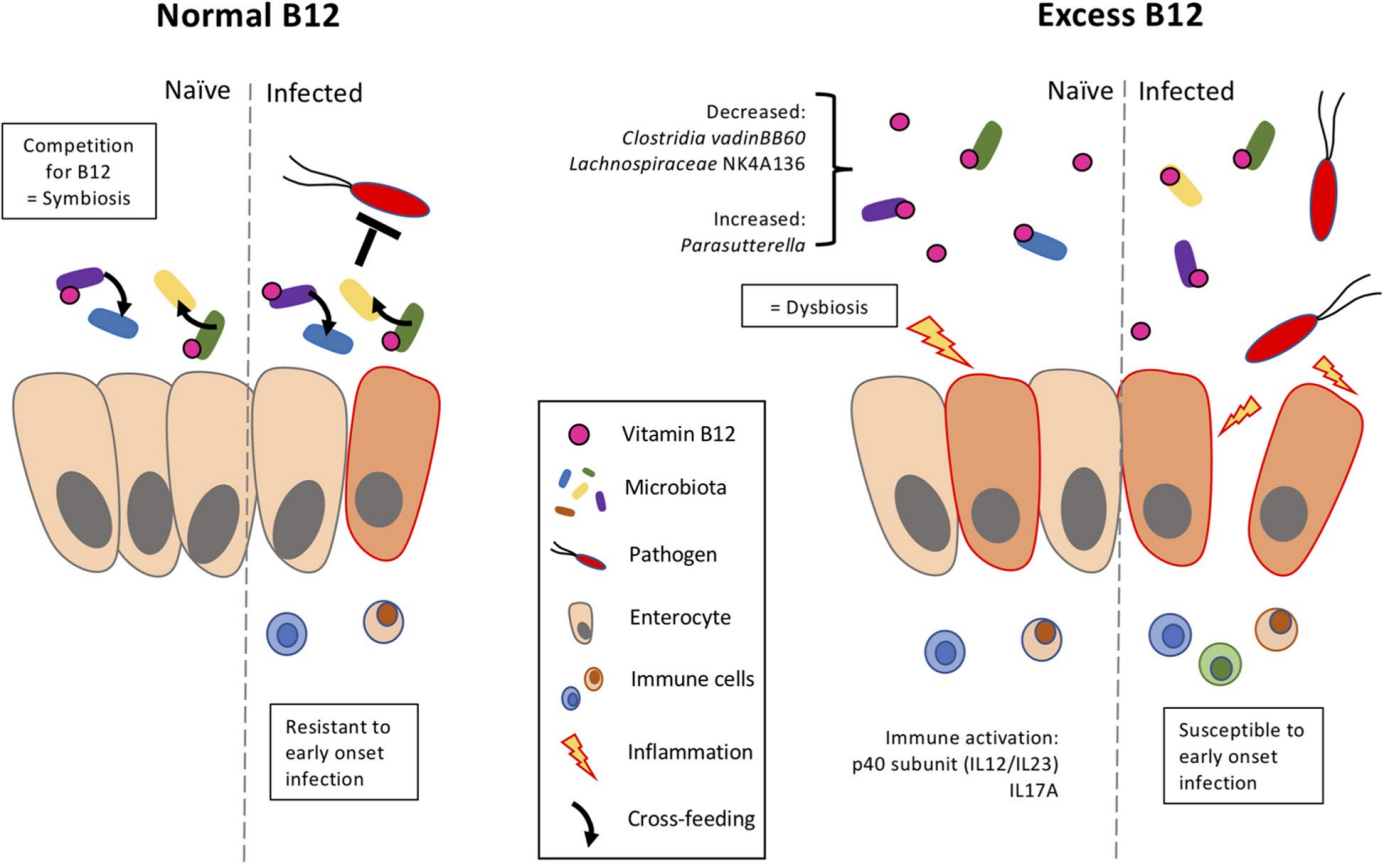

Video Abstract

**Supplementary Information:**

The online version contains supplementary material available at 10.1186/s40168-023-01461-w.

## Background

Vitamin B12 (cobalamin) is a cobalt-containing corrinoid molecule required for fundamental biological processes in both humans and bacteria. It is made exclusively by microorganisms and belongs to a family of organometallic cofactors called cobamides [1]. With few exceptions, such as ruminants which depend on the biosynthesis of cobalamin by resident microbes, most animals rely on its bioaccumulation in the food chain [2]. Humans must obtain cobalamin through diet because the only significant population of microbes that could produce cobalamin resides in the colon, past the absorption site in the small intestine [3]. Dietary sources of cobalamin in humans are mainly of animal origin, but supplements, fortified food products, fermented foods, and some plants and algae are available as alternatives [4]. Oral supplementation is an effective and more attractive option to replenish B12 stores than intramuscular injections [5, 6]. It is important to note that excess B12 is excreted mainly through bile and can be reabsorbed through enterohepatic circulation; however, any unabsorbed B12 will reach microbial communities in the gut [7]. To what extent high levels of B12 influence the gastrointestinal (GI) microbiota and host health remains poorly understood.

Cobamides are essential for bacteria, playing a significant role in supporting enzyme activity in the cytosol and as coenzyme riboswitches that regulate gene expression in the nucleus [8]. Genomic studies revealed that widespread cobalamin sharing between microbes can impact microbial growth and metabolism through numerous cobamide-/corrinoid-dependent enzymes and transporter proteins [9–11]. Because of this, B12 and cobamide derivatives likely play a more critical role in microbe-microbe interactions that modulate microbial ecosystems than previously understood [3]. For example, B12 uptake by the gut commensal *Bacteroides thetaiotaomicron* was shown in vitro to limit the production of Shiga toxin-2 produced by enterohemorrhagic *Escherichia coli* O157:H7 (EHEC) [12]. This has been attributed to reducing the ability of EHEC to use ethanolamine (a breakdown product of lipid membranes and major metabolite found in the gut) by limiting the availability of cobalamin required to activate adenosylcobalamin-dependent ethanolamine ammonia-lyase. Regulation of ethanolamine metabolism influences the growth and/or virulence of several *Enterobacteriaceae* and *Firmicutes* species [13]. Therefore, it follows that competition among microbes for cobalamin can impact the activity of ethanolamine-utilizing bacteria. In addition, *B. thetaiotaomicron* creates competition by using a surface-exposed lipoprotein, which binds cobalamin with such affinity that can remove it from intrinsic factor, a cobalamin transport protein necessary for absorption in humans and animals [14]. However, additional competitive and syntrophic interactions may exist between microbes for cobalamin in the GI tract and to what extent this impacts bacterial pathogenesis in the gut remains poorly understood.

Daily oral cobalamin supplements can contain doses that far exceed (as high as 10,000 μg/tablet) the recommended daily amount of 2.4 μg/day in humans. High exposures are required when normal absorption is impeded and are generally considered safe. The practice of over-supplementing cobalamin to ensure adequate absorption is supported by the fact that no upper limit has been set for B12 supplementation. A study in C57BL/6J mice using a dose comparable to a 5000 μg tablet (30.46 μg/day) for humans found a depletion in *Bacteroides* but no change in cecal short-chain fatty acids (SCFAs) or markers of dextran sulfate sodium (DSS)-induced colitis with B12 treatment, including colon length and fluorescein isothiocyanate-dextran permeability test [15]. Since competition exists between enteric pathogens and commensal microbes for B12 sequestering and sharing, we hypothesized that excessive cobalamin supplementation alters the gut microbiota’s functional activity, creating a favorable environment for pathogen colonization and pathogenesis. Due to the fact that no upper limit has been determined for B12, we supplemented a megadose of B12 to mice in drinking water and challenged with *Citrobacter rodentium*, a natural mouse-specific pathogen that mirrors the attaching and effacing pathology seen in human EHEC infections [16]. We evaluated the direct and indirect impact of oral B12 supplementation on the gut microbiota and ability of the host to resist enteric pathogen colonization and pathogenesis.

## Methods

### Mice and vitamin B12 supplementation

Animal experiments were conducted in accordance with guidelines set by the Canadian Council on Animal Care and approved by the Animal Care and Use Committee at the University of Alberta (Edmonton, AB, Canada). All mice were raised and maintained under specific pathogen-free (SPF) or germ-free (GF) conditions. Six- to 7-week-old female C3H/HeOuJ mice (Jackson Laboratories, Maine, USA) were randomly housed four or five per cage. An 8-week-old female C57BL/6J mice (University of Alberta, AB, Canada) were housed three per cage using the Tecniplast Isocage-P bioexclusion system (Buguggiate, VA, Italy). An 8-week-old germ-free female C57BL/6J mice were housed in an isolator (CEP Standard Safety, McHenry, IL, USA) with open cages in the University of Alberta Axenic Mouse Research Unit. All mice were allowed to acclimatize for 1 week with ad libitum access to water and standard chow containing approximately 0.08 mg of cyanocobalamin per kilogram of diet post-autoclaving (2020SX; Envigo-teklad, Indiana, USA). Calculated based on the average consumption of 5 g of diet per mouse per day, the standard chow diet alone contributed approximately 0.4 μg of cyanocobalamin. Mice received filter-sterilized drinking water supplemented with or without B12 in the form of cyanocobalamin (V2876, Sigma-Aldrich, St. Louis, MO, USA) at 40 μg/ml, approximately 100 times the amount in the 2020SX diet for 2 weeks in the following experiments:

1. After 2 weeks of water treatment, a survival (SURV) experiment was performed in C3H/HeOuJ mice (*n* = 12) using the *C. rodentium*-challenge model described below. A 20% loss of initial body weight was selected as a humane endpoint for mice in the SURV experiment as previously described [17].
2. Early-stage pathogen colonization (EPC) experiment was performed in C3H/HeOuJ mice (*n* = 16) to investigate the onset of pathogen colonization during the early phases of infection. Two mice per cage from each group were euthanized (naïve_CON & naïve_CNCbl40), and the remaining mice were challenged with *C. rodentium* while continuing water treatment (inf_CON & inf_CNCbl40) for a total of 8 mice per group.
3. Different forms of B12 were investigated in conventional C57BL/6J mice housed three per cage (*n* = 6) by supplementing cyanocobalamin and methylcobalamin (Thermo Fisher Scientific, MA, USA) at 10 μg/ml and 40 μg/ml in drinking water.
4. Germ-free C57BL/6J mice (*n* = 4) were used to test the direct role of cyanocobalamin at the 40 μg/ml dose to corroborate the change in colonic inflammation markers found in the conventional mice.

In all experiments, cages were randomly assigned to treatment groups: control (CON) and B12 supplemented (cyanocobalamin at 10 μg/ml, CNCbl10, and 40 μg/ml, CNCbl40; methylcobalamin at 10 μg/ml, MeCbl10, and 40 μg/ml, MeCbl40) groups accordingly.

### Water intake and B12 dose estimation

A pilot study (data not shown) was conducted to determine daily water consumption of mice supplemented with B12 (cyanocobalamin) in drinking water. Cyanocobalamin at 40 μg/ml or control drinking water was provided to mice (*n* = 15; five mice per cage) ad libitum, and water consumption was monitored for a week. Drinking water was changed every 2 days, and water consumption was measured. The water consumed per cage at each timepoint was considered a replicate, and the average was used to compare water intake between treatments. B12 supplemented in drinking water did not impact water consumption. Daily water intake was approximately 3 ml per mouse per day.

According to water intake, B12 supplemented in drinking water at 10 μg/ml and 40 μg/ml were estimated to reach a total dose of 30 μg and 120 μg per mouse a day, respectively. The human equivalent dose for the cyano- and methyl-cobalamin drinking water used in these studies is ~5000 μg/day (CNCbl10 & MeCbl10) and ~25,000 μg/day (CNCbl40 & MeCbl40), which was calculated using the standard human equivalent dose parameters [18]. A megadose was used in the present study to push the phenotype and ensure high B12 levels were maintained in the gut to disrupt competition, cross-feeding and microbe-host interactions related to B12 utilization by the gut microbiota. These levels were considered feasible within the human population, where 10,000 μg B12 capsules are available and would be taken as a single bolus. Comparing B12 supplementation between humans and mice using a multiplicity factor of their respective recommended dietary allowance (RDA) shows that a dose of 5000–10,000 μg tablet per day in an adult human is equal to 2083–4167 × their RDA of 2.4 μg/day, whereas mice receiving a 120 μg/day in the present study is equal to 2400 × their RDA. The RDA of B12 for mice is approximately 0.05 μg/day and is based on a diet containing 10 μg of B12 per kilogram that was deemed adequate for mice [19].

### *C. rodentium*-challenge model

From a glycerol stock, *C. rodentium* (DBS100) was plated on MacConkey agar (BD Difco, NJ, USA), and a single colony was picked and incubated overnight at 37 °C in Luria-Bertani broth (Sigma-Aldrich) with shaking at 200 rpm. Mice were infected with 100 μl of the overnight culture (1 × 10^9^ CFU/ml) by oral gavage. All mice were confirmed to be free of coliforms by plating a fecal sample on MacConkey agar prior to infection. Pathogen load was determined daily in fecal samples and in the GI content at day 5 postinfection by plating serial dilutions of sample homogenates in 1 × PBS on MacConkey agar. Plates were incubated at 37 °C overnight, and colonies were counted and normalized to sample weight.

### Sample collection

Fresh fecal samples were collected daily or every 2nd day postinfection directly in 1 ml of sterile 1 × PBS for plating. All mice were euthanized using carbon dioxide, and sampling was done aseptically. Prior to infection, fecal samples were collected from mice for baseline microbiota analysis. Mouse tissues and intestinal content (ileum, cecum, and colon) were snap-frozen in liquid nitrogen and stored at −80 °C until use.

### Intestinal vitamin B12 level measurement

Snap-frozen cecal and colonic digesta samples were weighed and homogenized with two rounds of beating (30 s at 4 m/s with a cooling step on ice) in a proprietary buffer provided by Calgary Laboratory Services: Diagnostic and Scientific Research Centre (Calgary, AB, Canada). Samples were subsequently centrifuged at 10,000 rpm, and the supernatant was collected and stored at −20 °C. Vitamin B12 was quantified via electrochemiluminescence using the Roche Diagnostics vitamin B12 II assay performed on the Roche Diagnostics e602 (Calgary Laboratory Services). This technique measures total vitamin B12 using a ruthenium-label recombinant porcine intrinsic factor as the reporter probe.

### Short-chain fatty acids (SCFA) analysis

Snap-frozen cecal content was thawed on ice and weighed (30 mg/sample) and homogenized in 600 μl of 25% phosphoric acid. Samples were centrifuged at 15,000 rpm at 4 °C for 10 min, and the supernatant was passed through a 0.45 μm syringe filter (Fisher). A 200 μl aliquot of filtered sample was combined with 50 μl of internal standard (23 μmol/ml, isocaproic acid) and analyzed on a SCION 456-GC instrument.

### Cecal microbial metatranscriptome analysis

Total RNA was extracted from frozen cecal samples as previously described [20]. Approximately 50 mg of frozen cecal content was added to 0.1 mm glass bead-containing tubes (PowerBead Tubes, Qiagen) prefilled with 300 μl RLT buffer (RNeasy mini kit, Qiagen) supplemented with β-mercaptoethanol (10 μl/ml, Sigma-Aldrich) and 1 ml TRIzol (Invitrogen). Cell disruption was accomplished using a FastPrep®-24 bead-beating machine (MP biomedicals) with two rounds of beating (30 s at 6.5 m/s). After incubating for 5 min at room temperature, samples were centrifuged (1 min, 12,000 × g, 4 °C), and supernatants were transferred into tubes containing 300 μl of chloroform and vortexed and incubated for 3 min. After centrifugation (15 min, 12,000 × g, 4 °C), the upper aqueous phase was carefully collected and transferred into a new tube containing 1 ml of freshly prepared 70% ethanol solution, mixed by pipetting, and loaded onto a RNeasy spin column (RNeasy mini kit, Qiagen). RNA extraction and on-column DNA digestion (Qiagen) were completed as described by the manufacturer’s protocol. The quality and quantity of RNA were measured using an Agilent Bioanalyzer. Samples with an RNA integrity number (RIN) ≥ 7.0 were used to generate metatranscriptome libraries at Génome Québec Innovation Centre (Montréal, QC). Samples were diluted to 100 ng/μl, and host rRNA-depletion (NEBNext® Human/Mouse/Rat) was conducted. The libraries were sequenced as 100 bp paired-end reads on a NovaSeq 6000 system (Illumina).

Analysis of unfiltered raw data (~35M read average per sample) was completed using the Simple Annotation of Metatranscriptomes by Sequence Analysis 2.0 (SAMSA2) pipeline [21] as follows: PEAR (version 0.9.10) to merge reads, Trimmomatic (version 0.36) to trim low-quality reads, ShortMeRNA (version 2.1) to remove rRNA, and DIAMOND (version 2.0.2) to annotate mRNA data to the RefSeq database [22]. The merging step resulted in ~25 M merged reads per sample. Bacterial rRNA made up ~9 M reads per sample, and ribodepleted reads (mRNA transcripts) led to ~2 M annotated reads with ~13 M unknown reads per sample. In addition, the SEED subsystems hierarchical database [23] was used to categorize and compare functional activities of the microbiome. Analysis of annotated reads was completed using the DESeq2 package and visualized in R with the ggplot2 package.

### Cytokine and chemokine assays

Protein was extracted using a 2-cm piece of distal colon and homogenized in 300 μl of Meso Scale Discovery lysis buffer with protease and phosphatase inhibitors as described in the assay protocol. The homogenates were centrifuged at 15,000 rpm for 10 min, and the protein concentration in the supernatant was determined using the Pierce Bicinchoninic Acid assay Kit (Thermo Scientific). Sample homogenates from naïve mice and infected mice were loaded into wells at 150 μg and 100 μg of total protein, respectively. The U-PLEX Biomarker Group 1 (mouse) assay platform (Meso Scale Discovery, Gaithersburg, MD, USA) was used to measure interferon gamma (INFγ), interleukins (IL-1β, IL-4, IL-6, IL-10, IL-12/23p40, IL-17A, and IL-22), keratinocyte-derived chemokine (KC), tumor necrosis factor alpha (TNFα), granulocyte-macrophage colony-stimulating factor (GM-CSF), matrix metalloproteinase-9 (MMP9), chemokine protein known as regulated on activation normal T cell expressed and secreted (RANTES), interferon gamma-induced protein-10 (IP10), monocyte chemoattractant protein-1 (MCP1), and macrophage inflammatory proteins (MIP1α, MIP2, and MIP3α). Final concentrations were presented as pg/ml in 100 μg of total colon protein.

### Microbial community analyses

Total DNA was extracted from ileum, cecum, and colon contents using the QIAamp Fast DNA Stool Mini Kit (Qiagen, Valencia, CA, USA) with an additional bead-beating step using ~200 mg of garnet rock at 6.0 m/s for 60 s on a FastPrep-24 5G instrument (MP Biomedicals). Amplicon libraries were constructed according to the protocol from Illumina (16S Metagenomic Sequencing Library Preparation) that amplified the V3-V4 region of the bacterial 16S rRNA gene: 341F (5′-TCGTCGGCAGCGTCAGATGTGTATAAGAGACAGCCTACGGGNGGCWGCAG- 3′) and 805R (5′-GTCTCGTGGGCTCGGAGATGTGTATAAGAGACAGGACTACHVGGGTATCTAATCC-3′). Paired-end sequencing was accomplished using an Illumina MiSeq Platform (2 × 300 cycles; Illumina Inc., San Diego, CA, USA). Raw sequences were processed with Quantitative Insight into Microbial Ecology 2 (QIIME) [24] pipeline using the divisive amplicon denoising algorithm 2 (DADA2) to filter, trim, and merge paired-end reads into amplicon sequence variants (ASVs). Ribosomal RNA data from cecal metatranscriptomic sequencing was processed as pre-merged single-end reads in QIIME2 using deblur denoise-16S function and trimmed at 160 bp. Phylogenetic trees were constructed using the qiime alignment (mafft; mask) and qiime phylogeny (fasttree; midpoint-root) function. Taxonomy was assigned using the qiime feature-classifier classify-sklearn function using the SILVA v138 database trained for the specific amplicon region [25]. QIIME2 files (.qza) were imported into R using qiime2R (version 0.99.4) package and analyzed with phyloseq (version 1.34.0) package [26].

Alpha diversity (Observed, Shannon, phylogenetic diversity (PD)) and beta diversity (weighted and unweighted UniFrac) indices were analyzed with rarefied samples (ileum at 21,497, cecum at 34,012, and colon at 7435 reads) in C3H/HeOuJ mice. In addition, we analyzed the cecal microbial community by analyzing the rRNA (rarefied at 1,247,061 reads) from the metatranscriptome sequencing data. Statistical significance for alpha diversity indices was determine with ANOVA and Tukey correction. Principal coordinate analyses (PCoA) was plotted using the phyloseq package, and clustering significance was determined using the “betadisper” function [27] for dispersion and “pairwiseAdonis.dm” function [28] for orientation. Differential abundance analysis was done with DESeq2 using non-rarefied reads and “tree_glom” (or tax_glom for cecum rRNA) function. The “log2foldchange” of only the ASVs with a *P*-value less than 0.05 was plotted with bolded ASVs signifying the significant adjusted *P*-value < 0.10, < 0.05 (*), < 0.01 (**), and < 0.001 (***). Plotted ASVs were assigned according to their lowest classifiable taxonomic rank and are distinguishable by their corresponding ASV number from most to least abundant. Spearman’s correlation of the colonic microbiota (DESeq2 normalized ASV counts) and immune profiles in colon tissues from naïve (SURV and EPC) and infected (EPC) mice experiments were analyzed and visualized using the “psych” and “pheatmap” packages in R. Significant correlations were plotted on the heatmap as “*” for *P*-value < 0.05 and “#” to denote a trend (*P*-value < 0.10).

### In vitro culture experiments

All in vitro culture experiments were done in an anaerobic chamber (5% CO_2_, 5% H_2_, and 90% N_2_), and cultures were incubated at 37 °C without shaking. *B. thetaiotaomicron* was isolated from C3H/HeOuJ mice fecal samples by serially diluting in 1 × PBS with 0.1% L-cysteine and plating on pre-reduced brain heart infusion (BHI; Difco) agar plus 10% calf blood (Cedarlane, ON, Canada) supplemented with 200 μg/ml of gentamicin [29]. Isolates were identified by amplifying and Sanger sequencing the 16S rRNA gene, and sequences were matched using BLAST web-based tool [30]. *C. rodentium* (10 μl of overnight culture) was inoculated alone or in competition with *B. thetaiotaomicron* (100 μl overnight culture) in 10 ml of pre-reduced low-glucose Dulbecco’s Modified Eagle’s medium (Gibco Life Technologies, Grand Island, NY, USA) supplemented with cyanocobalamin at 0 ppm, 0.01 ppm, and 15 ppm, which was subsequently incubated for 6 h. Overnight cultures grown from a single colony of *C. rodentium* grown in Luria-Bertani broth at 1.4 × 10^7^ CFU/ml and *B. thetaiotaomicron* grown in BHI broth (Difco) at 5.5 × 10^7^ CFU/ml were used as inoculums. Counts were determined by plating on MacConkey agar for *C. rodentium* and the BHI with calf blood agar for *B. thetaiotaomicron*. Total RNA was immediately extracted from 1 ml of pelleted cells with 1 ml of TRIzol reagent and purified using the spin columns as described above.

### Reverse-transcription quantitative PCR

Colon tissues were homogenized in 600 μl of lysis buffer via bead beating, and RNA was extracted using the GeneJET RNA Purification Kit (Thermo Scientific). Samples were treated with DNase as manufacturer’s protocols. RNA samples extracted from both colon tissue and in vitro culture experiment were reverse transcribed using the qFlex cDNA Synthesis Kit (Quanta Bioscience). Primers used for quantitative PCR (Additional file 1 Table S1) were previously validated [29, 31, 32]. The qPCR was performed using PerfeCTa SYBR Green SuperMix (Quantabio) conducted on an ABI StepOne Real-Time System following the cycles: 95 °C for 3 min and 40 cycles of 95 °C for 10 s and 60 °C for 30 s. Gene expression was calculated using the delta-delta Ct (^−ΔΔCt^) method that showed the fold change relative to a housekeeping gene.

### Statistical analysis

Significance testing was conducted using GraphPad Prism 6 (GraphPad software, La Jolla, CA, USA). Student’s *t*-test or ANOVA was used for parametric, and Kruskal-Wallis test was used for nonparametric data. Data were presented as mean ± standard deviation. Survival curve analysis was done using Mantel-Cox test with data up to day 10 post infection. Differences between multiple treatments were corrected by conducting either the Bonferroni’s, Tukey’s, or Dunn’s post hoc comparison test.

## Results

### Cyanocobalamin supplementation enhances early-stage colonization of *C. rodentium *and pathogenesis in C3H/HeOuJ mice

**Fig. 1:**
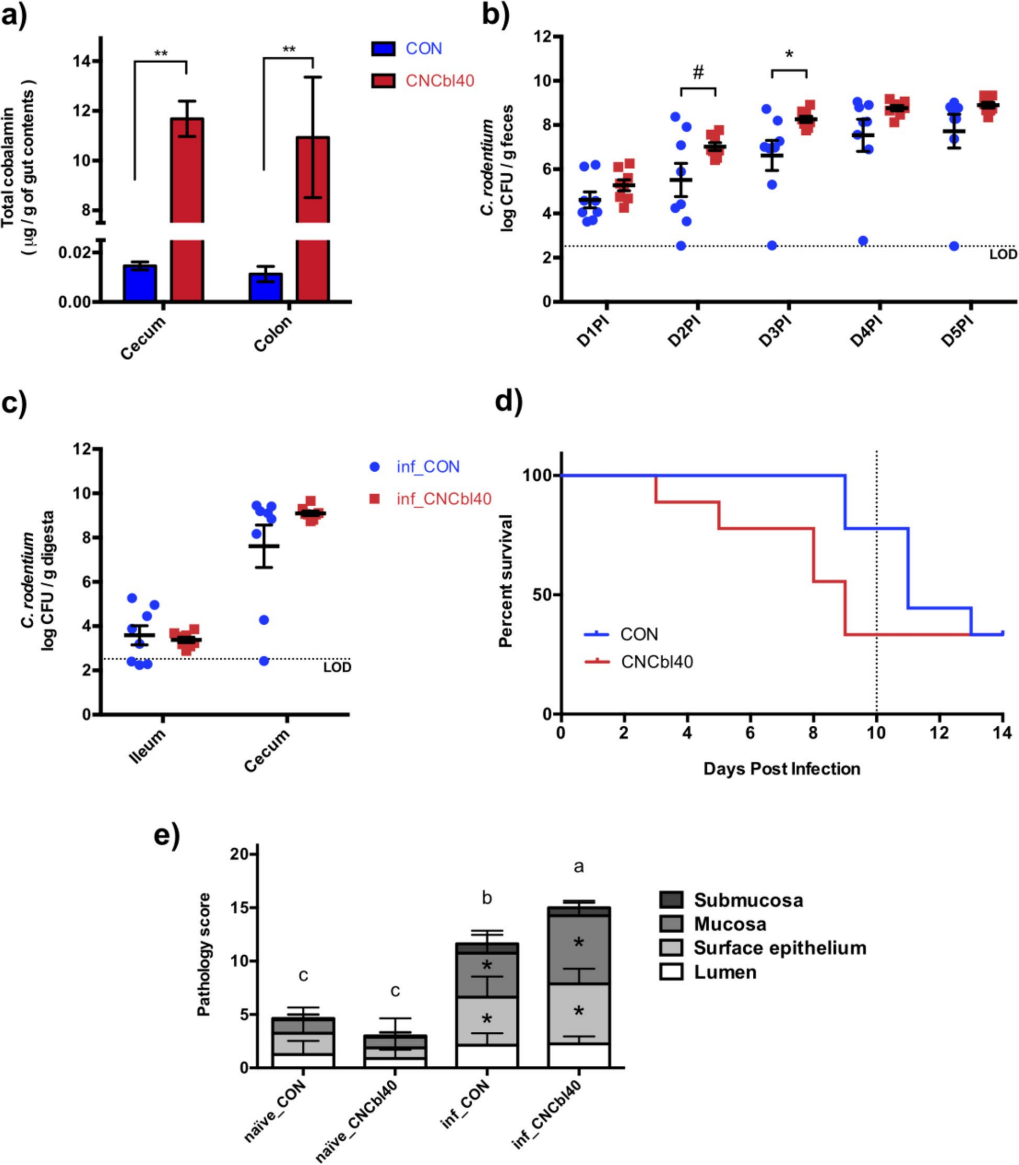
The impact of cyanocobalamin supplementation on survival and early onset of *C. rodentium* infection in C3H/HeOuJ mice. **a** Supplementing cyanocobalamin in drinking water increased cecal and colon levels of cobalamin by ~1000 times (*n* = 15; *P < 0.05, **P < 0.01). **b** Daily fecal enumeration of *C. rodentium* in the EPC experiment indicated increased colonization burden at day 2 postinfection (D2PI) and D3PI from cyanocobalamin supplementation (*n* = 8; #*P* < 0.10, * *P* < 0.05). **c** Enumeration of *C. rodentium* at D5PI in the ileum and cecum was more consistent with cyanocobalamin supplementation, but colonization levels were similar (*n* = 8). **d** Consistent with the more rapid colonization, the SURV experiment revealed an earlier onset of mortality, reducing mice survival over the first 10-day postinfection (*n* = 9; *P* < 0.05; Mantel-Cox test). **e** Colon pathology scores at D5PI in naïve and infected mice show that cyanocobalamin significantly increased mucosal and epithelial damage (*) compared to infected control mice (*n* = 8; *P* < 0.05; limit of detection (LOD))

The amount of total cobalamin in cecum and colon contents of mice were determined to be 1000 times greater (*P* < 0.01) in mice supplemented cyanocobalamin at 40 μg/ml in drinking water compared to control water (Fig. 1 a). Higher cobalamin levels resulted in a more rapid and consistent colonization of *C. rodentium* as determined by daily fecal enumeration (Fig. 1 b). No difference in *C. rodentium* load was detected in the ileum and cecum contents at day 5 postinfection (Fig. 1 c). Differences in pathogen load were greatest at day 3 postinfection (*P* < 0.05). The more rapid and consistent colonization of *C. rodentium* seen in the early pathogen colonization (EPC) experiment was confirmed in the SURV experiment. Mice receiving cyanocobalamin in excess had an earlier onset of mortality that started at day 3 compared to day 9 in control (Fig. 1 d). Survival curves between CON and CNCbl40 were significantly different at day 10 (*P* = 0.03), although significance was lost by day 14 postinfection (*P* = 0.14). Consistent with the increased pathogen load, mice in the inf_CNCbl40 group terminated 5-day postinfection had higher colon pathology scores compared to inf_CON (*P* < 0.05) (Fig. 1 e). Cyanocobalamin water supplementation alone induced no visible tissue damage prior to infection (Fig. 1 e). Overall, colonization and pathogenesis of *C. rodentium* in C3H/HeOuJ mice were enhanced following cyanocobalamin supplementation in drinking water.

### Cobalamin supplementation alters the Firmicutes and Proteobacteria populations within the GI tract of C3H/HeOuJ mice

**Fig. 2:**
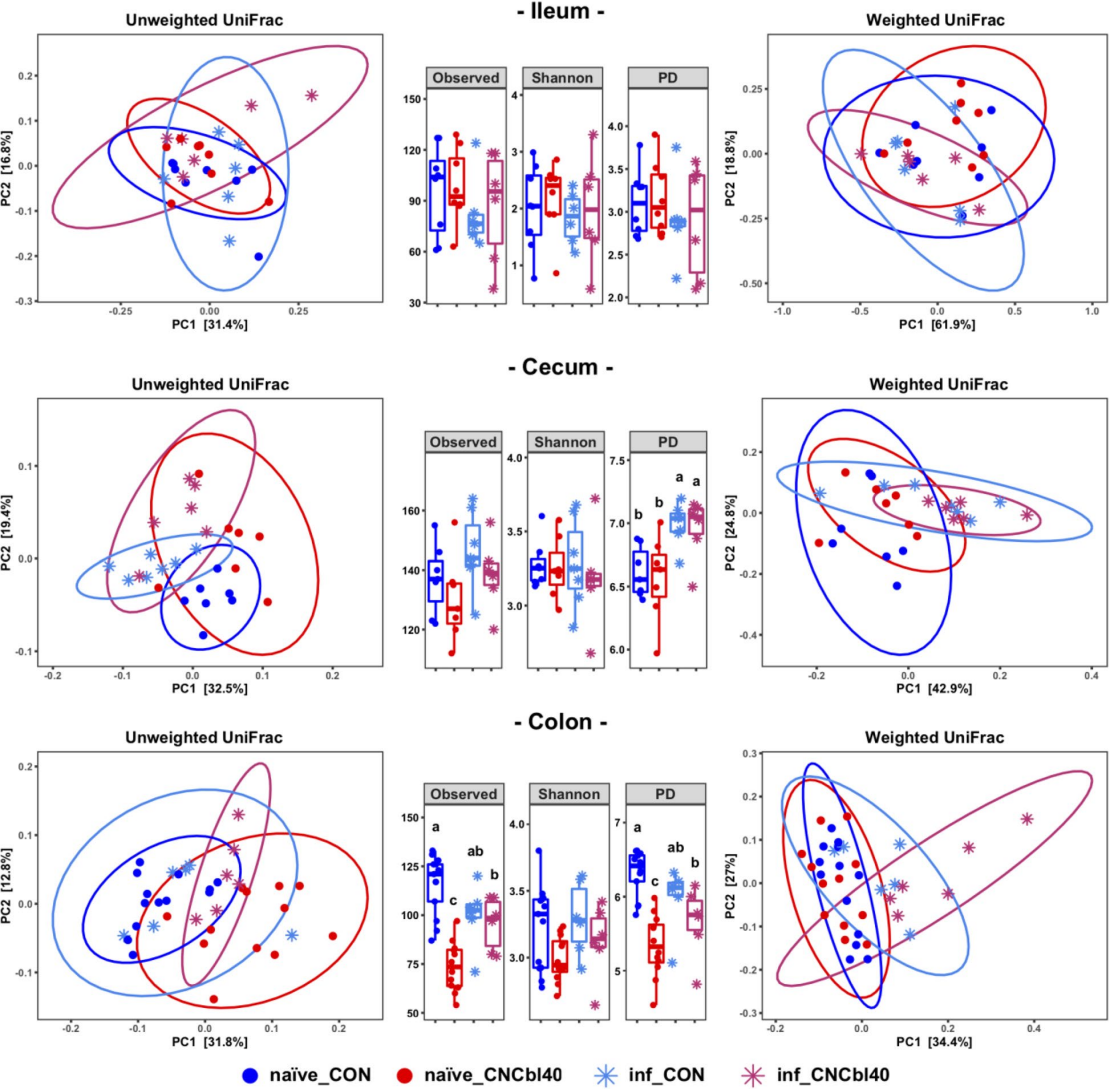
Microbiota analyses of naïve and infected (inf_) mice from the EPC experiment. Principal coordinate plots of the ileum, cecum, and colon microbiota are based on the weighted and unweighted UniFrac dissimilarity metric. Distinct clustering was determined by unweighted UniFrac in the cecum and colon (Additional file 1 Table S2). Cyanocobalamin supplementation significantly reduced alpha diversity (observed and PD) in the colon (*n* = 7–12; *P* < 0.05)

**Fig. 3:**
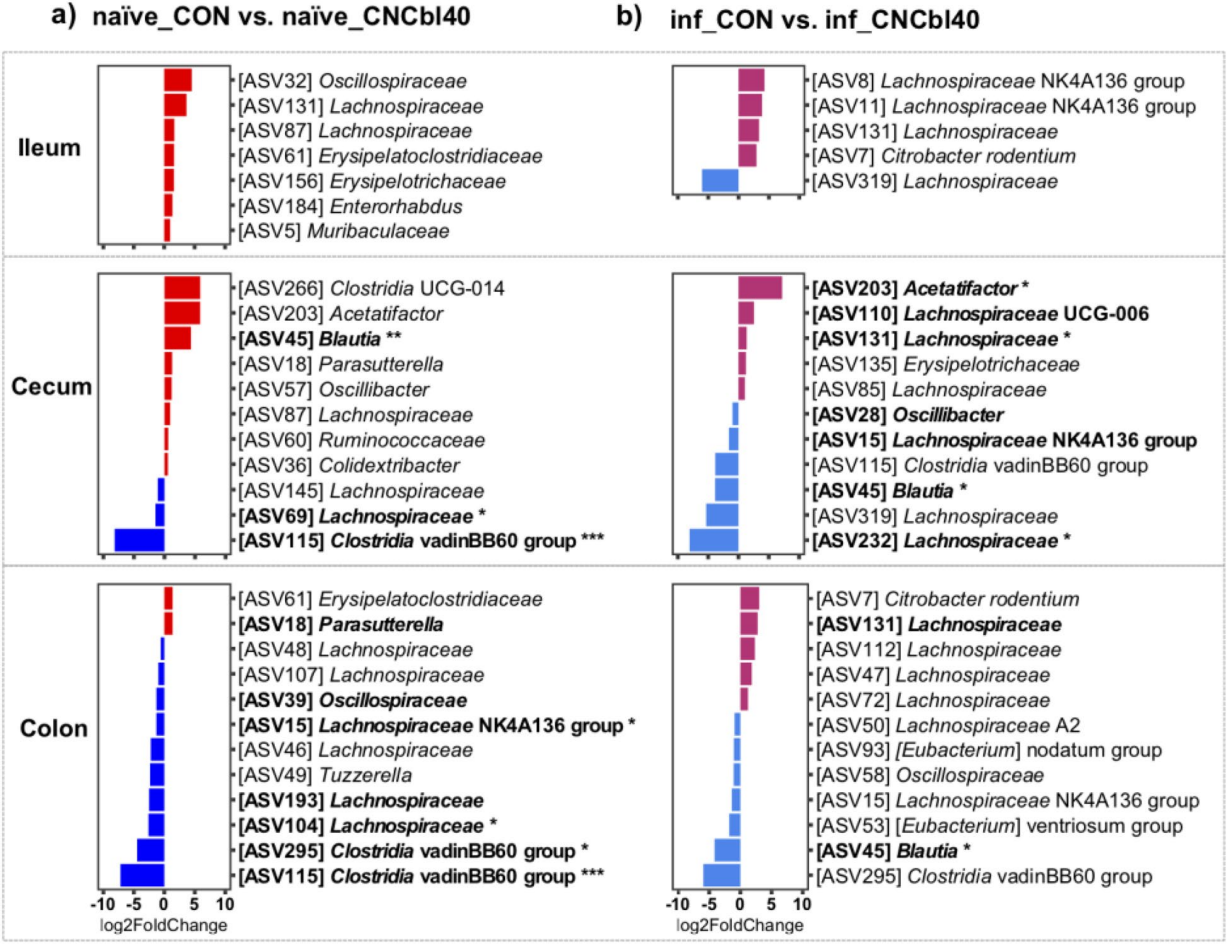
DESeq2 differential analyses of microbial communities in mice from the EPC experiment. Cyanocobalamin supplementation altered the *Firmicutes* population throughout the GI tract. The control group had a greater abundance of *Firmicutes*, including *Lachnospiraceae* species and *Clostridia vadinBB60* group bacterium in the cecum and colon **a** preinfection and **b** postinfection (*n* = 6–8; only ASVs with a *P*-value less than 0.05 were plotted; adjusted *P*-value was used for significance; bolded taxa represent a trend (*P* < 0.10); *P < 0.05, **P < 0.01, *** *P* < 0.001)

Cyanocobalamin supplementation at 40 μg/ml in drinking water caused a shift in microbial composition in the cecum and colon, but not ileum, favoring *Proteobacteria* species and altering the dynamics of the low-abundance *Firmicutes* (Figs. 2 and 3). Species richness, indicated by observed counts, and phylogenetic distance analyses showed that B12 treatment led to lower colonic diversity prior to pathogen challenge. Principal coordinate analysis (PCoA) plots using weighted and unweighted UniFrac distance metrics showed distinct clustering of microbiota in naïve and infected mice (pairwise Adonis; Additional file 1 Table S2). Differences in unweighted UniFrac, but not weighted UniFrac, revealed that low-abundance community members were impacted in the cecum (*P* < 0.05) and colon (*P* < 0.01) in naïve_CNCbl40 mice compared with naïve_CON mice. The severity of microbial disruption induced by infection was more pronounced in the inf_CNCbl40 as compared to the inf_CON group. Both weighted (*P* < 0.01) and unweighted (*P* < 0.05) UniFrac metrics revealed a difference between naïve_CNCbl40 and inf_CNCb140 in the colon, whereas no difference was observed between infected and uninfected control groups (weighted *P* = 1.0, unweighted *P* = 0.81). In the cecum, the gut microbial communities between naïve_CON and inf_CON were different (*P* < 0.01) based on unweighted UniFrac metric, whereas naïve_CNCbl40 and inf_CNCbl40 groups were different (*P* < 0.01) by weighted UniFrac metric.

Differences in beta diversity observed in the colon and cecum were largely explained by changes in *Firmicutes* and *Proteobacteria* populations in both naïve and infected mice as determined by DEseq2’s differential expression analysis (Fig. 3). In advance of infection, an uncultured bacterium belonging to the *Clostridia vadinBB60* group and a *Lachnospiraceae* was significantly lower in the cecum (*P* < 0.001 and *P* < 0.05, respectively) and colon (*P* < 0.001 and *P* < 0.05, respectively), whereas *Parasutterella* was higher in the colon (*P* < 0.10) of the naïve_CNCbl40 group compared with the naïve_CON group (Fig. 3 a). A *Blautia* bacterium was the only significant microbe that increased (*P* < 0.01) in the cecum but not in the colon of naïve_CNCbl40 group. Consistent with the higher fecal *C. rodentium* counts, mice in the inf_CNCb140 group had a numerically higher number of reads corresponding to *C. rodentium* compared to the inf_CON group in the ileum and colon (Fig. 3 b). In the cecum, *Acetatifactor* and a *Lachnospiraceae* species increased (*P* < 0.05), while *Blautia* and other *Lachnospiraceae* species decreased (*P* < 0.05) in infected mice supplemented with cyanocobalamin. The colon of inf_CNCbl40 mice had lower levels of *Blautia* (*P* < 0.05) as well as numerically lower levels of species belonging to *Clostridia vadinBB60* group, Oscillospiraceae, *Lachnospiraceae* A2, and (*Eubacterium*) groups compared to inf_CON. These results indicate that cyanocobalamin supplementation encourages the growth of *Proteobacteria* (*Parasutterella* and *Citrobacter*) species and impacts the dynamics of the low-abundance *Firmicutes* species in the gut.

**Fig. 4:**
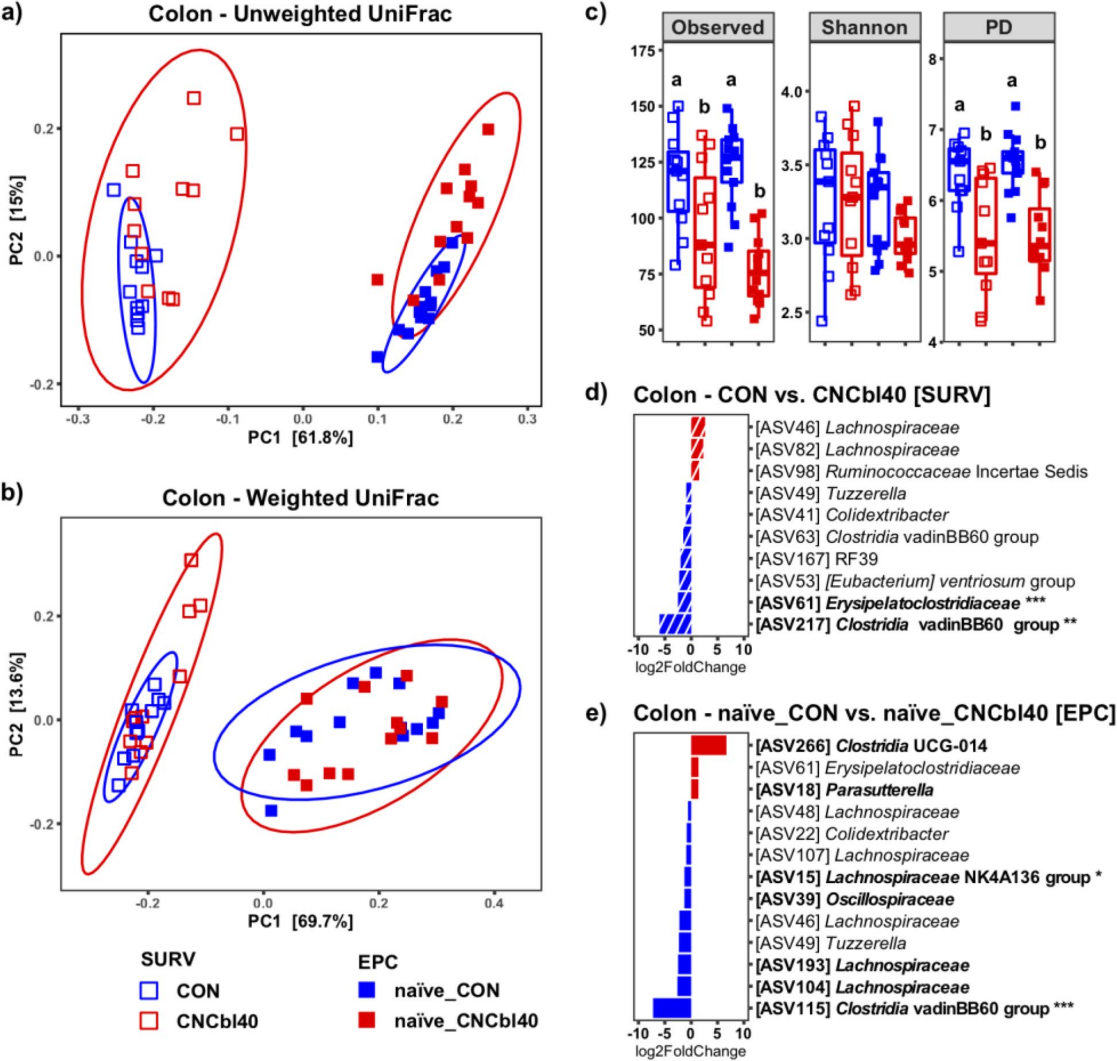
Comparison of the colonic microbial communities in naïve C3H/HeOuJ mice from the SURV and EPC experiments. **a** Unweighted UniFrac PCoA plot and **b** weighted UniFrac comparison show a similar pattern in microbial community clustering (Additional file 1 Table S2). **c** The changes were associated with a consistent reduction in alpha diversity (Observed and PD) regardless of experiment (*n* = 10–12; *P* < 0.05). Differential expression determined by DEseq2 analysis also showed that the *Firmicutes* populations (*Clostridia vadinBB60* group) were impacted by cyanocobalamin in both mice harboring **d** SURV and **e** EPC gut microbiota (*n* = 10–12; bolded taxa represent a trend (*P* < 0.10; *P < 0.05, **P < 0.01, ***P < 0.001)

Consistent shifts in beta diversity in response to B12 supplementation were observed in the SURV and EPC experiments prior to pathogen challenge (Fig. 4). However, there were notable differences in community composition between experiments including the absence of *Akkermansia muciniphila*, *Bacteroides thetaiotaomicron*, and *Parasutterella* species in the SURV experiments (Additional file 1 Fig. S1), which are relevant to the immune phenotypes discussed below. SCFA analysis on cecal content from the SURV experiment revealed that B12 supplementation had no impact on the SCFA concentrations (Additional file 1 Fig. S2). Despite differences in baseline microbiota, the species richness (observed counts) and phylogenetic diversity index were lower (*P* < 0.05) in mice supplemented with B12 in both experiments (Fig. 4 c). In addition to numerically lower levels of *Tuzzerella* species, the *Clostridia vadinBB60* group was consistently reduced (*P* < 0.01) by B12 supplementation (Fig. 4 d and e). Due to the absence of *Parasutterella* in the SURV experiment, the higher relative abundance of the genus (*P* < 0.10) was only detected in the EPC experiment.

**Fig. 5:**
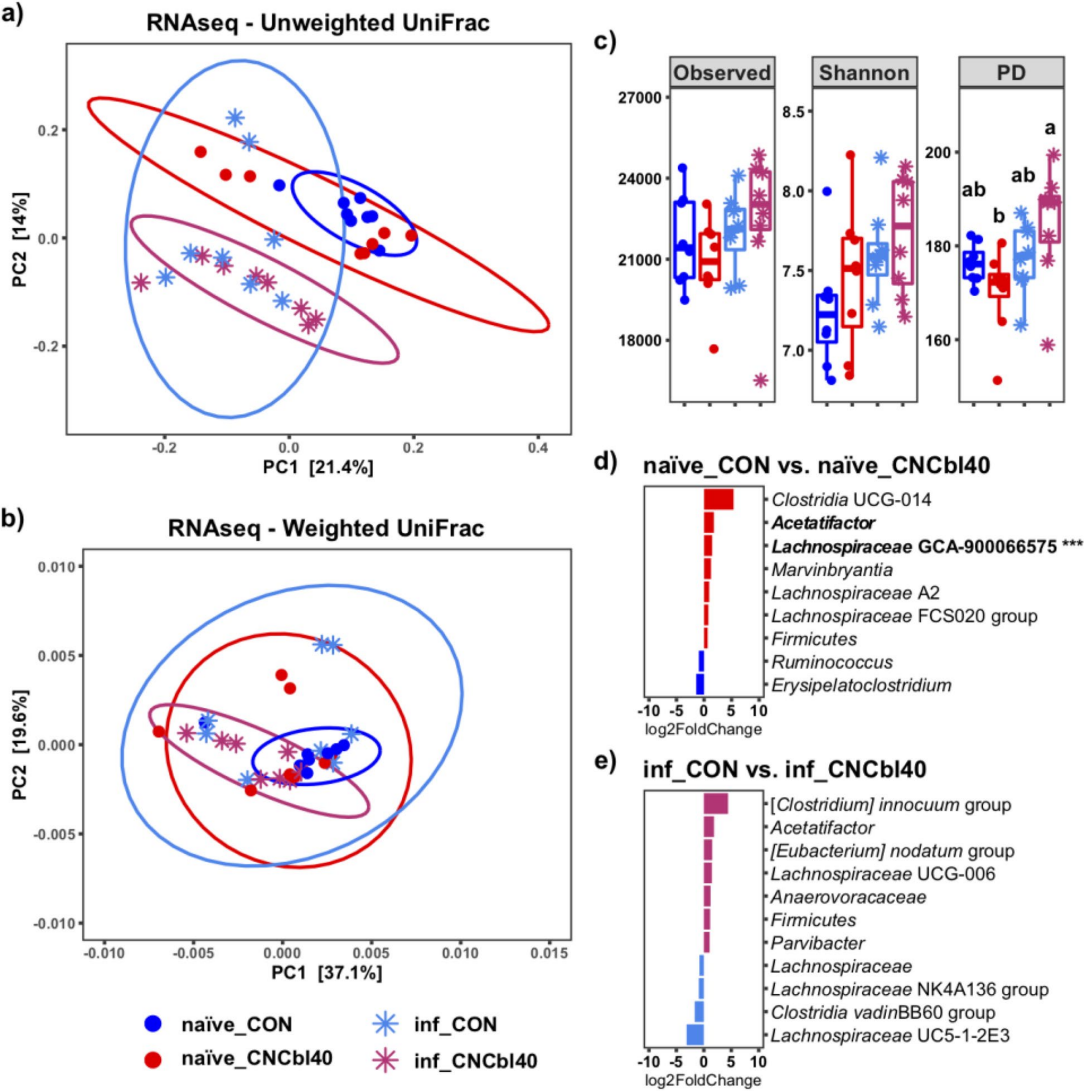
Cecal microbiota analysis of the 16S rRNA gene identified by SAMSA2 metatranscriptome analysis from the EPC experiment. Distinct clustering of microbial communities in **a** unweighted but not **b** weighted UniFrac PCoA plots (Additional file 1 Table S2). **c** Alpha diversity as determine by Observed, Shannon, and PD metrics showed that diversity (PD only) increased postinfection for cyanocobalamin-supplemented mice compared to control (*P* < 0.05). Differential expression analysis using DEseq2 of microbial taxa confirmed that the *Firmicutes* populations were altered from cyanocobalamin supplementation in **d** naïve and **e** infected mice (*n* = 6–8; bolded taxa represent a trend (*P* < 0.10); ***P < 0.001)

We analyzed changes in the active cecal microbiota using ribosomal RNA sequences identified in the metatranscriptome data (Fig. 5). Unweighted UniFrac showed a clear separation between the microbiota of naïve treatment groups and inf_CNCbl40 group, whereas some mice from the inf_CON group remained similar to the naïve treatment groups (Fig. 5 a; Additional file 1 Table S2). No difference was observed with the weighted UniFrac analysis (Fig. 5 b). Alpha diversity metrics revealed no change in observed counts or Shannon diversity indices. However, the PD metric was numerically lower in the naïve_CNCbl40 group, which was consistent with the 16S rRNA gene amplicon datasets moving from the cecum to colon. Interestingly, the inf_CNCbl40 group became more diverse (*P* < 0.05) than the naïve_CNCbl40 group as determined by the PD metric (Fig. 5 c). Overall, changes in community composition with B12 supplementation and infection, including *Clostridia vadinBB60* and *Acetatifactor*, are consistent with 16S rRNA gene amplicon data (Fig. 5 d and e). Differential expression analysis of the active microbial community in the cecum did not reveal significant changes, however, does suggest restructuring of population dynamics in relation to B12 supplementation (Additional file 1 Fig. S3).

### Cyanocobalamin supplementation altered the Firmicutes population dynamics in the cecum

**Fig. 6:**
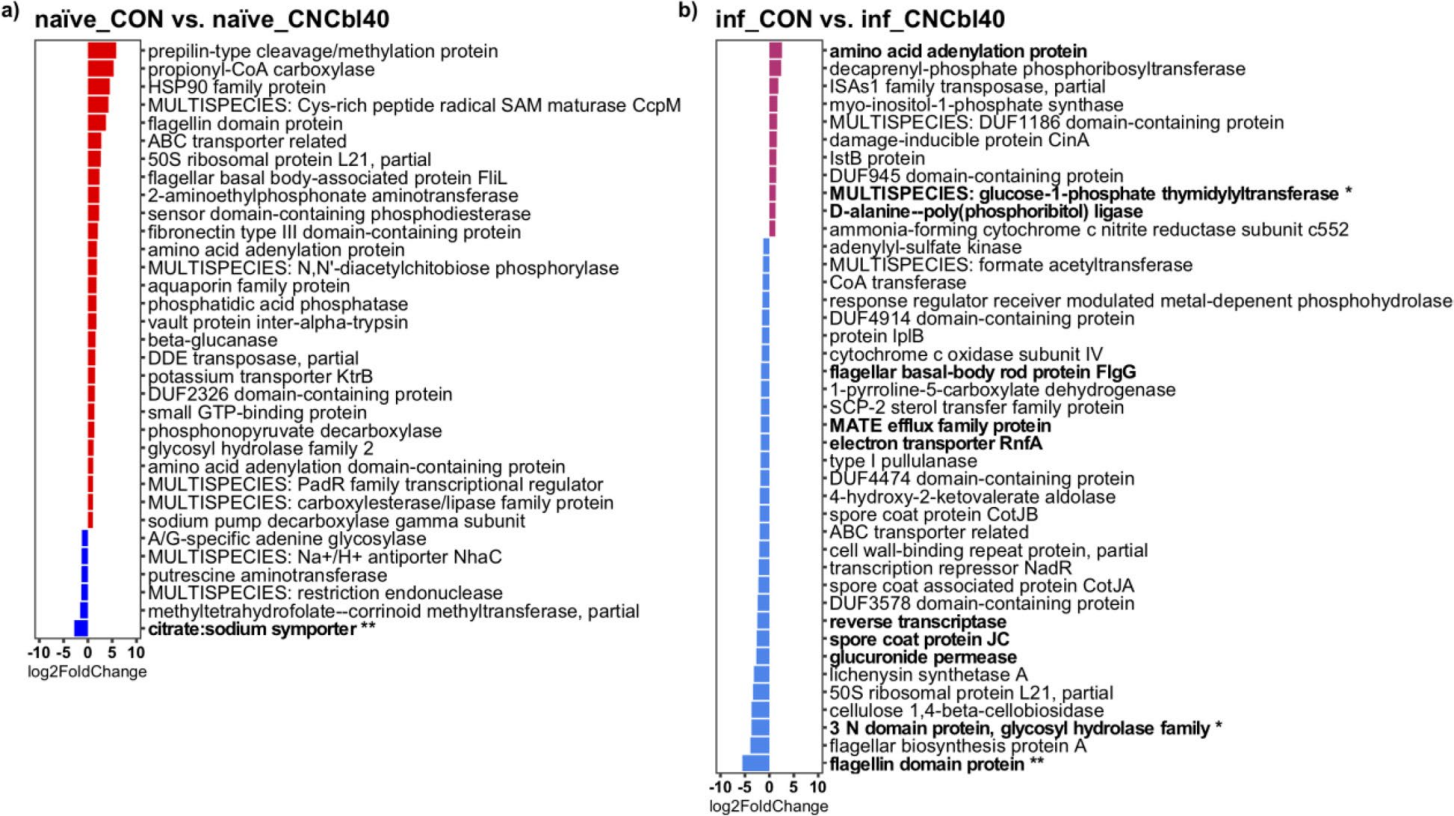
DESeq2 differential expression analyses of cecal metatranscriptome from the EPC experiment. **a** Naïve and **b***C. rodentium*-challenged mice (inf_) supplemented with cyanocobalamin displayed altered functional activities related to metabolism (citrate:sodium symporter) and motility (flagellin domain protein) and confirmed to be from the *Lachnospiraceae* family (*n* = 8; bolded taxa represent a trend (*P* < 0.10); *P < 0.05, **P < 0.01)

**Fig. 7:**
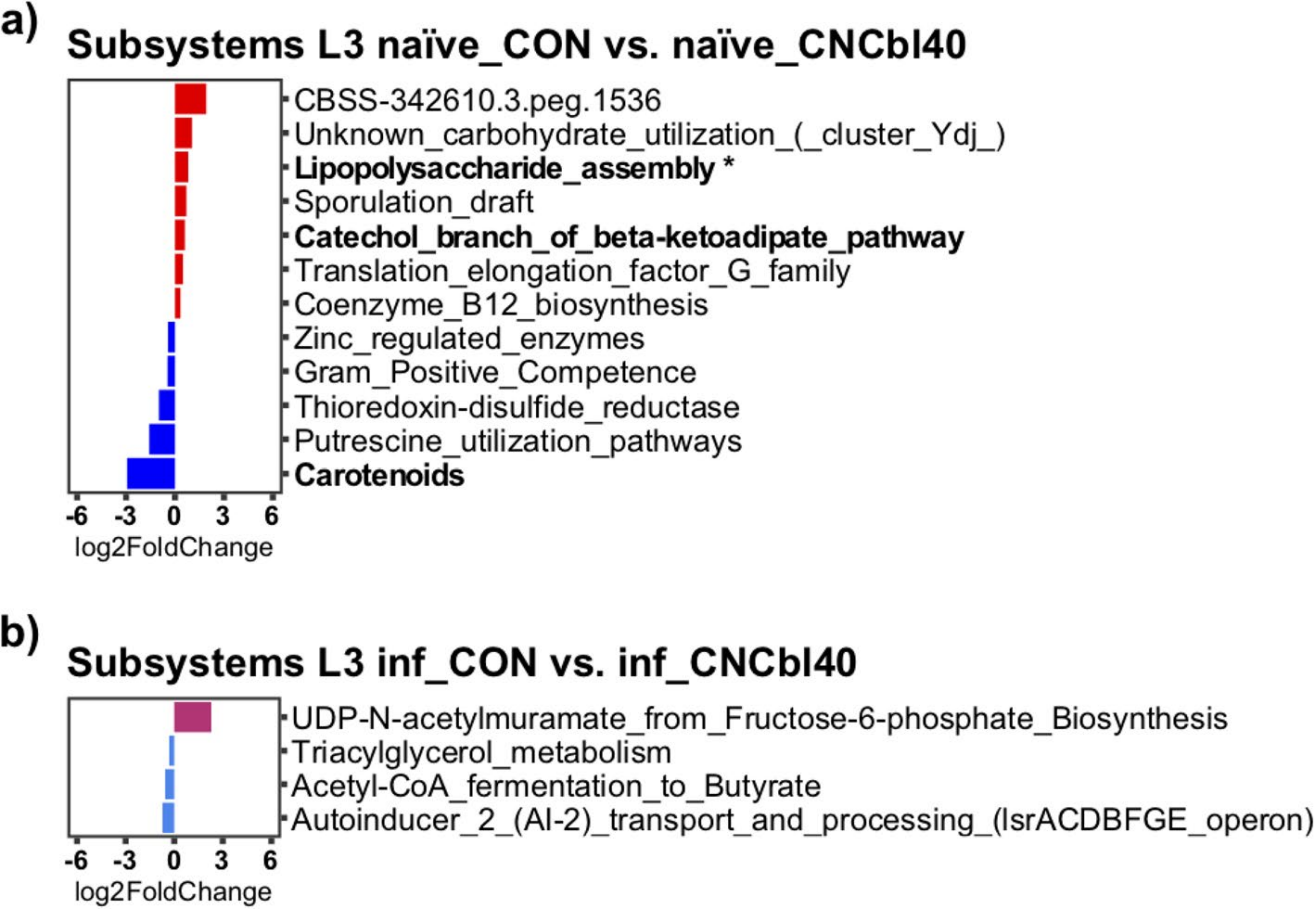
SEED subsystems level 3 DESeq2 analysis of the C3H/HeOuJ cecal metatranscriptome from the EPC experiment. Cyanocobalamin supplementation increased pathways related to gram-negative lipopolysaccharide assembly while decreasing genes related to the competence of gram-positive bacteria in **a** naïve mice but not in **b***C. rodentium*-challenged mice (*n* = 8; bolded taxa represent a trend (*P* < 0.10); *P < 0.05)

Transcriptome analysis revealed that cyanocobalamin treatment led to changes in overall microbial activity pre- and post-pathogen challenge (Fig. 6). The naïve_CNCbl40 group had significantly lower expression of citrate:sodium symporter (*P* < 0.01) and a noteworthy decrease in methyltetrathydrofolate-corrinoid methyltransferase (unadjusted *P* < 0.001, adjusted *P* = 0.64), a cobalamin-specific enzyme (Fig. 6 a). Post-pathogen challenge revealed that the inf_CNCbl40 group had lower expression of flagellin domain protein (*P* < 0.01), 3N domain protein-glycosyl hydrolase family (*P* < 0.05), and reverse transcriptase (*P* < 0.10), whereas enzymes glucose-1-phosphate thymidylyltransferase (*P* < 0.01) and D-alanine-poly (phosphoribitol) ligase (*P* < 0.10) were enriched (Fig. 6 b). The SEED subsystems pathway analysis (level 3) showed a trend for increased expression of genes related to the carotenoid pathway (*P* < 0.10) in the naïve_CON group (Fig. 7). At the same time, mice supplemented with B12 had greater expression of genes related to the lipopolysaccharide assembly (*P* < 0.05) and catechol branch of beta-ketoadipate (*P* < 0.10) pathways. Genes related to gram-positive competence and putrescine utilization pathways were numerically higher in the naïve_CON group. In addition, coenzyme B12 biosynthesis pathways were favored in the naïve_CNCbl40 group (Fig. 7 a). The main pathways enriched in the inf_CON group were related to triacylglycerol metabolism, acetyl-CoA fermentation to butyrate, and autoinducer 2 (A1-2) transport and processing (lsrACDBFGE) pathways. The inf_CNCbl40 group displayed microbial activity that favored the UDP-N-acetylmuramate from fructose-6-phosphate biosynthesis pathway (Fig. 7 b).

**Fig. 8:**
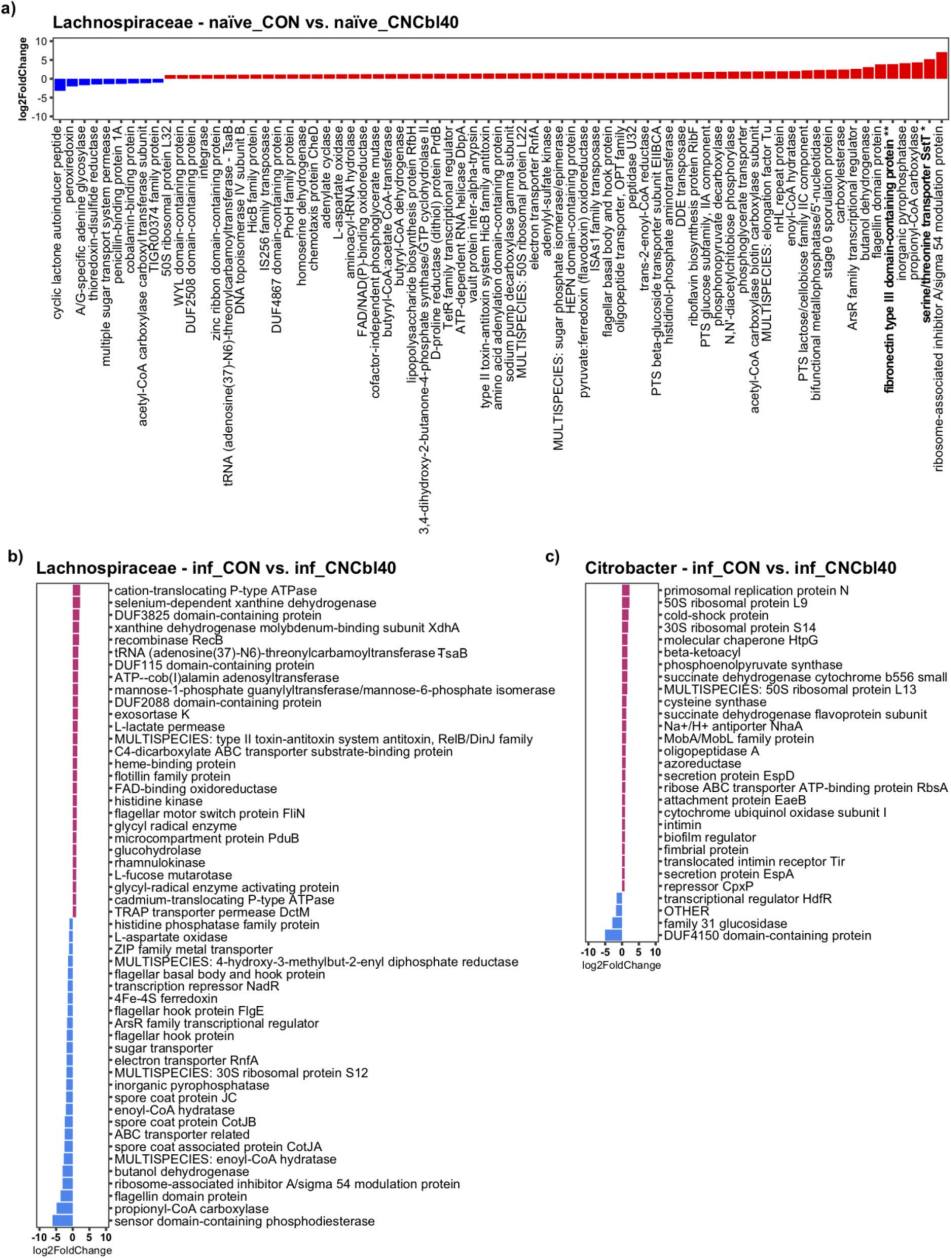
DESeq2 differential expression analyses of *Lachnospiraceae* and *Citrobacter*-specific functional activity from the EPC experiment. Prior to infection, the functional activity of the *Lachnospiraceae* family (**a**) shows that cyanocobalamin treatment increased the expression of numerous genes, including fibronectin type 3 domain-containing protein and serine/threonine transport SstT. **b** After exposure to *C. rodentium*, the *Lachnospiraceae* family members of the inf_CNCbl40 group displayed distinct activities compared to inf_CON group. *Citrobacter*-specific gene expression (**c**) was more pronounced in the inf_CNCbl40 group than inf_CON with notable signals of increased virulence gene expression, while control mice had more family 31 glucosidase activity (*n* = 8; bolded taxa represent a trend (*P* < 0.10); *P < 0.05, **P < 0.01)

The majority of these changes were attributed to *Lachnospiraceae* species preinfection and *C. rodentium* postinfection. Therefore, we extracted and compared the metatranscriptome data annotated to *Lachnospiraceae* and *Citrobacter* (Fig. 8). *Lachnospiraceae* species drastically altered their activity in response to a gut environment saturated with cyanocobalamin. In naïve mice, cyanocobalamin treatment enhanced the expression of fibronectin type 3 domain-containing protein (*P* < 0.01) and serine/threonine transporter SstT (*P* < 0.05), along with favoring numerous notable genes: type 2 toxin-antitoxin system HicB family antitoxin, flagellin domain protein, inorganic pyrophosphatase, propionyl-CoA carboxylase, and ribosome-associated inhibitor A/sigma 54 modulation protein (Fig. 8 a). In addition, cobalamin-binding protein and cyclic lactone autoinducer peptide genes of Lachnospiraceae were favored in the naïve_CON group (Fig. 8 a). Lachnospiraceae species were more active in the inf_CON group than in the inf_CNCbl40 group, with a notable increase in ribosome-associated inhibitor A/sigma 54 modulation protein, flagellin domain protein, propinyl-CoA carboxylase, and sensor domain-containing phosphodiesterase. Of particular interest, ATP-cob(I) alamin adenosyltransferase and MULTISPECIES: type 2 toxin-antitoxin system antitoxin, RelB/DinJ family genes were enriched in the inf_CNCbl40 group (Fig. 8 b). Cyanocobalamin supplementation changed the activity of C. rodentium and notably enhanced expression of numerous virulence genes: E. coli secretion protein A (EspA) and D (EspD), translocated intimin receptor (Tir), intimin, and E. coli attaching and effacing gene B (EaeB) (Fig. 8 c). Interestingly, the gene related to glucose utilization, family 31 glucosidase, was favored in the inf_CON group along with transcriptional regulator HdfR and DUF4150 domain-containing protein (Fig. 8 c).

### Excess cyanocobalamin does not directly impact *C. rodentium* virulence expression in vitro

**Fig. 9:**
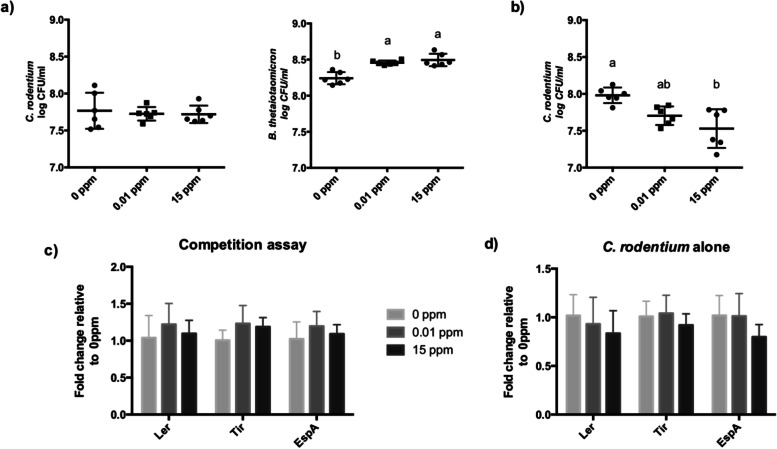
In vitro competition assay of *C. rodentium* and *B. thetaiotaomicron* under physiological relevant concentrations of cyanocobalamin. Cyanocobalamin alone was unable to directly alter *C. rodentium* growth or virulence. **a** Enumeration of *C. rodentium* and *B. thetaiotaomicron* grown in competition for 6 h anaerobically and **b** of *C. rodentium* grown alone. Expression of gene related to virulence (Ler, Tir, and EspA) did not differ between treatments when *C. rodentium* was grown in **c** competition or **d** alone at different concentrations of cyanocobalamin (*n* = 6; *P* < 0.05)

Previous studies have shown altered virulence expression of Shiga toxin-producing EHEC in vitro in response to *B. thetaiotaomicron* competition for cobalamin [12, 29]. Therefore, we tested *C. rodentium* virulence expression when grown alone or in competition at physiologically relevant concentrations of cobalamin (Fig. 9). Because luminal cobalamin levels increased from 0.01 ppm to approximately 15 ppm with supplementation, we evaluated how cyanocobalamin at 0 ppm, 0.01 ppm, and 15 ppm impacted *C. rodentium* growth and virulence. The competition assay revealed that *C. rodentium* maintains steady levels regardless of cobalamin exposure. In contrast, *B. thetaiotaomicron* numbers increased (*P* < 0.05) with additional cyanocobalamin at 0.01 and 15 ppm (Fig. 9 a), indicating that there was competition for B12. When *C. rodentium* was grown alone, cyanocobalamin treatment lowered *C. rodentium* abundance from 8.0 to 7.5 log CFU/ml (*P* < 0.05) in a dose-dependent manner (Fig. 9 b). Virulence factors of the locus of enterocyte effacement (LEE), which include the LEE-encoded regulator (Ler), Tir, and EspA, were not found to be different in the competition assay (Fig. 9 c and d) or when *C. rodentium* was grown alone (Fig. 9 d).

### Cobalamin supplementation alters colonic cytokine profiles of naïve and infected C3H/HeOuJ mice

**Fig. 10:**
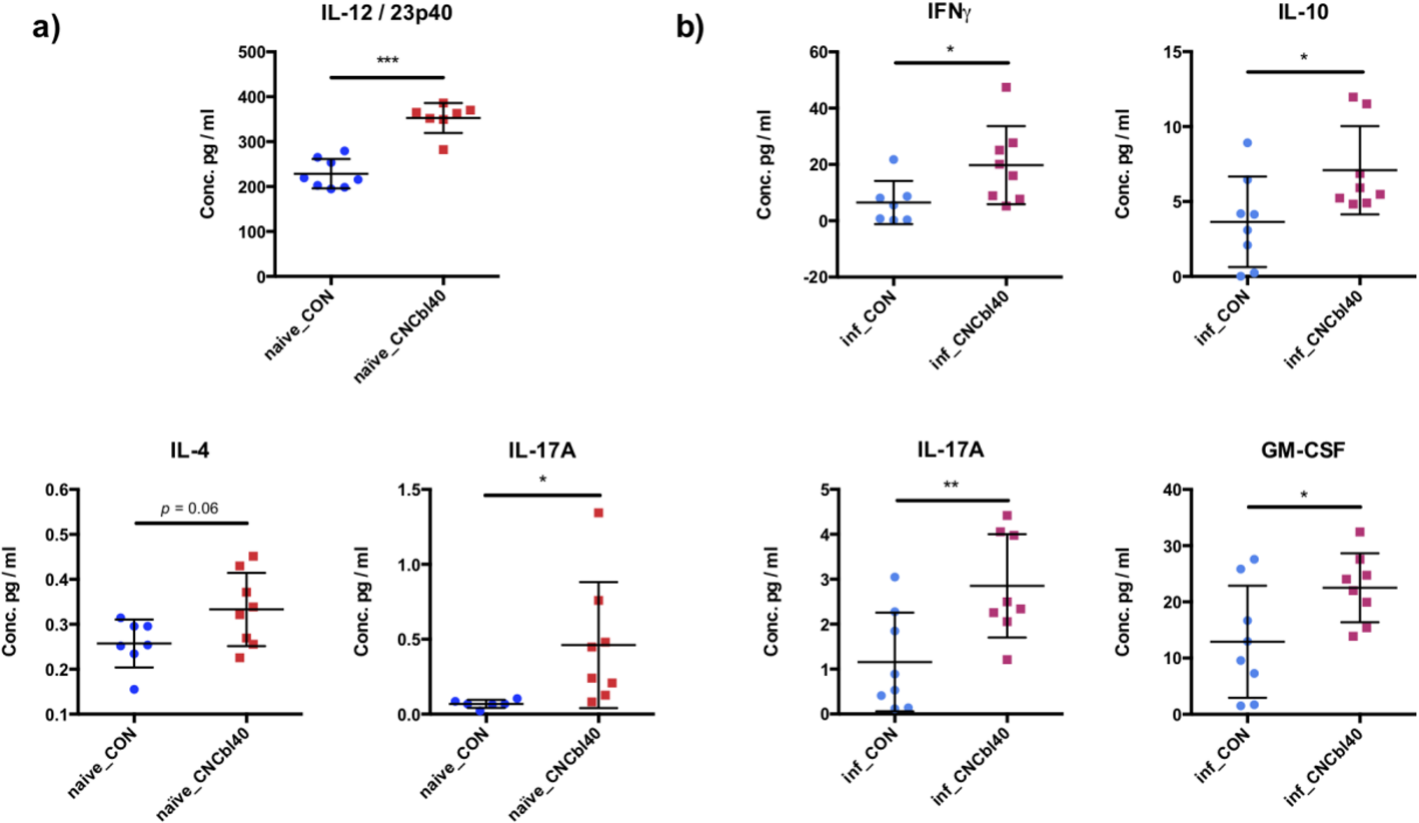
Significant host biomarker concentrations determined in colon tissue homogenates of C3H/HeOuJ mice from the EPC experiment. **a** Cyanocobalamin supplementation enhanced IL-12/23p40 and IL-17A cytokines of naïve mice, with a higher trend of IL-4 levels (*P* = 0.06) compared to control. **b***C. rodentium* challenge led to a significant increase in IFNγ, IL-10, IL-17A, and GM-CSF in the cyanocobalamin-supplemented mice (*n* = 8; *P < 0.05, **P < 0.01, ***P < 0.001)

To determine host response to cyanocobalamin treatment, cytokine and chemokine biomarkers were measured in the colon tissue of naïve and infected mice of the EPC experiment (Fig. 10; Additional file 1 Figs. S4 & S5). Naïve mice supplemented cobalamin in excess had greater concentrations of cytokines IL-12/23p40 (*P* < 0.001), IL-4 (*P* = 0.06), and IL-17A (*P* < 0.05) in colon tissue (Fig. 10 a). The inf_CNCbl40 group had increased levels of IFNγ (*P* < 0.05), IL-10 (*P* < 0.05), IL-17A (*P* < 0.01), and GM-CSF (P < 0.05) compared to inf_CON group (Fig. 10 b). These results suggest that cyanocobalamin supplementation impacted immune activation in advance of infection.

### Colonic p40 subunit increased with cobalamin treatment depending on microbiota status

**Fig. 11:**
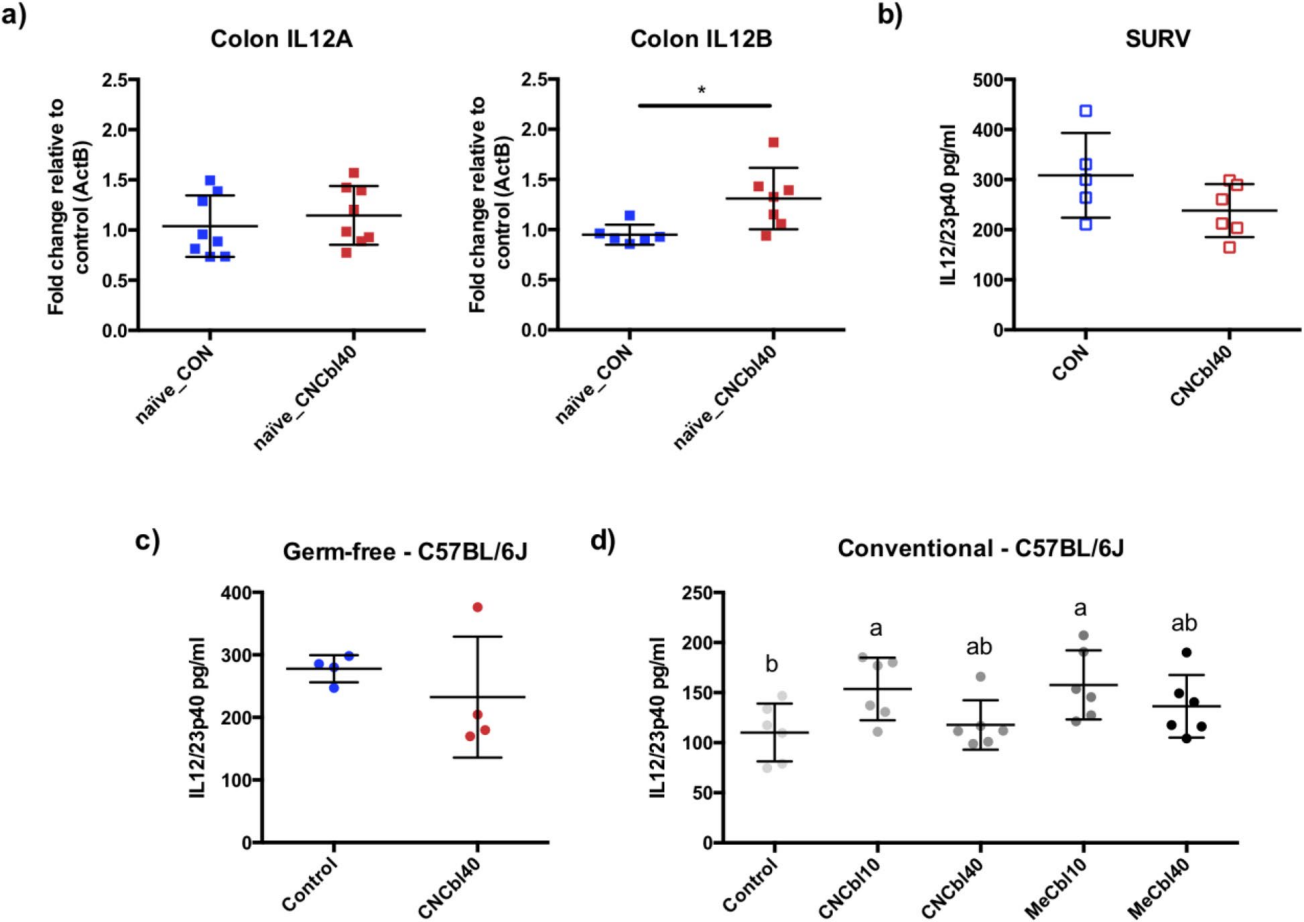
Cyanocobalamin-induced dysbiosis increased the production of the p40 subunit in colon tissues of mice prior to pathogen challenge. Pre-infection levels of IL-12/23p40 in colon tissues were directly related to microbiota structure and independent of related mice genotypes. **a** Gene expression was significantly higher for IL12B, the p40 subunit coding gene, but not for the IL-12A (p35) gene (*n* = 8) in the EPC experiment. **b** The SURV experiment did not display the increase in IL-12/23p40 (*n* = 5–6), nor did **c** germ-free C57BL/6J mice (*n* = 4; *P < 0.05, **P < 0.01, ***P < 0.001). **d** Cyanocobalamin and methylcobalamin supplementation at 10 μg/ml and 40 μg/ml increased IL/23p40 protein levels in the colon of conventional C57BL/6J mice (*n* = 6; *P* < 0.05) compared to control

The IL-12/23p40 measurement could represent either the IL-12 or IL-23 cytokines. Therefore, we measured gene expression of IL12A (p35) and IL12B (p40) in the colon. IL12B was significantly enriched (*P* < 0.01) in the naïve_CNCbl40 group compared the naïve_CON group, but no difference was observed with IL12A (Fig. 11 a). In agreement, IL-12p70 cytokine levels, a heterodimer of p35 and p40 subunits, were below the assay’s detection limit in the colon (data not shown). The increase in IL-12/23p40 levels was not observed in the SURV experiment mice (Fig. 11 b), indicating the response is likely dependent on a specific component of the microbiota. As such, we tested whether a microbial community was required for the induction of IL-12/23p40. Indeed, cyanocobalamin treatment did not increase IL-12/23p40 protein levels in germ-free C57BL/6J mice (Fig. 11 c), although it did increase in conventional C57BL/6J mice (Fig. 11 d). Correlative analyses in SURV and EPC revealed that IL-12/23p40 is associated with a reduction in multiple *Firmicutes* species, including *Lachnospiraceae*, Oscillospiraceae, Eggerthellaceae, and Ruminococcaceae members in naïve mice (Additional file 1 Fig. S6). Select *Ruminococcaceae* species negatively and positively correlated with IL-12/23p40 in infected mice (Additional file 1 Fig. S6). We also wished to rule out the potential of cyanide as compared to other forms of B12. Conventionalized C57BL/6J mice displayed similar increases in IL-12/23p40 protein levels when they received either cyanocobalamin or methylcobalamin. Colon IL-12/23p40 protein levels were greatest in the CNCbl10 (*P* < 0.05) and MeCbl10 (*P* < 0.05) groups, while CNCbl40 and MeCbl40 were numerically higher (Fig. 11 c).

## Discussion

We demonstrate that excessive cobalamin levels in the gut can alter the functional dynamics of the microbiota and host immune signaling, providing an environment supportive of pathogen colonization. It is well established that host metabolism, including host diet, environmental factors, and immune status, drives the ecological environment of the GI tract [33–35]. Perturbations in activities of the gut microbiota, otherwise known as a gut dysbiosis, are typically described along with several diseases [36, 37]. A study using antibiotic treatment to deplete butyrate-producing microbes revealed that this type of dysbiosis supports *Enterobacteriaceae* expansion and inflammation through suppression of the peroxisome proliferator-activated receptor gamma (PPAR-γ) homeostatic signaling pathway [38]. The activation of PPAR-γ has been shown to prevent gut inflammation by regulating macrophage and T-cell populations [39, 40]. Many studies using antibiotics and diet to induce a dysbiosis have associated a decrease in alpha and beta diversity to increased mucosal inflammation and a shift in pathogen and/or pathobiont activity [41–44]. Environmental factors and cooperative-metabolic strategies in the host can promote host-microbiota mutualism and promote the shift of a virulent pathogen to an avirulent passenger of the gut [45]. In the present study, the effect of high cobalamin levels on the gut microbiota prior to pathogen challenge helps to explain the enhanced *C. rodentium* colonization, reduced survival, and increased mucosal damage in the infected cyanocobalamin-treated mice.

Cyanocobalamin treatment had a subtle impact on overall microbiota structure but a distinct impact on the *Firmicutes* populations in cecum and colon. A study using C57BL/6J mice concluded that B12 supplementation minimally impacts microbial community structure under healthy conditions but does after DSS-induced colitis treatment [46]. Under these conditions, the researchers saw a decrease in *Lachnospiraceae* family members and *Parabacteroides*, along with an increase in *Enterobacteriaceae* species in non-SPF mice receiving a B12-supplemented diet at 200 mg of cyanocobalamin per kilogram [46]. This data supports our findings that naïve mice supplemented with cyanocobalamin consistently showed decreased alpha diversity due to the reduced abundance of *Firmicutes* species and an increase in *Parasutterella* species. Upon *C. rodentium* challenge, we found that mice supplemented with cyanocobalamin had a greater quantity of *C. rodentium* with a notable change to numerous *Lachnospiraceae* species. Cyanocobalamin treatment consistently reduced *Clostridia vadinBB60* group, *Lachnospiraceae* NK4A136 group, and other *Lachnospiraceae* species in both the SURV and EPC experiments. Similar changes to the microbiota were found to increase the ability of *Salmonella* to colonize the gut [47]. Researchers found that gnotobiotic mice harboring greater abundances of *Clostridia vadinBB60* and *Lachnospiraceae* NK4A136 groups, as well as *Ruminococcaceae* members, had enhanced colonization resistance to a *Salmonella* challenge [47]. In humans, high levels of *Clostridia vadinBB60* have also been associated with low *Escherichia-Shigella* [48]. The interactions among these *Firmicutes* and other commensal microbes have been shown to contribute to a host’s ability to resist pathogen colonization and pathogenesis in *Salmonella*, *Clostridium difficile*, EHEC, and *Citrobacter* infections [49–53]. Therefore, we looked at the activity of the cecal microbiota in naïve and infected mice to better understand the impact of cyanocobalamin on the integration of *C. rodentium* into the resident gut community.

The activity of the gut microbiome, as determined through cecal metatranscriptome, was altered by cyanocobalamin treatment, indicating that cobalamin transport and utilization may be altered in some microbes. The citrate:sodium symporter, which was more expressed in naïve_CON than naïve_CNCbl40 group, is present only in a few pathogenic and commensal microbes in the GI tract and is required for citrate fermentation [54]. Interestingly, the increased citrate utilization by the gut commensal and opportunistic pathogen *Enterococcus faecalis* improved their pathogenic behavior in *Galleria mellonella* larvae [54]. Following cyanocobalamin supplementation, the loss in citrate:sodium symporter expression suggested an overall reduction in citrate metabolism by commensal microbes. This would leave more citrate for *C. rodentium* and/or open a niche that is typically filled by citrate-metabolizing microbes in naïve mice. The persistence of commensals in various niches of the gut likely contributes to a host’s ability to resist pathogen colonization through competitive exclusion [55], and the enhanced citrate metabolism could be a good indication of microbe-microbe mutualism. The increased expression of lipopolysaccharide assembly genes in naïve mice implies that gram-negative microbes, such as *Parasutterella*, were more active and higher in abundance with cyanocobalamin supplementation. This was matched by an increase in genes of the catechol branch of beta-ketoadipate pathway, which may be related to the proteobacterial degradation of B12 as previously shown in *Pseudomonas* species [56]. The increase in “coenzyme B12 biosynthesis” was related to genes involved in cobalamin transport, a result of microbes recycling cobalamin when present in excess. In contrast, genes related to “gram-positive competence” were elevated in the control group, along with putrescine utilization and carotenoid pathways. These pathways have been associated with colonic immunity and microbial mutualism that help to maintain GI homeostasis [57–60]. Cyanocobalamin supplementation led to distinct changes to the cecal microbiome, including changes to various enzymes, transcription regulators, and transporters, and may indirectly explain the enhanced colonization of *C. rodentium*. In agreement, culture experiments showed no direct impact on *C. rodentium* growth or virulence at physiological relevant cobalamin levels. Interestingly, *C. rodentium* had enhanced expression of “family 31 glucosidase” in control mice, a feature that may explain the nature of this pathogen’s metabolism and increased virulence gene expression with cyanocobalamin supplementation. In fact, bacterial glucose metabolism and host niche adaptations that increase glucose levels in the intestine have been shown to control pathogen virulence [33] and attenuate virulence in *C. rodentium* [45]*.* Moreover, B12 may directly contribute to *C. rodentium* metabolism through the degradation of 1,2-propanediol, to which the first enzyme in the pathway is a B12-dependent propanediol dehydratase to produce propionate [61]. However, excess B12 had minimal effects on *C. rodentium* growth and virulence in vitro when grown with *B. thetaiotaomicron*, a 1,2-propanediol producer in the large intestine [62]. This suggests the impact of B12 on infection outcome is not likely through the direct utilization of B12 by *C. rodentium* but the overall effects on the resident microbiota, which occurred prior to infection challenge. For instance, a notable change in flagellin domain protein, related to *Lachnospiraceae* species, may represent a greater necessity for these organisms to be motile. This may be connected to fibronectin type 3 domain-containing protein and serine/threonine transporter, both of which have been shown to be important for cell binding [63, 64] and anabolic reactions [65], respectively. We suspect that this is a sign of niche displacement among *Lachnospiraceae* species and likely other *Firmicutes* in response to cyanocobalamin supplementation and pathogen challenge.

The cobalamin-induced changes in microbial composition and activity described above likely contributed to increased IL12/23p40 subunit levels in the colon. The cytokines IL-12 and IL-23 play a central role in T-cell-mediated regulation, and their use as therapeutic targets has highlighted their importance to host defense and inflammatory disease [66–68]. The activation of IL-12p40 has previously been attributed to hypoxia-inducible factor-1, a key regulator of mucosal inflammation by controlling Th1/Th17 response [69]. Contrary to our results, researchers provide evidence of IL12p40 being protective against *C. rodentium*. Still, they noted a decrease in IL-17, which in our mice was elevated in both a naïve and infected cyanocobalamin-supplemented groups. Previous studies have shown that the microbiota influences Th17 response in the gut [70], and that this intestinal inflammation can enhance pathogen colonization [71]. In fact, a study with similar microbial changes to our control mice caused by knocking out the class 1-restricted T-cell-associated molecule gene was associated with lower Th17 response and reduced *Salmonella* colonization [47]. Although IL-17 plays a key role in protecting against *C. rodentium* infection [72], we found that the increased level of IL-17 and IL-12/23p40 in colon tissue prior to infection was a sign of immune dysregulations, and this led to greater colonization. Postinfection, we found greater levels of IFNγ and IL-17A in the colon of mice supplemented with cyanocobalamin. An immune phenotype is previously characterized in the cecum of mice with a resident microbiota dominated by *Bacteroidetes* and *Verrucomicrobiaceae* species that together or in conjunction with other bacteria primes mucosal immunity to help protect against *Salmonella* colonization [50]. The enhanced production of IFNγ and IL-17A in gut tissues was associated with reduced *Salmonella* colonization, whereas in our study, an increase of *C. rodentium* colonization corresponded with higher IL-17A and IL-12/23p40 but with no change in IFNγ. Interestingly, different commensal *E. coli* strains are associated with changes in IFNγ, IL-17A, and GM-CSF production [73]; however, the mice in our study were *E. coli*-free, and this may explain why we did not see an increase in IFNγ production. Germ-free and SURV-experiment mice did not exhibit enhanced IL-12/23p40 protein levels from B12 supplementation as observed in EPC-experiment mice. This further suggests that the effects from cyanocobalamin are mediated through the gut microbiota. Finally, both cyano- and methyl-cobalamin supplementation to conventional C57BL/6J mice at 30 and 120 μg/day similarly increased IL-12/23p40 levels in the colon. Taken together, these results suggest that the B12-induced dysbiosis drives IL-12/23p40 levels in the colon regardless of B12 form and at a typical human equivalent dose (one 5000 μg tablet) of 30 μg/day in mice. Future studies should use lower doses to determine the threshold concentration of B12 in the gut necessary to change the activities of the microbiota, particularly in the *Lachnospiraceae* populations and how these changes impact microbe-host interactions in the gut with a specific focus on IL-12p40 cytokine production along the GI tract.

## Conclusion

In the present study, we showed that excessive B12 concentrations in the gut can induce a dysbiosis that promotes *C. rodentium* colonization and pathogenesis in mice. The B12-induced dysbiosis can be characterized by the decrease in members belonging to *Clostridia vadinBB60* and *Lachnospiraceae* NK4A136 groups, along with an increased abundance of *Parasutterella* in the cecum and colon*.* The changes to the gut microbiota from B12 supplementation stimulated the production of the IL-12/23p40 subunit and IL-17A cytokines in the colon and opened the gut niche for *C. rodentium* colonization without any obvious tissue damage prior to pathogen challenge. In general, oral B12 supplementation can contribute to a dysbiosis that promotes low-grade inflammation and a virulent *C. rodentium* population regardless of cobalamin type. Through changes to the microbiota, a B12-saturated gut environment shifts the metabolism of *C. rodentium* from avirulent to virulent as determined by the decrease in glucose enzyme activity and increase in virulent gene expression.

## Supplementary Information

**Additional file 1: Supplementary Figures S1.** DESeq2 differential expression analysis of the microbial community between naïve C3H/HeOuJ mice from the SURV and EPC experiments. **Figure S2.** Cecal SCFA profiles of naïve C3H/HeOuJ mice from the SURV experiment. **Figure S3.** DESeq2 differential expression analyses of the active microbial community as determine by cecal metatranscriptomics of C3H/HeOuJ mice. **Figure S4.** Inflammation biomarkers in naïve C3H/HeOuJ mice from the EPC experiment (n = 8). **Figure S5.** Inflammation biomarkers in *C. rodentium*-challenged C3H/HeOuJ from the EPC experiment (n = 8). **Figure S6.** Spearman’s correlation of the significantly altered immune profiles of colon tissues and microbiota in C3H/HeOuJ mice. **Supplementary Tables:Table S1.** Real-time qRT-PCR primer list. **Table S2.** Summary of beta-diversity analyses of microbial communities.

**Additional file 2.** Microbiota R analysis.

**Additional file 3.** Microbiota Deseq2 Results.

**Additional file 4.** Cecal metatranscriptomics Deseq2 Results.

## Data Availability

All data generated or analyzed during the current study are included in this published article (and supplementary information files) or available in the NCBI SRA repository, BioProject: PRJNA791318.

## Abbreviations

CON: Control
CNCbl10: Cyanocobalamin at 10 μg/ml
CNCbl40: Cyanocobalamin at 40 μg/ml
MeCbl10: Methylcobalamin at 10 μg/ml
MeCbl40: Methylcobalamin at 40 μg/ml
EHEC: Enterohemorrhagic *Escherichia coli*
SURV: Survival experiment
EPC: Early-stage pathogen colonization experiment
PBS: Phosphate-buffered saline
SAMSA2: Simple annotation of metatranscriptomes by sequence analysis 2.0
SCFA: Short-chain fatty acid
INFγ: Interferon gamma
IL: Interleukins
KC: Keratinocytes-derived chemokine
TNFα: Tumor necrosis factor alpha
GM-CSF: Granulocyte-macrophage colony-stimulating factor
MMP9: Matrix metalloproteinase-9
RANTES: Regulated on activation normal T cell expressed and secreted
IP10: Interferon gamma-induced protein-10
MCP1: Monocyte chemoattractant protein-1
MIP: Macrophage inflammatory proteins
ASV: Amplicon sequencing variant
BHI: Brain heart infusion.
