# Supplementary material for: Over supplementation with vitamin B12 alters microbe-host interactions in the gut leading to accelerated Citrobacter rodentium colonization and pathogenesis in mice
Source: Microbiome. 2023 Feb 3;11:21. doi: 10.1186/s40168-023-01461-w (PMC9896722; doi:10.1186/s40168-023-01461-w)
Supplement: Supplementary file 2 — Additional file 1: Supplementary Figures S1. DESeq2 differential expression analysis of the microbial community between naïve C3H/HeOuJ mice from the SURV and EPC experiments. Figure S2. Cecal SCFA profiles of naïve C3H/HeOuJ mice from the SURV experiment. Figure S3. DESeq2 differential expression analyses of the active microbial community as determine by cecal metatranscriptomics of C3H/HeOuJ mice. Figure S4. Inflammation biomarkers in naïve C3H/HeOuJ mice from the EPC experiment (n = 8). Figure S5. Inflammation biomarkers in C. rodentium-challenged C3H/HeOuJ from the EPC experiment (n = 8). Figure S6. Spearman’s correlation of the significantly altered immune profiles of colon tissues and microbiota in C3H/HeOuJ mice. Supplementary Tables:Table S1. Real-time qRT-PCR primer list. Table S2. Summary of beta-diversity analyses of microbial communities. [file 40168_2023_1461_MOESM1_ESM.docx]

**Supplementary Figures and Tables**


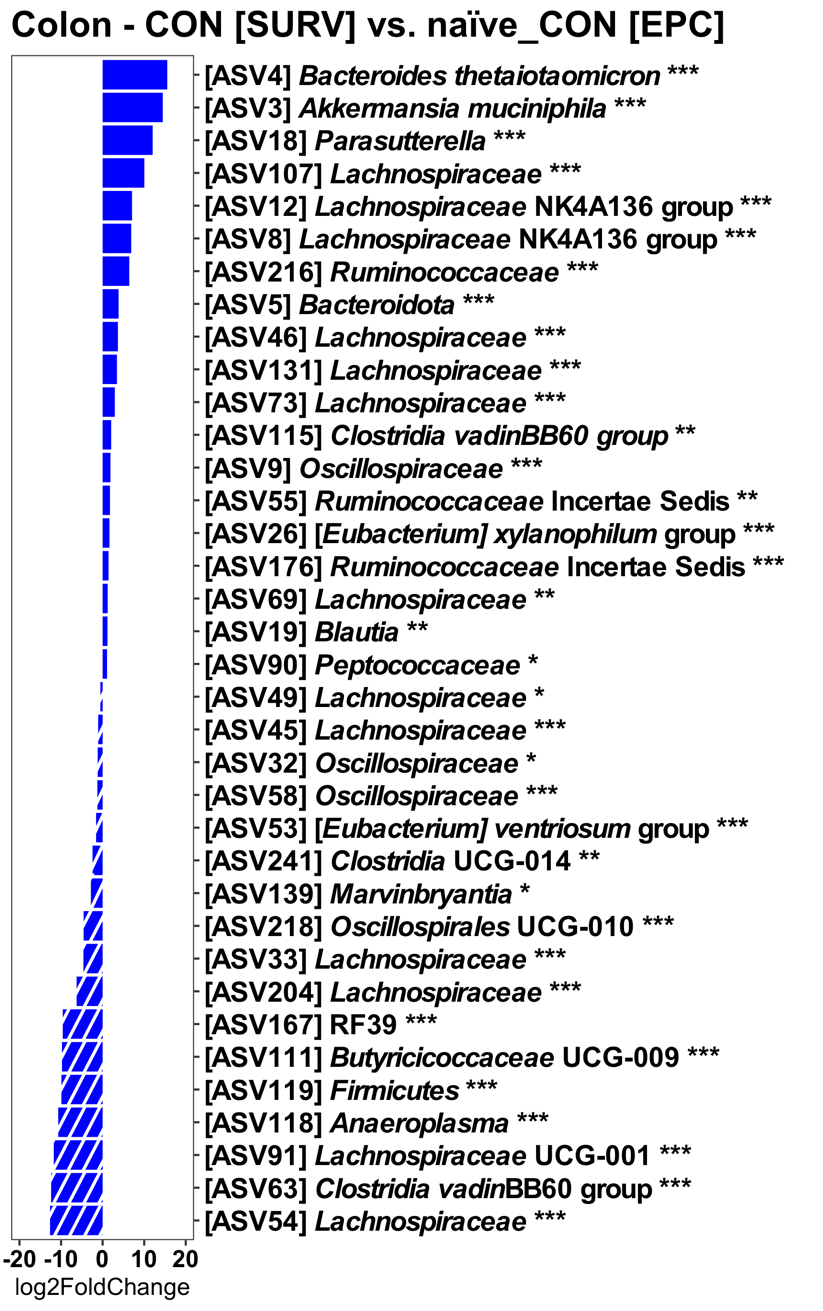


**Figure S1: DESeq2 differential expression analysis of the microbial community between naïve C3H/HeOuJ mice from the SURV and EPC experiments.** The SURV experiment is distinguishable by the complete absence of major taxa such as *Bacteroides thetaiotaomicron, Akkermansia muciniphila* and *Parasutterella* (n = 13-14; bolded taxa represent a trend (*P* < 0.10); * *P* < 0.05, ** *P* < 0.01, *** *P* < 0.001).


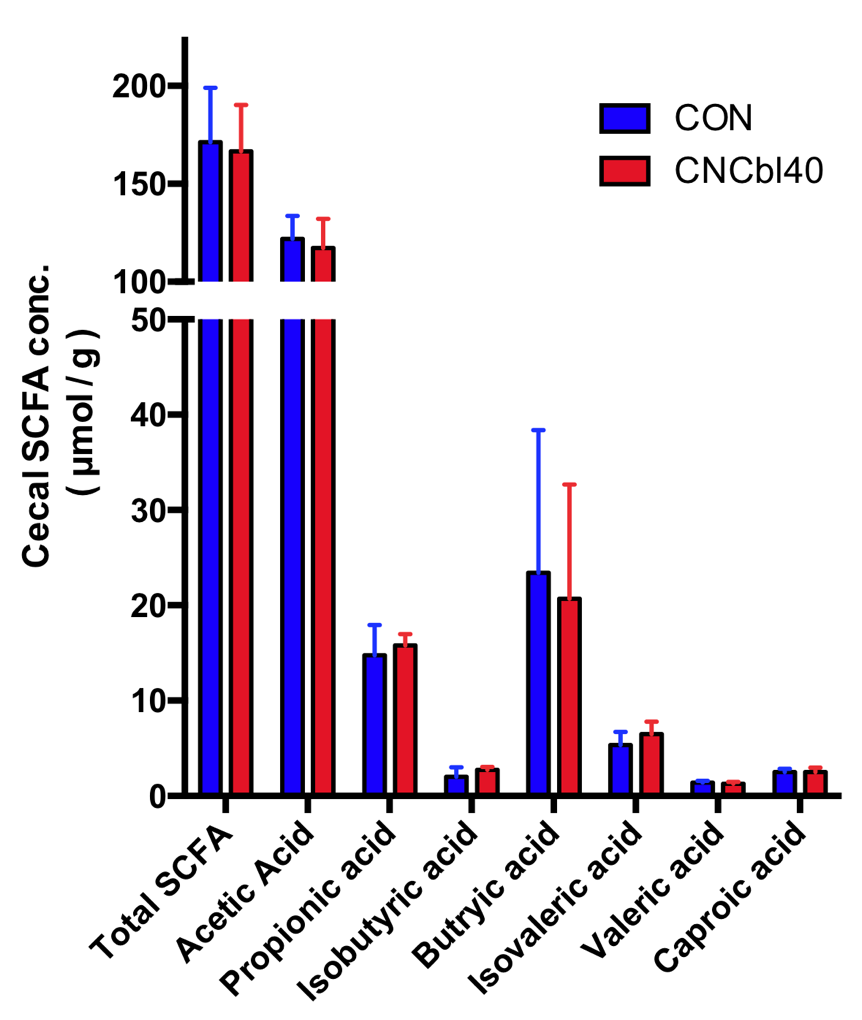


**Figure S2: Cecal SCFA profiles of naïve C3H/HeOuJ mice from the SURV experiment.** Cyanocobalamin supplementation did not alter SCFA production in the cecum (n=6).


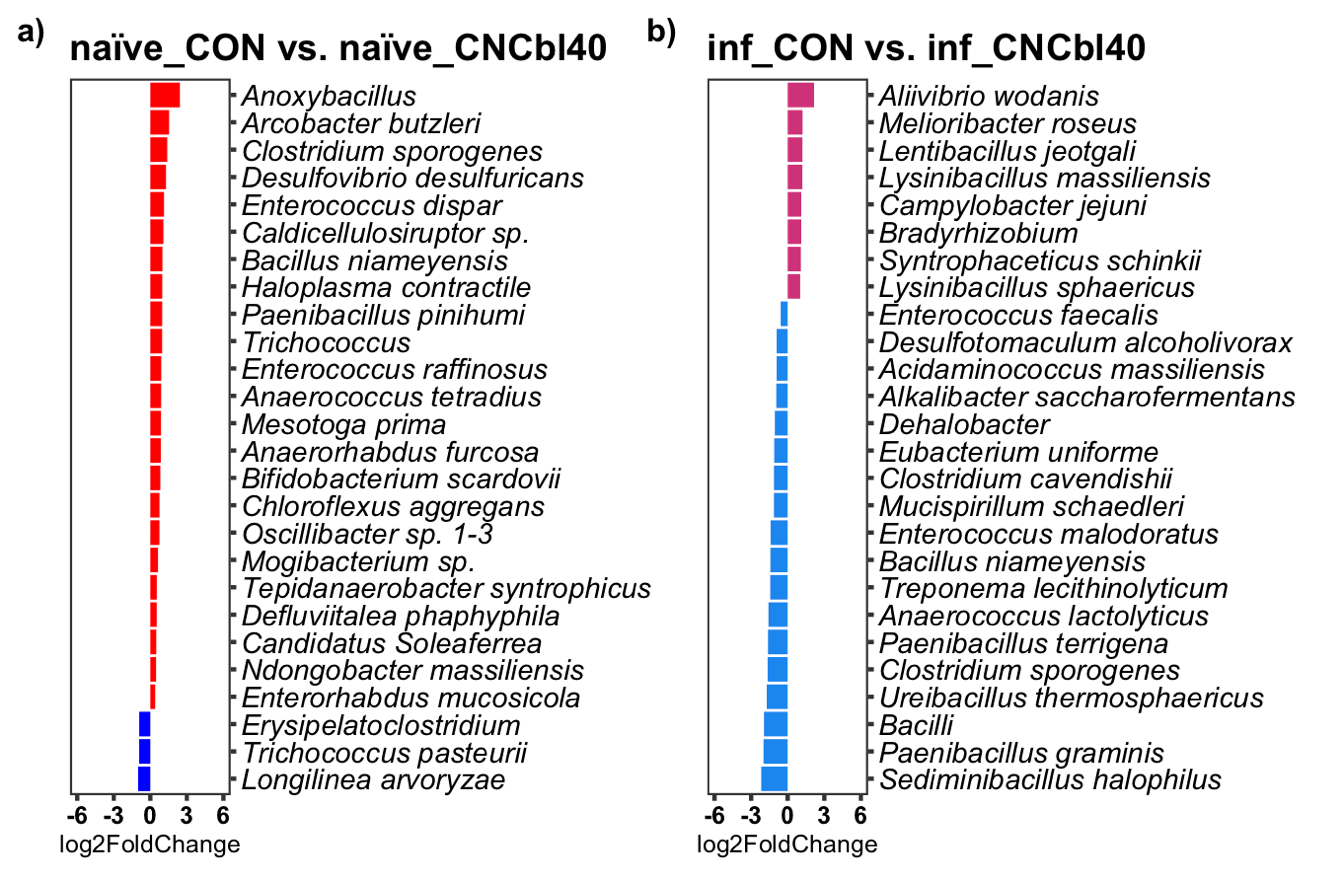


**Figure S3: DESeq2 differential expression analyses of the active microbial community as determine by cecal metatranscriptomics of C3H/HeOuJ mice.** Cyanocobalamin supplementation increased the abundance of numerous active members before infection (a); however, mice at D5PI receiving regular drinking water (b) displayed greater numbers of different active members. All plotted taxa have a non-adjusted *P* value less than 0.05 (n = 8).


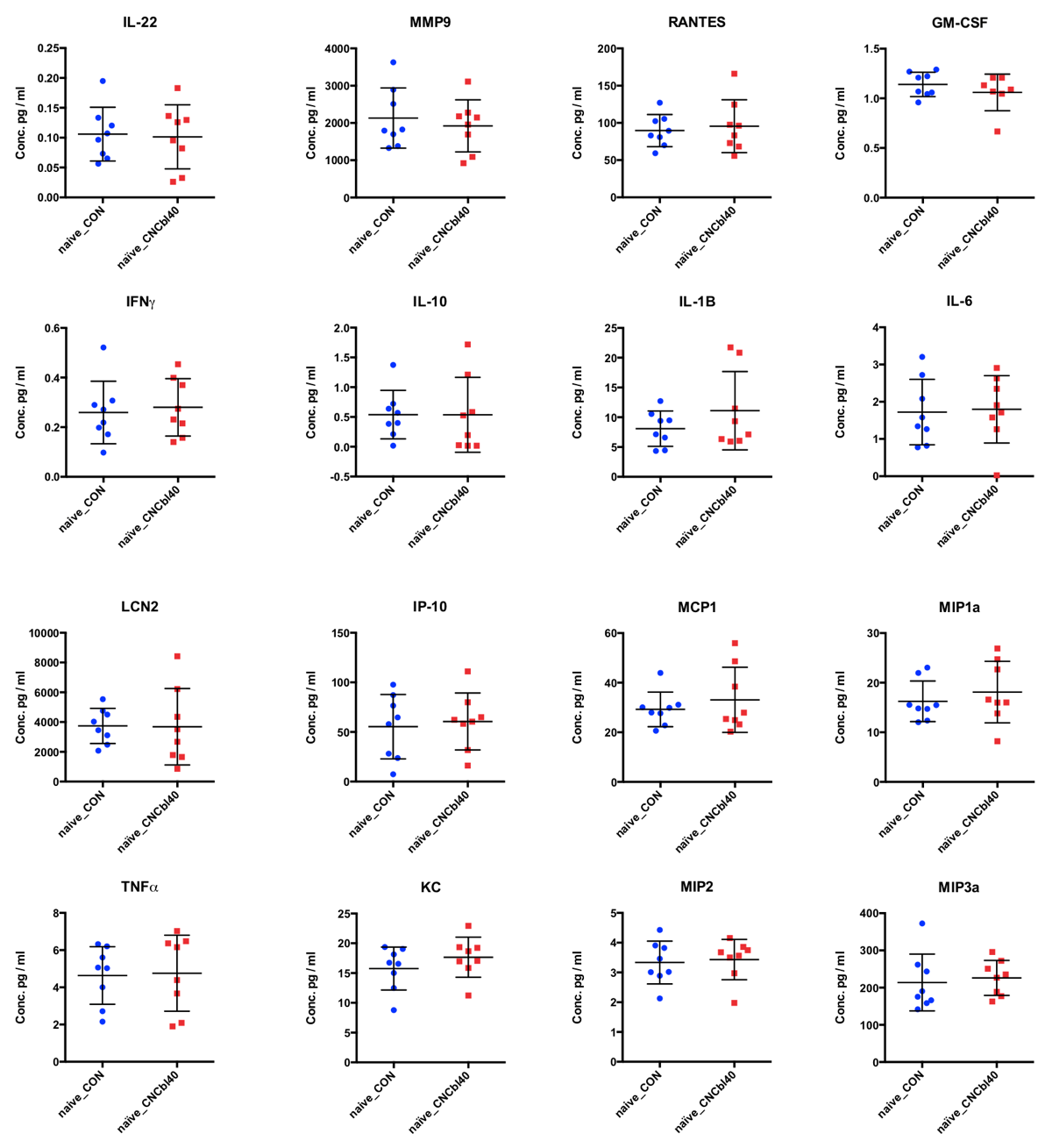


**Figure S4: Inflammation biomarkers in naïve C3H/HeOuJ mice from the EPC experiment** (n = 8).

**
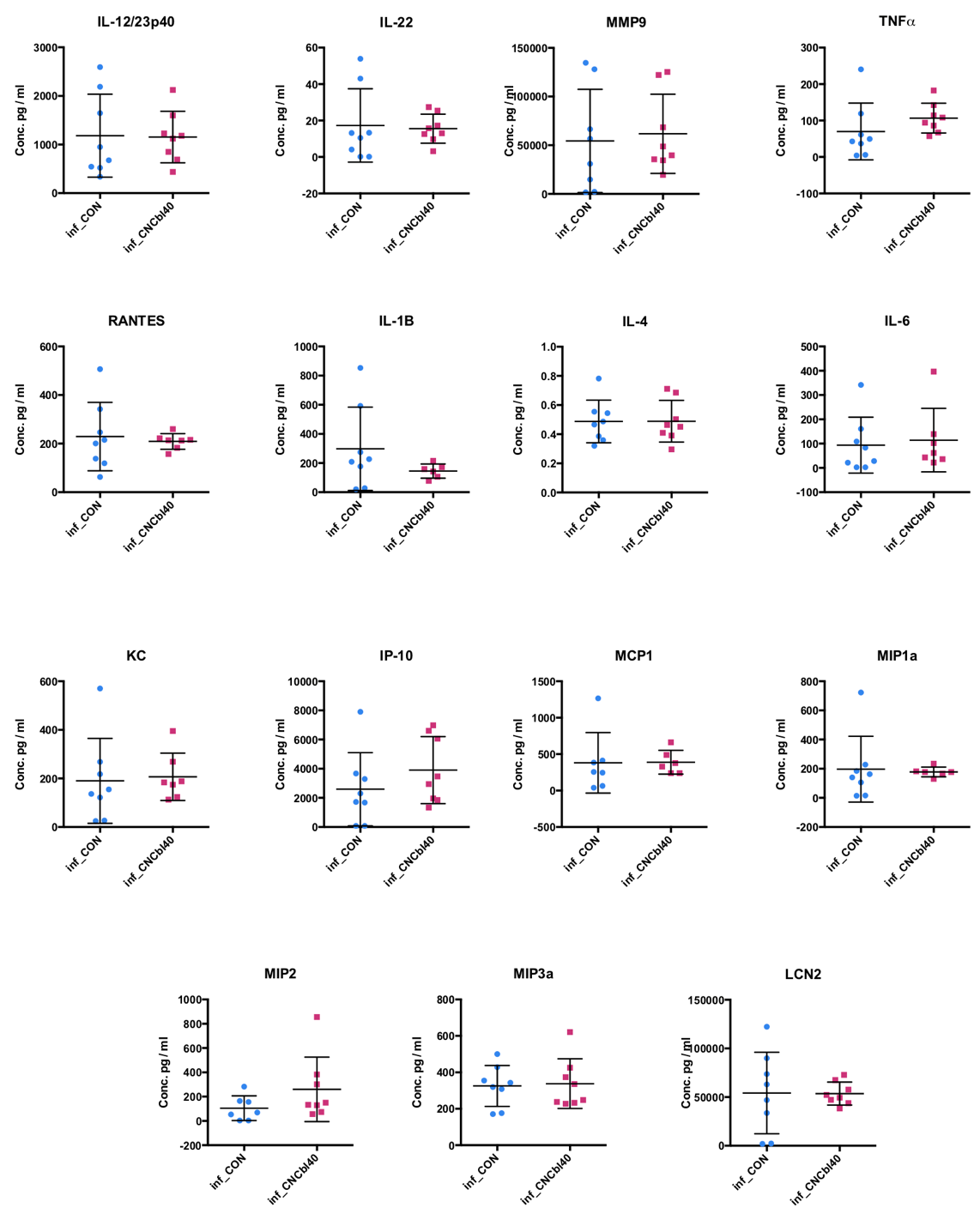
**

**Figure S5: Inflammation biomarkers in *C. rodentium*-challenged C3H/HeOuJ from the EPC experiment** (n = 8).


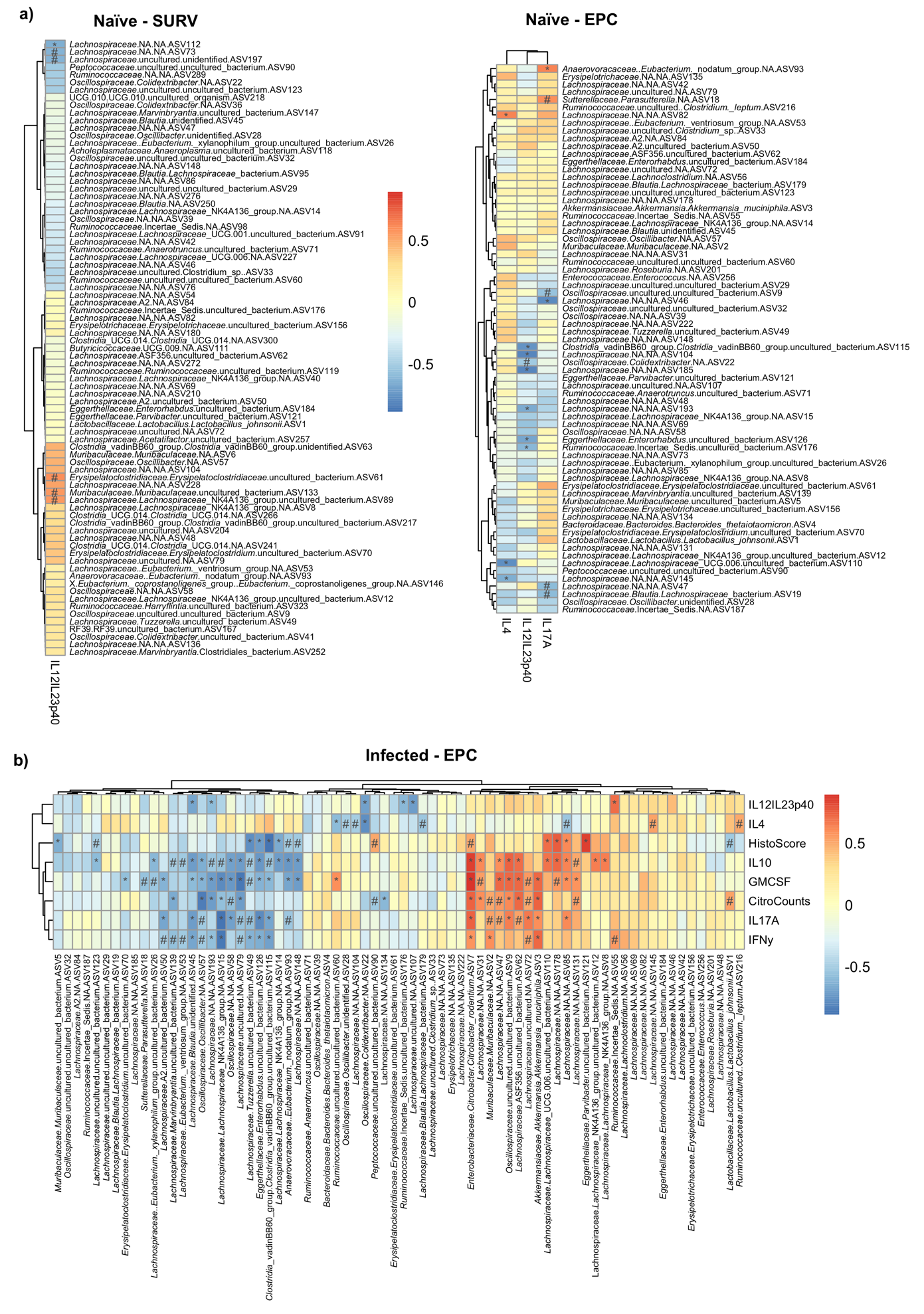


**Figure S6: Spearman’s correlation of the significantly altered immune profiles of colon tissues and microbiota in C3H/HeOuJ mice.** (a) Naïve mice from the SURV (n=10) and EPC experiment (n=11) (b) Infected mice from the EPC experiment (n=12). A trend (*P* < 0.10) is denoted with # and significance (*P* < 0.05) with *.

**Table S1: Real-time qRT-PCR primer list.**

| Target Genes | Oligonucleotide sequences  (5' - 3') | | Product  length | Ref. |
| --- | --- | --- | --- | --- |
| ActB * | F: | TGACAGGATGCAGAAGGAGA | 131 | [26] |
|  | R: | GCTGGAAGGTGGACAGTGAG |  |  |
| IL12A | F: | CCACCCTTGCCCTCCTAAAC | 132 | [26] |
|  | R: | GTTTTTCTCTGGCCGTCTTCA |  |  |
| IL12B | F: | GGGACATCATCAAACCAGACCC | 239 | [26] |
|  | R: | GCCTTTGCATTGGACTTCGG |  |  |
| Rpoa # | F: | ACGTCAGCCGGAAGTGAAAGAAGA | 86 | [24] |
|  | R: | AGCGGACAGTCAATTCCAGATCGT |  |  |
| Ler | F: | ACAGTTTGAATCTCCTGCTCACGC | 98 | [24] |
|  | R: | AATTCGCCCACAACAAGCCCATAC |  |  |
| Tir | F: | ATCAGATATCTCGCAAGCTCG | 134 | [27] |
|  | R: | CAACTCCATCTCCCATTCCTG |  |  |
| EspA | F: | ACGAGGTAACAACCATGCGAGTGT | 87 | [27] |
|  | R: | CTGCCTGGCATTGCTTTCCAGAAT |  |  |

Note: The annealing temperature used for all genes was 60°C. Housekeeping genes used for mice (*) and bacterial (#) gene expression calculations.

**Table S2. Summary of beta-diversity analyses of microbial communities.**

|  |  | Weighted UniFrac | | Unweighted UniFrac | |  |
| --- | --- | --- | --- | --- | --- | --- |
| Region | Comparisons | Dispersion | Orientation | Dispersion | Orientation | |
| Ileum | naïve_CON vs naïve_CNCbl40 | 0.98 | 1 | 1 | 1 | |
|  | inf_CON vs inf_CNCbl40 | 1 | 1 | 1 | 1 | |
|  | naïve_CON vs inf_CON | 1 | 1 | 1 | 1 | |
|  | naïve_CNCbl40 vs inf_CNCbl40 | 1 | 0.49 | 0.88 | 1 | |
|  | naïve_CON vs inf_CNCbl40 | 1 | 1 | 0.97 | 0.4 | |
|  | naïve_CNCbl40 vs inf_CON | 1 | 1 | 0.89 | 0.53 | |
| Cecum | naïve_CON vs naïve_CNCbl40 | 1 | 1 | 0.13 | **0.02** | |
|  | inf_CON vs inf_CNCbl40 | 0.84 | 1 | 1 | 0.25 | |
|  | naïve_CON vs inf_CON | 1 | 0.15 | 0.48 | **0.01** | |
|  | naïve_CNCbl40 vs inf_CNCbl40 | 0.9 | **0.01** | 0.83 | 0.1 | |
|  | naïve_CON vs inf_CNCbl40 | 0.79 | **0.01** | 0.54 | **0.01** | |
|  | naïve_CNCbl40 vs inf_CON | 1 | 0.35 | 0.84 | **0.01** | |
| Colon | naïve_CON vs naïve_CNCbl40 | 0.92 | 1 | 0.25 | **0.01** | |
|  | inf_CON vs inf_CNCbl40 | 1 | 0.17 | 0.96 | 1 | |
|  | naïve_CON vs inf_CON | 0.95 | 1 | 0.33 | 0.81 | |
|  | naïve_CNCbl40 vs inf_CNCbl40 | 0.74 | **0.01** | 0.91 | **0.04** | |
|  | naïve_CON vs inf_CNCbl40 | 0.96 | **0.01** | 0.1 | **0.01** | |
|  | naïve_CNCbl40 vs inf_CON | 0.73 | 0.34 | 1 | **0.05** | |
| Colon  [SURV vs EPC] | CON vs CNCbl40 | 0.21 | 0.7 | **0.01** | **0.01** | |
|  | naïve_CON vs naïve_CNCbl40 | 0.88 | 1 | **0.02** | **0.01** | |
|  | naïve_CON vs CON | 0.31 | **0.01** | 0.76 | **0.01** | |
|  | naïve_CNCbl40 vs CNCbl40 | 0.73 | **0.01** | 0.44 | **0.01** | |
| Cecum  [RNA-seq] | naïve_CON vs naïve_CNCbl40 | 0.38 | 1 | 0.29 | 0.6 | |
|  | inf_CON vs inf_CNCbl40 | 0.71 | 0.85 | 0.53 | 0.32 | |
|  | naïve_CON vs inf_CON | 0.06 | 0.77 | 0.16 | **0.01** | |
|  | naïve_CNCbl40 vs inf_CNCbl40 | 1 | 0.85 | 0.73 | **0.042** | |
|  | naïve_CON vs inf_CNCbl40 | 0.4 | 0.08 | 0.87 | **0.01** | |
|  | naïve_CNCbl40 vs inf_CON | 0.74 | 1 | 1 | 0.08 | |

Note: Dispersion p-values were calculated with betadisper() using a permuted model that indicates a difference in dispersion between groups. Orientation p-values were calculated with pairwise.adonis() and adjusted using bonferroni (perm=999) method, and indicates significantly different clustering between groups.
