# Supplementary material for: Over supplementation with vitamin B12 alters microbe-host interactions in the gut leading to accelerated Citrobacter rodentium colonization and pathogenesis in mice
Source: Microbiome. 2023 Feb 3;11:21. doi: 10.1186/s40168-023-01461-w (PMC9896722; doi:10.1186/s40168-023-01461-w)
Supplement: Supplementary file 3 — Additional file 2. Microbiota R analysis. [file 40168_2023_1461_MOESM2_ESM.docx]

**Over supplementation with vitamin B12 alters microbe-host interactions in the gut leading to accelerated *Citrobacter rodentium* colonization and pathogenesis in mice**

R script analyses:


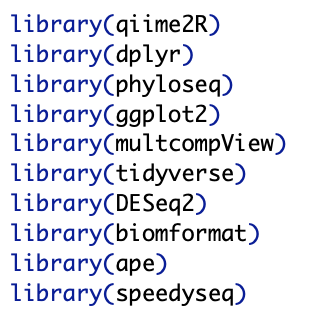


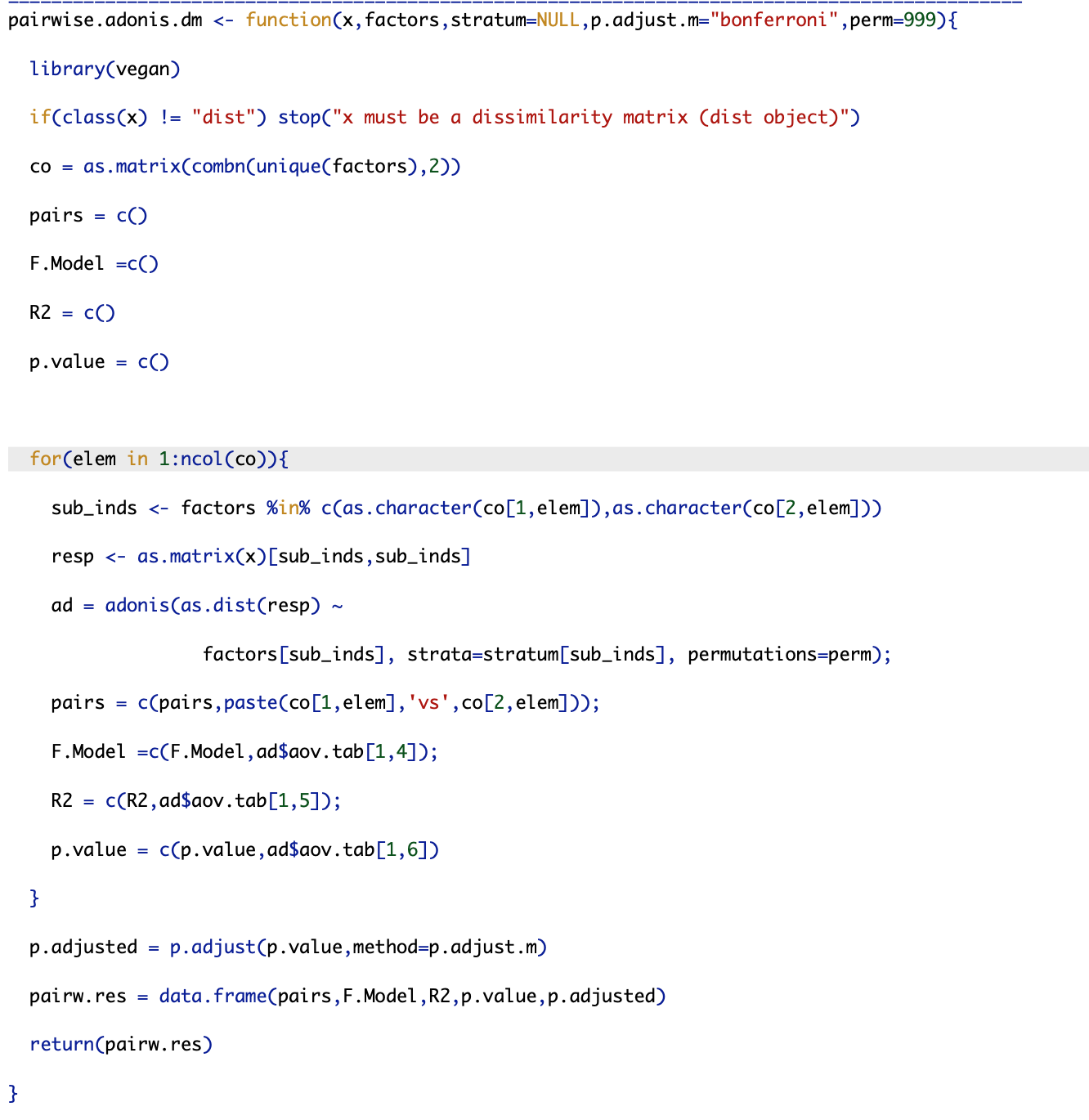


**Initial filtering:**

physeq.rm <- filter_taxa(physeq, function(x){sum(x > 0) > 1}, prune = T)

physeq.clean <- subset_taxa(physeq.rm, (Genus!="Chloroplast") | is.na(Genus))

physeq.clean <- subset_taxa(physeq.clean, (Genus!="Mitochondria") | is.na(Genus))

physeq.clean <- subset_taxa(physeq.clean, (Phylum!="NA"))

**Subset, additional filtering, plotting and analyses:**

**ILEUM (EPC):**

ileum <- subset_samples(physeq.clean, Section=="Ileum")

ileum.filtered <- filter_taxa(ileum.rm, function(x) sum(x > 3) > (0.2*length(x)), TRUE )

**Beta Diversity**

min(sample_sums(ileum.filtered)) #### min = 21497

ileum.rarefied <- rarefy_even_depth(ileum.filtered, sample.size = min(sample_sums(ileum.filtered)), rngseed=1)

**Unweighted UniFrac:**

ileum1 <- ordinate(ileum.rarefied, "PCoA", "unifrac", weighted=F)

ileum1 <- plot_ordination(ileum.rarefied, ileum1, color = "Treatment", axes=c(1,2), shape= "Treatment") + scale_colour_manual(name = "Treatment",values = c("blue", "red", "dodgerblue2", "violetred3"), limits = c("Control","CNCbl","Control_inf","CNCbl_inf")) + scale_shape_manual(name = "Treatment",values = c(16,16,8,8), limits = c("Control","CNCbl","Control_inf","CNCbl_inf"))+ stat_ellipse(type = "t", linetype = 1, size = .5, level =.95) + ggtitle("Ileum - Unweighted UniFrac") + geom_point(size=4) + theme_bw() + theme(panel.grid.major = element_blank(), panel.grid.minor = element_blank()) + theme(plot.title = element_text(size=18, face= "bold", hjust=0.5)) + theme(text = element_text(size = 14, face= "bold")) + guides(fill = guide_legend(override.aes = list(linetype = 0)), color = guide_legend(override.aes = list(linetype = 0))) + theme(legend.title=element_blank())

ileum1 + xlab("PC1 [31.4%]") + ylab("PC2 [16.8%]")

ileum1a = phyloseq::distance(ileum.rarefied, "unifrac", weighted=F)

pairwise.adonis.dm(ileum1a, phyloseq::sample_data(ileum.rarefied)$Treatment)


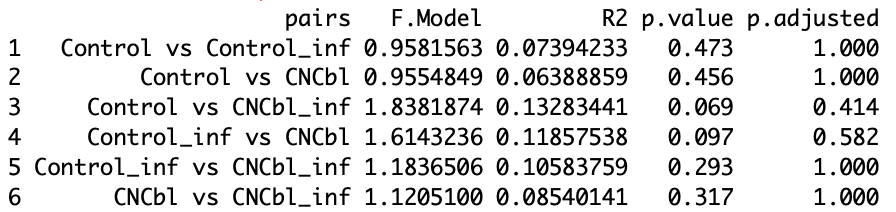


ileum1aa <- betadisper(ileum1a, phyloseq::sample_data(ileum.rarefied)$Treatment)

permutest(ileum1aa, pairwise=T , permutations = 999)

TukeyHSD(ileum1aa)


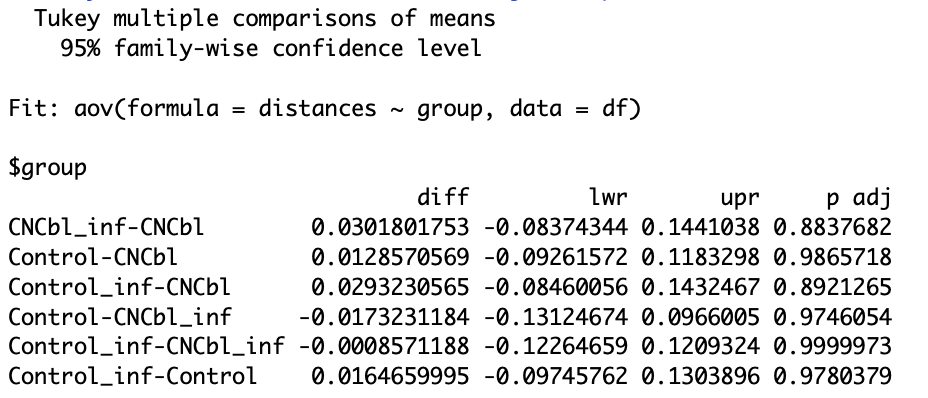


**Weighted UniFrac:**

ileum2 <- ordinate(ileum.rarefied, "PCoA", "unifrac", weighted=T)

ileum2 <- plot_ordination(ileum.rarefied, ileum2, color = "Treatment", axes=c(1,2), shape= "Treatment") + scale_colour_manual(name = "Treatment",values = c("blue", "red", "dodgerblue2", "violetred3"), limits = c("Control","CNCbl","Control_inf","CNCbl_inf")) + scale_shape_manual(name = "Treatment",values = c(16,16,8,8), limits = c("Control","CNCbl","Control_inf","CNCbl_inf"))+ stat_ellipse(type = "t", linetype = 1, size = .5, level =.95) + ggtitle("Ileum - Weighted UniFrac") + geom_point(size=4) + theme_bw() + theme(panel.grid.major = element_blank(), panel.grid.minor = element_blank()) + theme(plot.title = element_text(size=18, face= "bold", hjust=0.5)) + theme(text = element_text(size = 14, face= "bold")) + guides(fill = guide_legend(override.aes = list(linetype = 0)), color = guide_legend(override.aes = list(linetype = 0))) + theme(legend.title=element_blank())

ileum2 + xlab("PC1 [61.9%]") + ylab("PC2 [18.8%]")

ileum2a = phyloseq::distance(ileum.rarefied, "unifrac", weighted=T)

pairwise.adonis.dm(ileum2a, phyloseq::sample_data(ileum.rarefied)$Treatment)


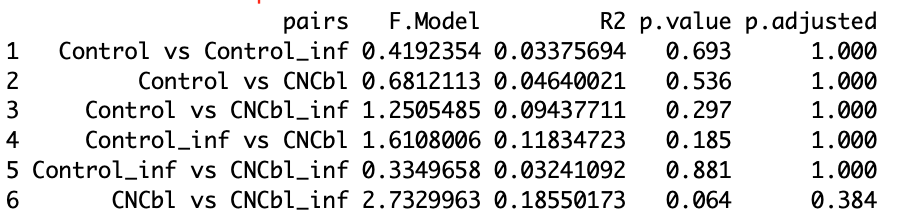


ileum2aa <- betadisper(ileum2a, phyloseq::sample_data(ileum.rarefied)$Treatment)

permutest(ileum2aa, pairwise=T , permutations = 999)

TukeyHSD(ileum2aa)


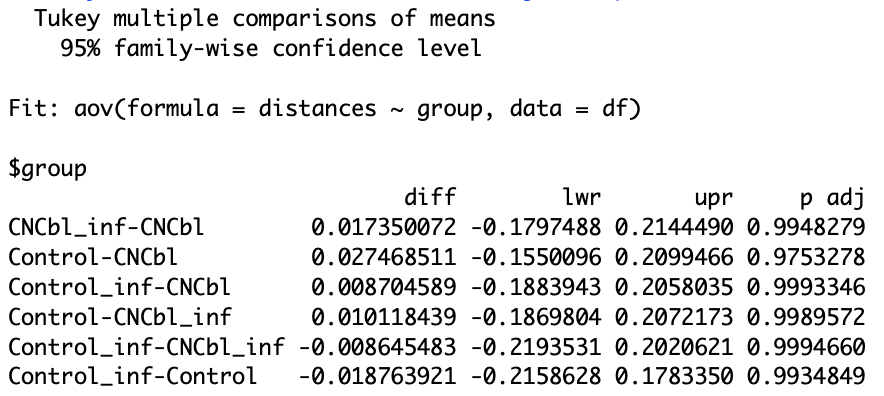


**Alpha Diversity:**

ileum.adiv$Treatment = factor(sample_data(ileum.rarefied)$Treatment, levels = c("Control","CNCbl","Control_inf","CNCbl_inf"))

levels(ileum.adiv$Treatment)

ileum.adiv %>%

gather(key = metric, value = value, c("Observed", "Shannon", "PD")) %>%

mutate(metric = factor(metric, levels = c("Observed", "Shannon", "PD"))) %>%

ggplot(aes(x = Treatment, y = value, colour = Treatment)) + geom_boxplot(outlier.color = NA) + scale_colour_manual(name = "Treatment",values = c("blue", "red", "dodgerblue2", "violetred3"), limits = c("Control","CNCbl","Control_inf","CNCbl_inf")) + scale_shape_manual(name = "Treatment", values = c(16,16,8,8), limits = c("Control","CNCbl","Control_inf","CNCbl_inf")) + geom_jitter(aes(color = Treatment, shape = Treatment), height = 0, width = .2, size = 3) + labs(x = "", y = "") + facet_wrap(~metric, scales = "free_y") + theme_bw() + theme(panel.grid.major = element_blank(), panel.grid.minor = element_blank()) + theme(strip.text.x = element_text(size=16, face="bold")) + theme(axis.text.x = element_blank(), axis.text.y = element_text(face="bold", size=12, color = "black")) + theme(legend.position="none") + scale_y_continuous(expand = expansion(mult = c(0.1,0.3)))

ANOVA = aov(ileum.adiv$Observed ~ ileum.adiv$Treatment)

TUKEY <- TukeyHSD(ANOVA, 'ileum.adiv$Treatment', conf.level=0.95)

TUKEY


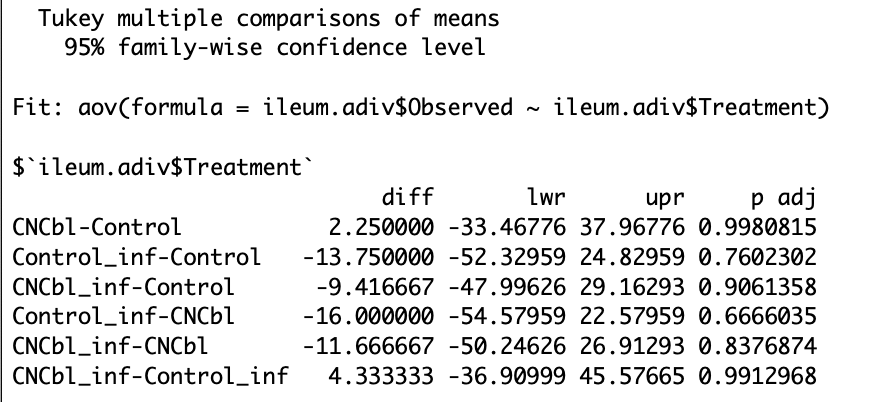


ANOVA = aov(ileum.adiv$Shannon ~ ileum.adiv$Treatment)

TUKEY <- TukeyHSD(ANOVA, 'ileum.adiv$Treatment', conf.level=0.95)

TUKEY


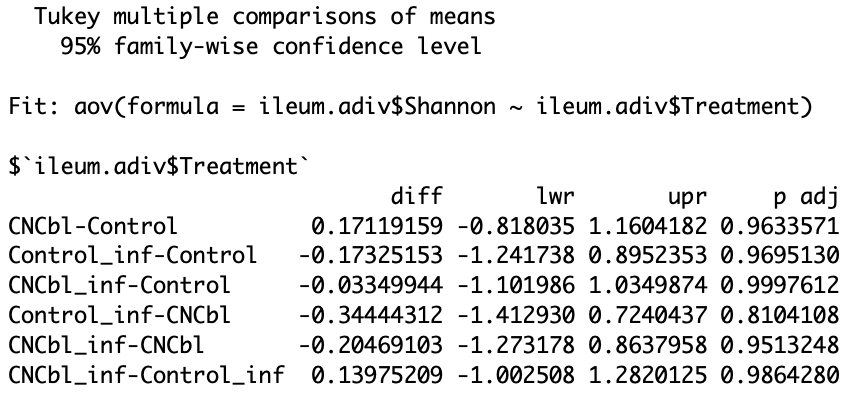


ANOVA = aov(ileum.adiv$PD ~ ileum.adiv$Treatment)

TUKEY <- TukeyHSD(ANOVA, 'ileum.adiv$Treatment', conf.level=0.95)

TUKEY


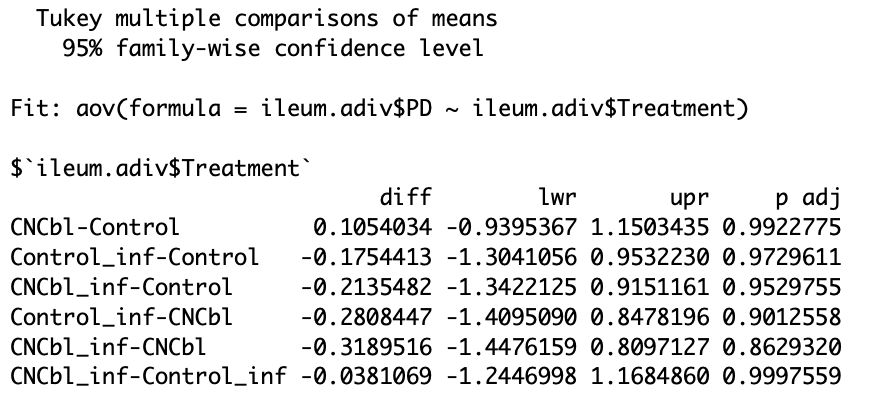


**Deseq2 Analysis: See ‘Microbiota Deseq2 Results Table’ for results**

alpha = 0.05 ### significance cutoff

####### Naïve ########

ileum.naïve <- subset_samples(ileum.filtered, Infection=="N")

ileum.naïve <- tree_glom(ileum.naïve, 0.05)

ileum.naïve

sample_data(ileum.naïve)$Treatment <- factor(sample_data(ileum.naïve)$Treatment, levels = c("Control","CNCbl"))

ds1a = phyloseq_to_deseq2(ileum.naïve, ~Treatment)

ds1a = DESeq(ds1a)

res1 = results(ds1a, contrast=c("Treatment", "CNCbl", "Control"))

res1 = res1[order(res1$pvalue, na.last=NA), ]

res1

res1 = cbind(as(res1, "data.frame"), as(tax_table(ileum.naïve)[rownames(res1), ], "matrix"))

res_sig1 = res1[(res1$pvalue < alpha), ]

res_sig1

df1 <- data.matrix(row.names(res_sig1))

colnames(df1) <- c("ASV")

fix(df1)

res_sig1 <- cbind(res_sig1, df1)

res_sig1$ID = paste(res_sig1$ASV, res_sig1$Phylum, res_sig1$Order, res_sig1$Family, res_sig1$Genus, res_sig1$Species, sep=" ")

fix(res_sig1)

ggplot(data = res_sig1, aes(y= reorder(ID, log2FoldChange), x=log2FoldChange, fill = log2FoldChange < 0)) + geom_bar(stat="identity") + scale_fill_manual(values = c("red","blue")) + theme_bw() + theme(panel.grid.major = element_blank(), panel.grid.minor = element_blank()) + labs(y = "") + theme(legend.position="none") + theme(axis.text.x = element_text(face="bold", size=12, color = "black"), axis.text.y = element_text(size=14, color = "black"), axis.title.x = element_text(size=12, color = "black"), plot.title = element_text(size = 18, face="bold")) + ggtitle("Ileum - Control vs. CNCbl") + scale_x_continuous(limits = c(-10,10)) + scale_y_discrete(position = "right")

####### Infected #########

ileum.infected <- subset_samples(ileum.filtered, Infection=="Y")

ileum.glom <- tree_glom(ileum.infected, 0.05)

ileum.glom

sample_data(ileum.glom)$Treatment <- factor(sample_data(ileum.glom)$Treatment, levels = c("Control_inf","CNCbl_inf"))

ds1b = phyloseq_to_deseq2(ileum.glom, ~Treatment)

ds1b = DESeq(ds1b)

res2 = results(ds1b, contrast=c("Treatment", "CNCbl_inf", "Control_inf"))

res2 = res2[order(res2$pvalue, na.last=NA), ]

res2

res2 = cbind(as(res2, "data.frame"), as(tax_table(ileum.filtered)[rownames(res2), ], "matrix"))

res_sig2 = res2[(res2$pvalue < alpha), ]

res_sig2

df2 <- data.matrix(row.names(res_sig2))

colnames(df2) <- c("ASV")

fix(df2)

res_sig2 <- cbind(res_sig2, df2)

res_sig2$ID = paste(res_sig2$ASV, res_sig2$Phylum, res_sig2$Order, res_sig2$Family, res_sig2$Genus, res_sig2$Species, sep=" ")

fix(res_sig2)

ggplot(data = res_sig2, aes(y= reorder(ID, log2FoldChange), x=log2FoldChange, fill = log2FoldChange < 0)) + geom_bar(stat="identity") + scale_fill_manual(values = c("violetred3", "dodgerblue2")) + theme_bw() + theme(panel.grid.major = element_blank(), panel.grid.minor = element_blank()) + labs(y = "") + theme(legend.position="none") + theme(axis.text.x = element_text(face="bold", size=12, color = "black"), axis.text.y = element_text(size=14, color = "black"), axis.title.x = element_text(size=12, color = "black"), plot.title = element_text(size = 18, face="bold")) + ggtitle("Ileum - Control_inf vs. CNCbl_inf") + scale_x_continuous(limits = c(-10,10)) + scale_y_discrete(position = "right")

**CECUM (EPC):**

cecum <- subset_samples(physeq.clean, Section=="Cecum")

cecum.filtered <- filter_taxa(cecum.rm, function(x) sum(x > 3) > (0.2*length(x)), TRUE )

**Beta Diversity**

sort(sample_sums(cecum.filtered)) ##min = 34012

cecum.rarefied <- rarefy_even_depth(cecum.filtered, sample.size = min(sample_sums(cecum.filtered)), rngseed=1)

**Unweighted UniFrac:**

cecum1 <- ordinate(cecum.rarefied, "PCoA", "unifrac", weighted=F)

cecum1 <- plot_ordination(cecum.rarefied, cecum1, color = "Treatment", axes=c(1,2), shape= "Treatment") + scale_colour_manual(name = "Treatment",values = c("blue", "red", "dodgerblue2", "violetred3"), limits = c("Control","CNCbl","Control_inf","CNCbl_inf")) + scale_shape_manual(name = "Treatment",values = c(16,16,8,8), limits = c("Control","CNCbl","Control_inf","CNCbl_inf"))+ stat_ellipse(type = "t", linetype = 1, size = .5, level =.95) + ggtitle("Cecum - Unweighted UniFrac") + geom_point(size=4) + theme_bw() + theme(panel.grid.major = element_blank(), panel.grid.minor = element_blank()) + theme(plot.title = element_text(size=18, face= "bold", hjust=0.5)) + theme(text = element_text(size = 14, face= "bold")) + guides(fill = guide_legend(override.aes = list(linetype = 0)), color = guide_legend(override.aes = list(linetype = 0))) + theme(legend.title=element_blank())

cecum1 + xlab("PC1 [32.5%]") + ylab("PC2 [19.4%]")

cecum1a = phyloseq::distance(cecum.rarefied, "unifrac", weighted=F)

pairwise.adonis.dm(cecum1a, phyloseq::sample_data(cecum.rarefied)$Treatment)


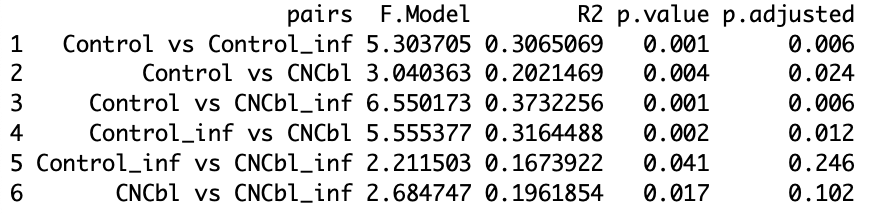


cecum1aa <- betadisper(cecum1a, phyloseq::sample_data(cecum.rarefied)$Treatment)

permutest(cecum1aa, pairwise=T , permutations = 999)

TukeyHSD(cecum1aa)


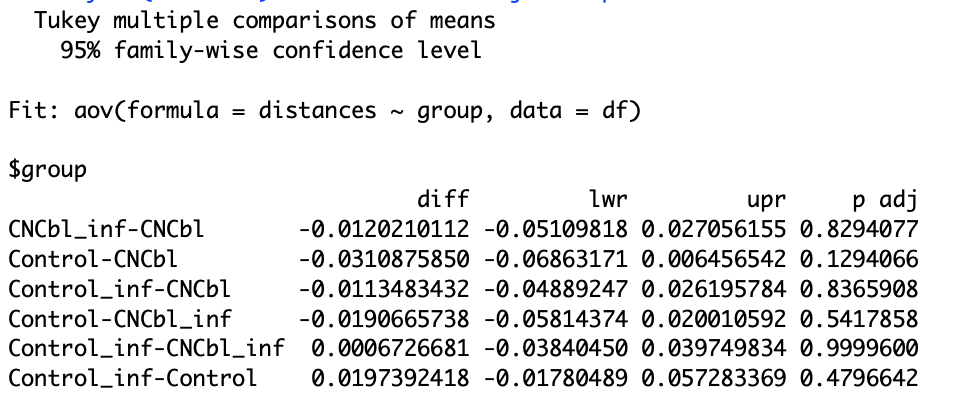


**Weighted UniFrac:**

cecum2 <- ordinate(cecum.rarefied, "PCoA", "unifrac", weighted=T)

cecum2 <- plot_ordination(cecum.rarefied, cecum2, color = "Treatment", axes=c(1,2), shape= "Treatment") + scale_colour_manual(name = "Treatment",values = c("blue", "red", "dodgerblue2", "violetred3"), limits = c("Control","CNCbl","Control_inf","CNCbl_inf")) + scale_shape_manual(name = "Treatment",values = c(16,16,8,8), limits = c("Control","CNCbl","Control_inf","CNCbl_inf"))+ stat_ellipse(type = "t", linetype = 1, size = .5, level =.95) + ggtitle("Cecum - Weighted UniFrac") + geom_point(size=4) + theme_bw() + theme(panel.grid.major = element_blank(), panel.grid.minor = element_blank()) + theme(plot.title = element_text(size=18, face= "bold", hjust=0.5)) + theme(text = element_text(size = 14, face= "bold")) + guides(fill = guide_legend(override.aes = list(linetype = 0)), color = guide_legend(override.aes = list(linetype = 0))) + theme(legend.title=element_blank())

cecum2 + xlab("PC1 [42.9%]") + ylab("PC2 [24.8%]")

cecum2a = phyloseq::distance(cecum.rarefied, "unifrac", weighted=T)

pairwise.adonis.dm(cecum2a, phyloseq::sample_data(cecum.rarefied)$Treatment)

**
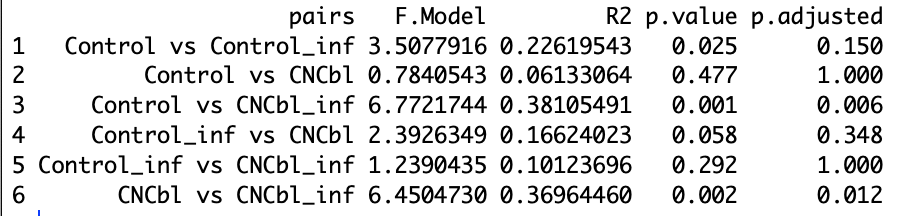
**

cecum2aa <- betadisper(cecum2a, phyloseq::sample_data(cecum.rarefied)$Treatment)

permutest(cecum2aa, pairwise=T , permutations = 999)

TukeyHSD(cecum2aa)

**
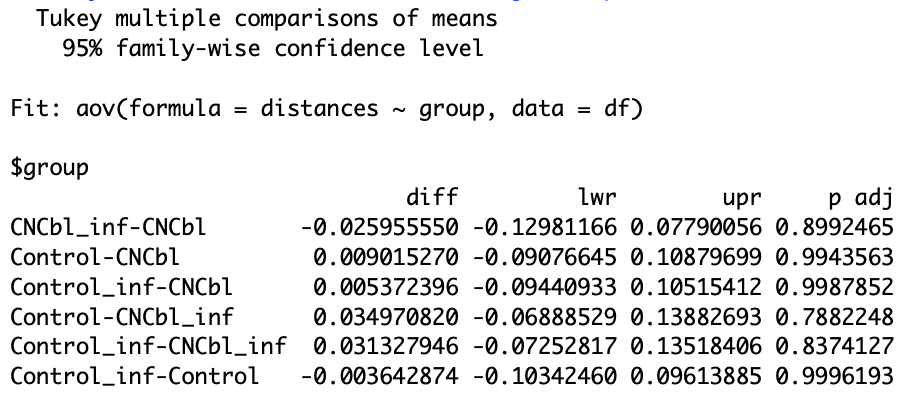
**

**Alpha Diversity:**

estimate_richness(cecum.rarefied)

cecum.adiv <- data.frame( "Chao1" = phyloseq::estimate_richness(cecum.rarefied, measures = "Chao1"), "Shannon" = phyloseq::estimate_richness(cecum.rarefied, measures = "Shannon"), "Obversed" = phyloseq::estimate_richness(cecum.rarefied, measures = "Observed"), "PD" = picante::pd(samp = data.frame(t(data.frame(phyloseq::otu_table(cecum.rarefied)))), tree = phyloseq::phy_tree(cecum.rarefied))[,1], "Treatment" = phyloseq::sample_data(cecum.rarefied)$Treatment)

cecum.adiv$Treatment = factor(sample_data(cecum.rarefied)$Treatment, levels = c("Control","CNCbl","Control_inf","CNCbl_inf"))

levels(cecum.adiv$Treatment)

cecum.adiv %>%

gather(key = metric, value = value, c("Observed", "Shannon", "PD")) %>%

mutate(metric = factor(metric, levels = c("Observed", "Shannon", "PD"))) %>%

ggplot(aes(x = Treatment, y = value, colour = Treatment)) + geom_boxplot(outlier.color = NA) + scale_colour_manual(name = "Treatment",values = c("blue", "red", "dodgerblue2", "violetred3"), limits = c("Control","CNCbl","Control_inf","CNCbl_inf")) + scale_shape_manual(name = "Treatment", values = c(16,16,8,8), limits = c("Control","CNCbl","Control_inf","CNCbl_inf")) + geom_jitter(aes(color = Treatment, shape = Treatment), height = 0, width = .2, size = 3) + labs(x = "", y = "") + facet_wrap(~metric, scales = "free_y") + theme_bw() + theme(panel.grid.major = element_blank(), panel.grid.minor = element_blank()) + theme(strip.text.x = element_text(size=16, face="bold")) + theme(axis.text.x = element_blank(), axis.text.y = element_text(face="bold", size=12, color = "black")) + theme(legend.position="none") + scale_y_continuous(expand = expansion(mult = c(0.1,0.3)))

ANOVA = aov(cecum.adiv$Observed ~ cecum.adiv$Treatment)

TUKEY <- TukeyHSD(ANOVA, 'cecum.adiv$Treatment', conf.level=0.95)

TUKEY

**
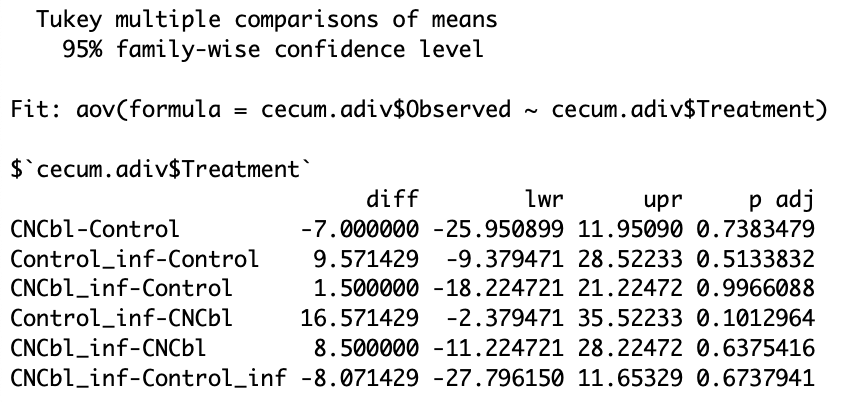
**

ANOVA = aov(cecum.adiv$Shannon ~ cecum.adiv$Treatment)

TUKEY <- TukeyHSD(ANOVA, 'cecum.adiv$Treatment', conf.level=0.95)

TUKEY

**
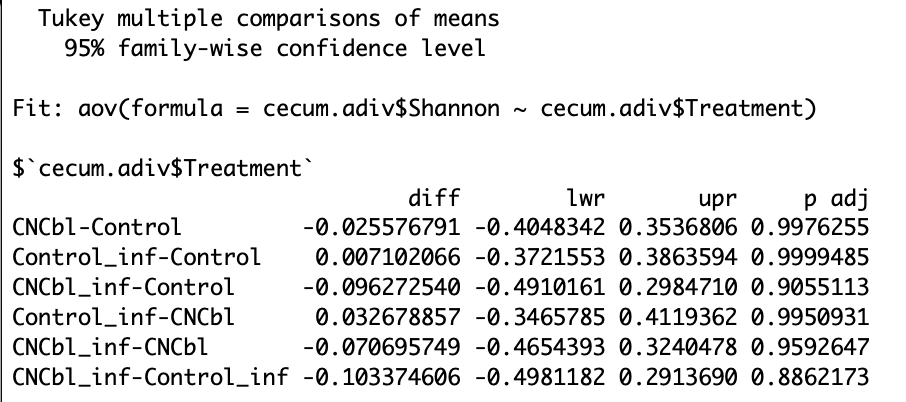
**

ANOVA = aov(cecum.adiv$PD ~ cecum.adiv$Treatment)

TUKEY <- TukeyHSD(ANOVA, 'cecum.adiv$Treatment', conf.level=0.95)

TUKEY

**
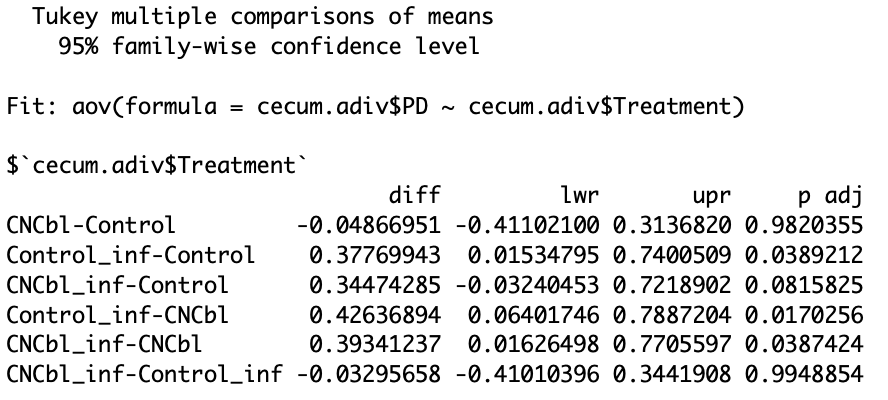
**

**Deseq2 Analysis: See ‘Microbiota Deseq2 Results Tables’ for results**

alpha = 0.05 ### significance cutoff

####### Naïve ########

cecum.naïve <- subset_samples(cecum.filtered, Infection=="N")

cecum.naïve <- tree_glom(cecum.naïve, 0.05)

cecum.naïve

sample_data(cecum.naïve)$Treatment <- factor(sample_data(cecum.naïve)$Treatment, levels = c("Control","CNCbl"))

ds2a = phyloseq_to_deseq2(cecum.naïve, ~Treatment)

ds2a = DESeq(ds2a)

res3 = results(ds2a, contrast=c("Treatment", "CNCbl", "Control"), alpha=alpha)

res3 = res3[order(res3$pvalue, na.last=NA), ]

res3

res3 = cbind(as(res3, "data.frame"), as(tax_table(cecum.naïve)[rownames(res3), ], "matrix"))

res_sig3 = res3[(res3$pvalue < alpha), ]

res_sig3

df1 <- data.matrix(row.names(res_sig3))

colnames(df1) <- c("ASV")

fix(df1)

res_sig3 <- cbind(res_sig3, df1)

res_sig3$ID = paste(res_sig3$ASV, res_sig3$Phylum, res_sig3$Family, res_sig3$Genus, res_sig3$Species, sep="|")

fix(res_sig3)

ggplot(data = res_sig3, aes(y= reorder(ID, log2FoldChange), x=log2FoldChange, fill = log2FoldChange < 0)) + geom_bar(stat="identity") + scale_fill_manual(values = c("red", "blue")) + theme_bw() + theme(panel.grid.major = element_blank(), panel.grid.minor = element_blank()) + labs(y = "") + theme(legend.position="none") + theme(axis.text.x = element_text(face="bold", size=12, color = "black"), axis.text.y = element_text(size=14 , color = "black"), axis.title.x = element_text(size=12, color = "black"), plot.title = element_text(size = 18, face="bold")) + ggtitle("Cecum - Control vs. CNCbl") + scale_x_continuous(limits = c(-10,10)) + scale_y_discrete(position = "right")

####### Infected #########

cecum.infected <- subset_samples(cecum.filtered, Infection=="Y")

cecum.infected <- tree_glom(cecum.infected, 0.05)

cecum.infected

sample_data(cecum.infected)$Treatment <- factor(sample_data(cecum.infected)$Treatment,levels = c("Control_inf","CNCbl_inf"))

ds2b = phyloseq_to_deseq2(cecum.infected, ~Treatment)

ds2b = DESeq(ds2b)

res4 = results(ds2b, contrast=c("Treatment", "CNCbl_inf", "Control_inf"), alpha=alpha)

res4 = res4[order(res4$pvalue, na.last=NA), ]

res4

res4 = cbind(as(res4, "data.frame"), as(tax_table(cecum.infected)[rownames(res4), ], "matrix"))

res_sig4 = res4[(res4$pvalue < alpha), ]

res_sig4

df2 <- data.matrix(row.names(res_sig4))

colnames(df2) <- c("ASV")

fix(df2)

res_sig4 <- cbind(res_sig4, df2)

res_sig4$ID = paste(res_sig4$ASV, res_sig4$Phylum, res_sig4$Family, res_sig4$Genus, res_sig4$Species, sep="|")

fix(res_sig4)

ggplot(data = res_sig4, aes(y= reorder(ID, log2FoldChange), x=log2FoldChange, fill = log2FoldChange < 0)) + geom_bar(stat="identity") + scale_fill_manual(values = c("violetred3","dodgerblue2")) + theme_bw() + theme(panel.grid.major = element_blank(), panel.grid.minor = element_blank()) + labs(y = "") + theme(legend.position="none") + theme(axis.text.x = element_text(face="bold", size=12, color = "black"), axis.text.y = element_text(size=14 , color = "black"), axis.title.x = element_text(size=12, color = "black"), plot.title = element_text(size = 18, face="bold")) + ggtitle("Cecum - Control_inf vs. CNCbl_inf") + scale_x_continuous(limits = c(-10,10)) + scale_y_discrete(position = "right")

**COLON (EPC):**

colon <- subset_samples(physeq.clean, Section=="Colon")

colon <- subset_samples(colon, Experiment=="EPC")

colon.filtered <- filter_taxa(colon, function(x) sum(x > 3) > (0.2*length(x)), TRUE )

**Beta Diversity**

sort(sample_sums(colon.filtered)) #7435

colon.rarefied <- rarefy_even_depth(colon.filtered, sample.size = min(sample_sums(colon.filtered)), rngseed=1)

**Unweighted UniFrac:**

colon1 <- ordinate(colon.rarefied, "PCoA", "unifrac", weighted=F)

colon1 <- plot_ordination(colon.rarefied, colon1, color = "Treatment", axes=c(1,2), shape= "Treatment") + scale_colour_manual(name = "Treatment",values = c("blue", "red", "dodgerblue2", "violetred3"), limits = c("Control","CNCbl","Control_inf","CNCbl_inf")) + scale_shape_manual(name = "Treatment",values = c(16,16,8,8), limits = c("Control","CNCbl","Control_inf","CNCbl_inf"))+ stat_ellipse(type = "t", linetype = 1, size = .5, level =.95) + ggtitle("Colon - Unweighted UniFrac ") + geom_point(size=4) + theme_bw() + theme(panel.grid.major = element_blank(), panel.grid.minor = element_blank()) + theme(plot.title = element_text(size=18, face= "bold", hjust=0.5)) + theme(text = element_text(size = 14, face= "bold")) + guides(fill = guide_legend(override.aes = list(linetype = 0)), color = guide_legend(override.aes = list(linetype = 0))) + theme(legend.title=element_blank())

colon1 + xlab("PC1 [31.8%]") + ylab("PC2 [12.8%]") + theme(legend.position =c(.85,.85))

colon1a = phyloseq::distance(colon.rarefied, "unifrac", weighted=F)

pairwise.adonis.dm(colon1a, phyloseq::sample_data(colon.rarefied)$Treatment)

**
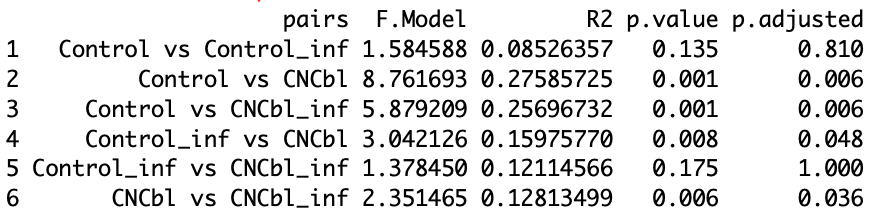
**

colon1aa <- betadisper(colon1a, phyloseq::sample_data(colon.rarefied)$Treatment)

permutest(colon1aa, pairwise=T , permutations = 999)

TukeyHSD(colon1aa)

**
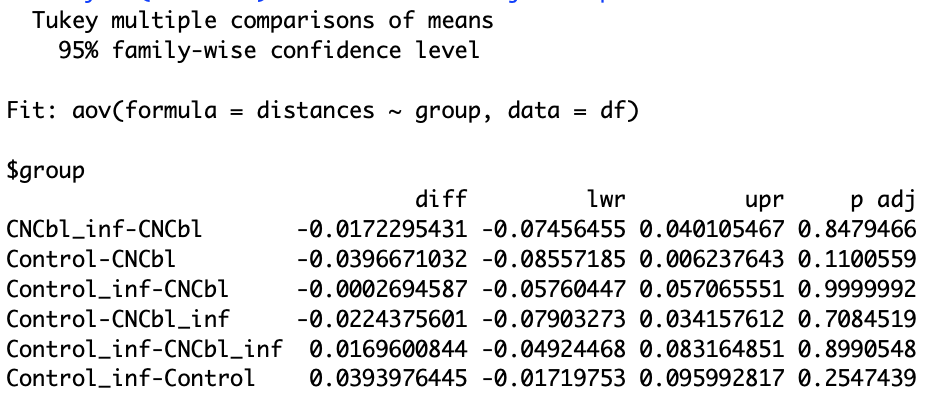
**

**Weighted UniFrac:**

colon2 <- ordinate(colon.rarefied, "PCoA", "unifrac", weighted=T)

colon2 <- plot_ordination(colon.rarefied, colon2, color = "Treatment", axes=c(1,2), shape= "Treatment") + scale_colour_manual(name = "Treatment",values = c("blue", "red", "dodgerblue2", "violetred3"), limits = c("Control","CNCbl","Control_inf","CNCbl_inf")) + scale_shape_manual(name = "Treatment",values = c(16,16,8,8), limits = c("Control","CNCbl","Control_inf","CNCbl_inf"))+ stat_ellipse(type = "t", linetype = 1, size = .5, level =.95) + ggtitle("Colon - Weighted UniFrac") + geom_point(size=4) + theme_bw() + theme(panel.grid.major = element_blank(), panel.grid.minor = element_blank()) + theme(plot.title = element_text(size=18, face= "bold", hjust=0.5)) + theme(text = element_text(size = 14, face= "bold")) + guides(fill = guide_legend(override.aes = list(linetype = 0)), color = guide_legend(override.aes = list(linetype = 0))) + theme(legend.title=element_blank())

colon2 + xlab("PC1 [34.4%]") + ylab("PC2 [27%]") + theme(legend.position =c(.85,.15))

colon2a = phyloseq::distance(colon.rarefied, "unifrac", weighted=T)

pairwise.adonis.dm(colon2a, phyloseq::sample_data(colon.rarefied)$Treatment)

**
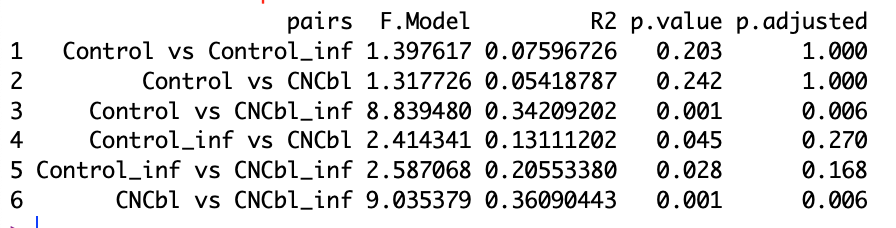
**

colon2aa <- betadisper(colon2a, phyloseq::sample_data(colon.rarefied)$Treatment)

permutest(colon2aa, pairwise=T , permutations = 999)

TukeyHSD(colon2aa)

**
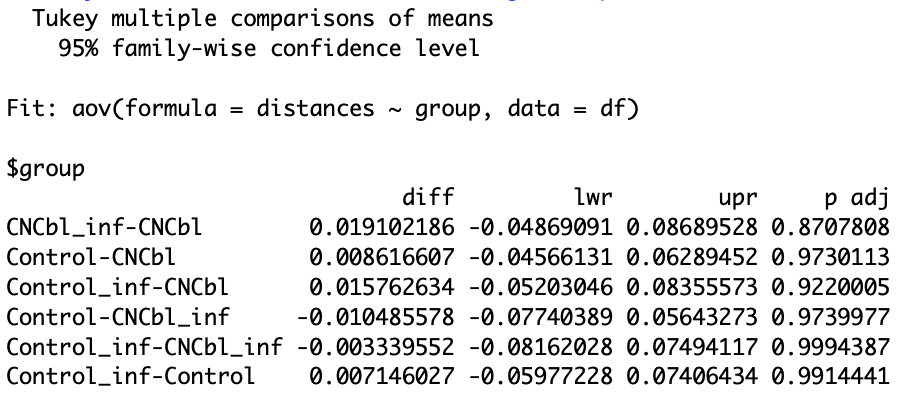
**

**Alpha Diversity:**

estimate_richness(colon)

colon.adiv <- data.frame( "Chao1" = phyloseq::estimate_richness(colon.rarefied, measures = "Chao1"), "Shannon" = phyloseq::estimate_richness(colon.rarefied, measures = "Shannon"), "Obversed" = phyloseq::estimate_richness(colon.rarefied, measures = "Observed"), "PD" = picante::pd(samp = data.frame(t(data.frame(phyloseq::otu_table(colon.rarefied)))), tree = phyloseq::phy_tree(colon.rarefied))[,1], "Treatment" = phyloseq::sample_data(colon.rarefied)$Treatment)

colon.adiv$Treatment = factor(sample_data(colon.rarefied)$Treatment, levels = c("Control","CNCbl","Control_inf","CNCbl_inf"))

levels(colon.adiv$Treatment)

colon.adiv %>%

gather(key = metric, value = value, c("Observed", "Shannon", "PD")) %>%

mutate(metric = factor(metric, levels = c("Observed", "Shannon", "PD"))) %>%

ggplot(aes(x = Treatment, y = value, colour = Treatment)) + geom_boxplot(outlier.color = NA) + scale_colour_manual(name = "Treatment",values = c("blue", "red", "dodgerblue2", "violetred3"), limits = c("Control","CNCbl","Control_inf","CNCbl_inf")) + scale_shape_manual(name = "Treatment", values = c(16,16,8,8), limits = c("Control","CNCbl","Control_inf","CNCbl_inf")) + geom_jitter(aes(color = Treatment, shape = Treatment), height = 0, width = .2, size = 3) + labs(x = "", y = "") + facet_wrap(~metric, scales = "free_y") + theme_bw() + theme(panel.grid.major = element_blank(), panel.grid.minor = element_blank()) + theme(strip.text.x = element_text(size=16, face="bold")) + theme(axis.text.x = element_blank(), axis.text.y = element_text(face="bold", size=12, color = "black")) + theme(legend.position="none") + scale_y_continuous(expand = expansion(mult = c(0.1,0.3)))

ANOVA = aov(colon.adiv$Observed ~ colon.adiv$Treatment)

TUKEY <- TukeyHSD(ANOVA, 'colon.adiv$Treatment', conf.level=0.95)

TUKEY


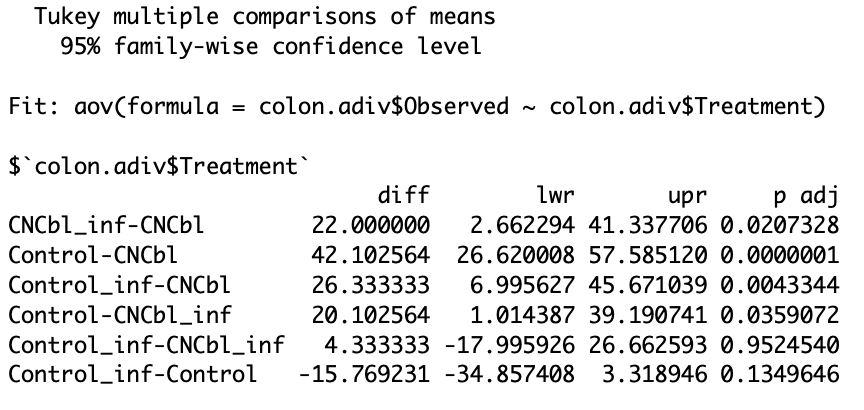


ANOVA = aov(colon.adiv$Shannon ~ colon.adiv$Treatment)

TUKEY <- TukeyHSD(ANOVA, 'colon.adiv$Treatment', conf.level=0.95)

TUKEY


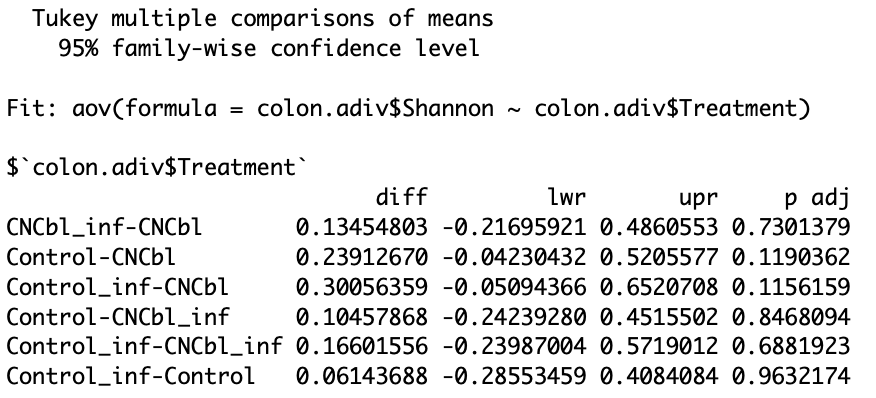


ANOVA = aov(colon.adiv$PD ~ colon.adiv$Treatment)

TUKEY <- TukeyHSD(ANOVA, 'colon.adiv$Treatment', conf.level=0.95)

TUKEY


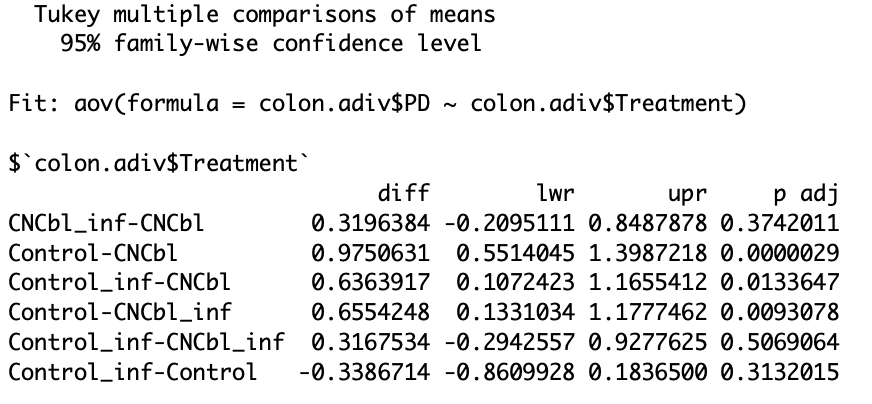


**Deseq2 Analysis: See ‘Microbiota Deseq2 Results Tables’ for results**

alpha = 0.05 ### significance cutoff

####### Naïve ########

colon.naïve <- subset_samples(colon.filtered, Infection=="N")

colon.naïve <- tree_glom(colon.naïve, 0.05)

colon.naïve

sample_data(colon.naïve)$Treatment <- factor(sample_data(colon.naïve)$Treatment, levels = c("Control","CNCbl"))

ds3a = phyloseq_to_deseq2(colon.naïve, ~Treatment)

ds3a = DESeq(ds3a)

res5 = results(ds3a, contrast=c("Treatment", "CNCbl", "Control"), alpha=alpha)

res5 = res5[order(res5$pvalue, na.last=NA), ]

res5

res5 = cbind(as(res5, "data.frame"), as(tax_table(colon.naïve)[rownames(res5), ], "matrix"))

res_sig5 = res5[(res5$pvalue < alpha), ]

res_sig5

df1 <- data.matrix(row.names(res_sig5))

colnames(df1) <- c("ASV")

fix(df1)

res_sig5 <- cbind(res_sig5, df1)

res_sig5$ID = paste(res_sig5$ASV, res_sig5$Phylum, res_sig5$Family, res_sig5$Genus, res_sig5$Species, sep="|")

fix(res_sig5)

ggplot(data = res_sig5, aes(y= reorder(ID, log2FoldChange), x=log2FoldChange, fill = log2FoldChange < 0)) + geom_bar(stat="identity") + scale_fill_manual(values = c("red", "blue")) + theme_bw() + theme(panel.grid.major = element_blank(), panel.grid.minor = element_blank()) + labs(y = "") + theme(legend.position="none") + theme(axis.text.x = element_text(face="bold", size=12, color = "black"), axis.text.y = element_text(size=14 , color = "black"), axis.title.x = element_text(size=12, color = "black"), plot.title = element_text(size = 18, face="bold")) + ggtitle("Colon - Control vs. CNCbl") + scale_x_continuous(limits = c(-10,10)) + scale_y_discrete(position = "right")

####### Infected #########

colon.infected <- subset_samples(colon.filtered, Infection=="Y")

colon.infected <- tree_glom(colon.infected, 0.05)

colon.infected

sample_data(colon.infected)$Treatment <- factor(sample_data(colon.infected)$Treatment, levels = c("Control_inf","CNCbl_inf"))

ds3b = phyloseq_to_deseq2(colon.infected, ~Treatment)

ds3b = DESeq(ds3b)

res6 = results(ds3b, contrast=c("Treatment", "CNCbl_inf", "Control_inf"), alpha=alpha)

res6 = res6[order(res6$pvalue, na.last=NA), ]

res6

res6 = cbind(as(res6, "data.frame"), as(tax_table(colon.infected)[rownames(res6), ], "matrix"))

res_sig6 = res6[(res6$pvalue < alpha), ]

res_sig6

df2 <- data.matrix(row.names(res_sig6))

colnames(df2) <- c("ASV")

fix(df2)

res_sig6 <- cbind(res_sig6, df2)

res_sig6$ID = paste(res_sig6$ASV, res_sig6$Phylum, res_sig6$Family, res_sig6$Genus, res_sig6$Species, sep="|")

fix(res_sig6)

ggplot(data = res_sig6, aes(y= reorder(ID, log2FoldChange), x=log2FoldChange, fill = log2FoldChange < 0)) + geom_bar(stat="identity") + scale_fill_manual(values = c("violetred3", "dodgerblue2")) + theme_bw() + theme(panel.grid.major = element_blank(), panel.grid.minor = element_blank()) + labs(y = "") + theme(legend.position="none") + theme(axis.text.x = element_text(face="bold", size=12, color = "black"), axis.text.y = element_text(size=14 , color = "black"), axis.title.x = element_text(size=12, color = "black"), plot.title = element_text(size = 18, face="bold")) + ggtitle("Colon - Control_inf vs. CNCbl_inf") + scale_y_discrete(position = "right") + scale_x_continuous(limits = c(-10,10))

**COLON NAÏVE (SURV vs EPC)**

**Opted for a non-rarefied analysis as to compare with rarefied reads per literature {Formatting Citation}, minimal differences can be noted in final results (Fig 3a colon Vs Fig.4e)**

*****NOTE:**

**ABP+ (or ABPp) refers to the EPC experiment**

**ABP- (or ABPn) refers to the SURV experiment**

colon.allexp <- subset_samples(physeq.clean, Section=="Colon")

colon.allnaïve <- subset_samples(colon.allexp, Infection=="N")

colon.allnaïve.filtered <- filter_taxa(colon.allnaïve.filtered, function(x) sum(x > 3) > (0.2*length(x)), TRUE )

sample_data(colon.allnaïve.filtered)$Group = paste(sample_data(colon.allnaïve.filtered)$Treatment, sample_data(colon.allnaïve.filtered)$Microbiome, sep=" ")

**Beta Diverstiy:**

**Unweighted UniFrac:**

distance1 <- ordinate(colon.allnaïve.filtered, "PCoA", "unifrac", weighted=F)

ordplot1 <- plot_ordination(colon.allnaïve.filtered, distance1, color = "Group", axes=c(1,2), shape= "Group") + scale_colour_manual(name = "Group",values = c("blue", "red", "blue", "red"), limits = c("Control ABP-","CNCbl ABP-","Control ABP+","CNCbl ABP+")) + scale_shape_manual(name = "Group",values = c(0,0,15,15), limits = c("Control ABP-","CNCbl ABP-","Control ABP+","CNCbl ABP+")) + stat_ellipse(type = "t", linetype = 1, size = .4, level =.95) + ggtitle("Colon - Unweighted UniFrac") + theme(text = element_text(size = 14)) + geom_point(size=4) + theme_bw() + theme(panel.grid.major = element_blank(), panel.grid.minor = element_blank()) + theme(plot.title = element_text(size=18, face= "bold", hjust=0.5)) + theme(text = element_text(size = 14, face= "bold")) + guides(fill = guide_legend(override.aes = list(linetype = 0)), color = guide_legend(override.aes = list(linetype = 0))) + theme(legend.title=element_blank(), panel.border = element_rect(colour="black", fill=NA))

ordplot1 + xlab("PC1 [61.8%]") + ylab("PC2 [15%]") + theme(legend.position =c(.85,.15))

physeq_unweighted = phyloseq::distance(colon.allnaïve.filtered, "unifrac", weighted=F)

pairwise.adonis.dm(physeq_unweighted, phyloseq::sample_data(colon.allnaïve.filtered)$Group)

**
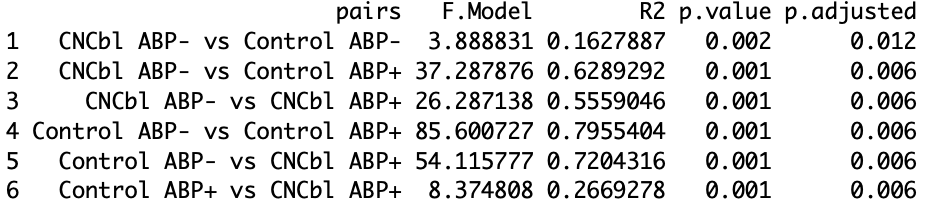
**

p1 <- betadisper(physeq_unweighted, phyloseq::sample_data(colon.allnaïve.filtered)$Group)

permutest(p1, pairwise=T , permutations = 999)

TukeyHSD(p1)


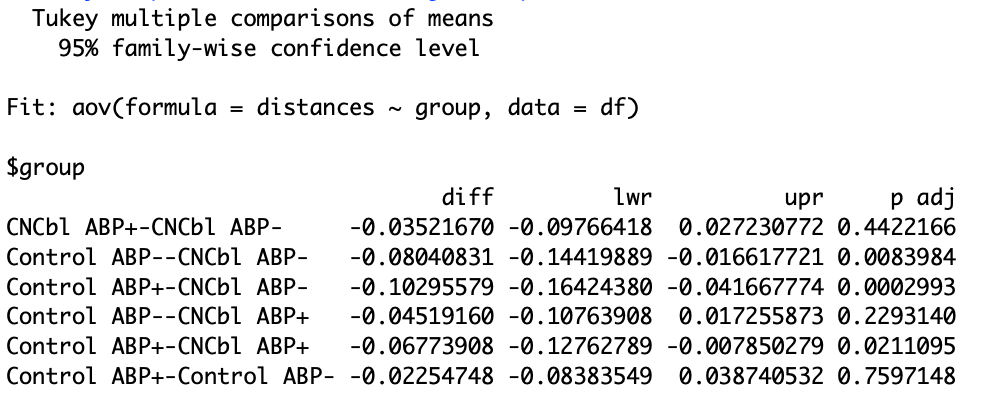


**Weighted UniFrac:**

distance2 <- ordinate(colon.allnaïve.filtered, "PCoA", "unifrac", weighted=T)

ordplot2 <- plot_ordination(colon.allnaïve.filtered, distance2, color = "Group", axes=c(1,2), shape= "Group") + geom_point(size=4) + scale_colour_manual(name = "Group", values = c("blue", "red", "blue", "red"), limits = c("Control ABP-","CNCbl ABP-","Control ABP+","CNCbl ABP+")) + scale_shape_manual(name = "Group", values = c(0,0,15,15), limits = c("Control ABP-","CNCbl ABP-","Control ABP+","CNCbl ABP+")) + stat_ellipse(type = "t", linetype = 1, size = .4, level =.95) + ggtitle("Colon - Weighted UniFrac") + geom_point(size=4) + theme(text = element_text(size = 14)) + theme_bw() + theme(panel.grid.major = element_blank(), panel.grid.minor = element_blank()) + theme(plot.title = element_text(size=18, face= "bold", hjust=0.5)) + theme(text = element_text(size = 14, face= "bold")) + guides(fill = guide_legend(override.aes = list(linetype = 0)), color = guide_legend(override.aes = list(linetype = 0))) + theme(legend.title=element_blank())

ordplot2 + xlab("PC1 [69.7%]") + ylab("PC2 [13.6%]") + theme(legend.position =c(.85,.85))

physeq_weighted = phyloseq::distance(colon.allnaïve.filtered, "unifrac", weighted=T)

pairwise.adonis.dm(physeq_weighted, phyloseq::sample_data(colon.allnaïve.filtered)$Group)

**
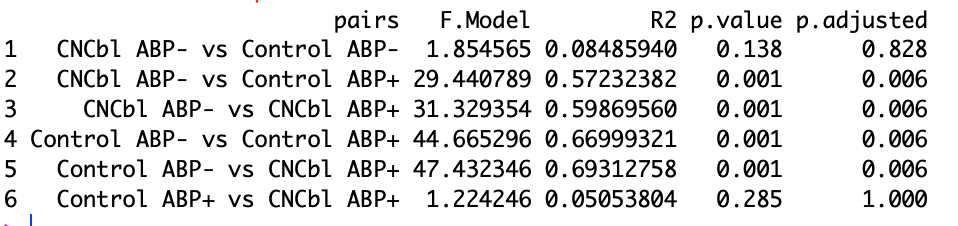
**

p2 <- betadisper(physeq_weighted, phyloseq::sample_data(colon.allnaïve.filtered)$Group)

permutest(p2, pairwise= T, permutation= 999)

TukeyHSD(p2)

**
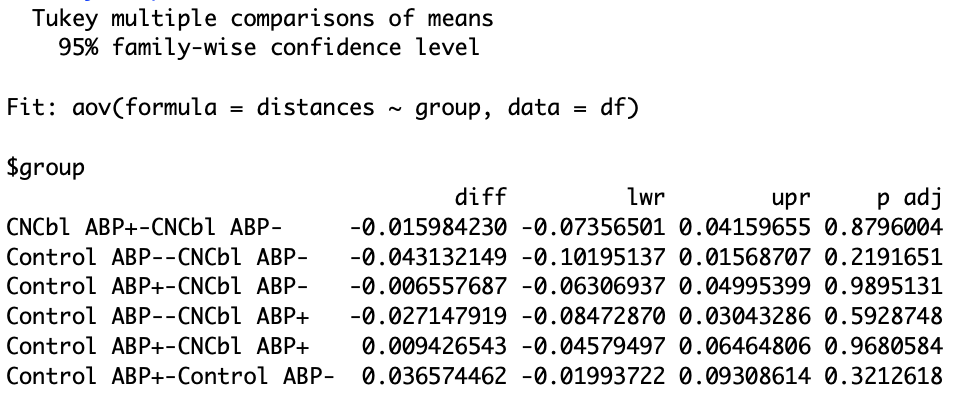
**

**Alpha Diversity:**

estimate_richness(colon.allnaïve.filtered)

adiv <- data.frame( "Chao1" = phyloseq::estimate_richness(colon.allnaïve.filtered, measures = "Chao1"), "Shannon" = phyloseq::estimate_richness(colon.allnaïve.filtered, measures = "Shannon"), "Obversed" = phyloseq::estimate_richness(colon.allnaïve.filtered, measures = "Observed"), "PD" = picante::pd(samp = data.frame(t(data.frame(phyloseq::otu_table(colon.allnaïve.filtered)))), tree = phyloseq::phy_tree(colon.allnaïve.filtered))[,1], "Group" = phyloseq::sample_data(colon.allnaïve.filtered)$Treatment)

adiv$Group = factor(sample_data(colon.allnaïve.filtered)$Group, levels = c("Control ABP-","CNCbl ABP-","Control ABP+","CNCbl ABP+"))

levels(adiv$Group)

adiv %>%

gather(key = metric, value = value, c("Observed", "Shannon", "PD")) %>%

mutate(metric = factor(metric, levels = c("Observed", "Shannon", "PD"))) %>%

ggplot(aes(x = Group, y = value, colour = Group)) + geom_boxplot(outlier.color = NA) + scale_colour_manual(name = "Group",values = c("blue", "red", "blue", "red"), limits = c("Control ABP-","CNCbl ABP-","Control ABP+","CNCbl ABP+")) + scale_shape_manual(name = "Group",values = c(0,0,15,15), limits = c("Control ABP-","CNCbl ABP-","Control ABP+","CNCbl ABP+")) + geom_jitter(aes(color = Group, shape = Group), height = 0, width = .2, size = 3) + labs(x = "", y = "") + facet_wrap(~metric, scales = "free_y") + theme_bw() + theme(panel.grid.major = element_blank(), panel.grid.minor = element_blank()) + theme(strip.text.x = element_text(size=16, face="bold")) + theme(axis.text.x = element_blank(), axis.text.y = element_text(face="bold", size=12, color = "black")) + theme(legend.position="none") + scale_y_continuous(expand = expansion(mult = c(0.1,0.3)))

ANOVA = aov(adiv$Observed ~ adiv$Group)

TUKEY <- TukeyHSD(ANOVA, 'adiv$Group', conf.level=0.95)

TUKEY

**
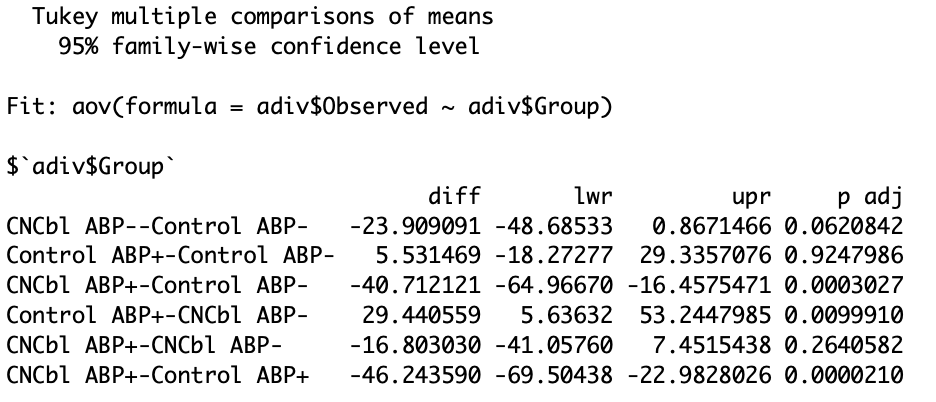
**

ANOVA = aov(adiv$Shannon ~ adiv$Group)

TUKEY <- TukeyHSD(ANOVA, 'adiv$Group', conf.level=0.95)

TUKEY

**
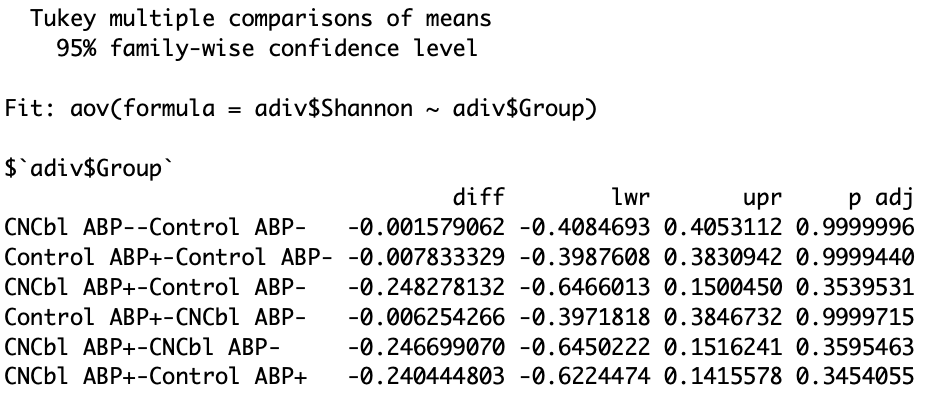
**

ANOVA = aov(adiv$PD ~ adiv$Group)

TUKEY <- TukeyHSD(ANOVA, 'adiv$Group', conf.level=0.95)

TUKEY

**
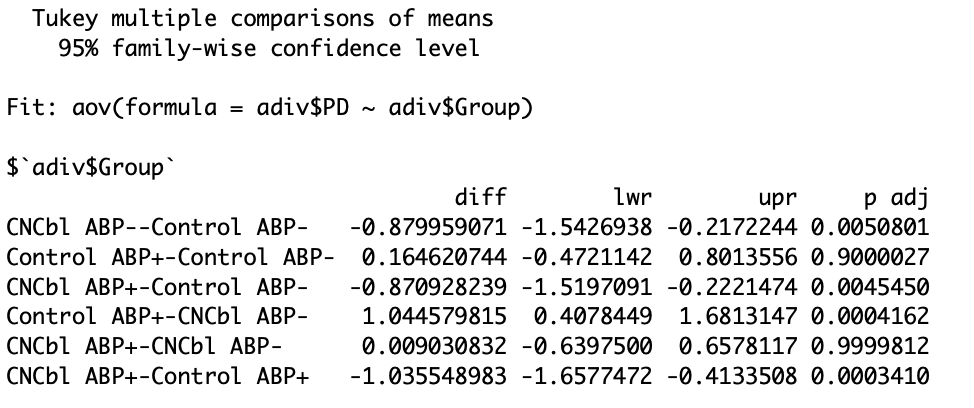
**

**Deseq2 Analysis: See ‘Microbiota Deseq2 Results Tables’ for results**

alpha = 0.05 ### significance cutoff

####### ABPn SURV experiment ########

ABPn <- subset_samples(colon.allnaïve.filtered, Microbiome2=="ABPn")

ABPn.glom <- tree_glom(ABPn, 0.05)

ABPn.glom

sample_data(ABPn.glom)$Group2 <- factor(sample_data(ABPn.glom)$Group2, levels = c("Control_ABPn","CNCbl_ABPn"))

ds4a = phyloseq_to_deseq2(ABPn.glom, ~Group2)

ds4a = DESeq(ds4a)

res7 = results(ds4a, contrast=c("Group2", "CNCbl_ABPn", "Control_ABPn"), alpha=alpha)

res7 = res7[order(res7$pvalue, na.last=NA), ]

res7 = cbind(as(res7, "data.frame"), as(tax_table(ABPn.glom)[rownames(res7), ], "matrix"))

res_sig7 = res7[(res7$pvalue < alpha), ]

df1 <- data.matrix(row.names(res_sig7))

colnames(df1) <- c("ASV")

fix(df1)

res_sig7 <- cbind(res_sig7, df1)

res_sig7$ID = paste(res_sig7$ASV, res_sig7$Phylum, res_sig7$Family, res_sig7$Genus, res_sig7$Species, sep="|")

fix(res_sig7)

ggplot(data = res_sig7, aes(y= reorder(ID, log2FoldChange), x=log2FoldChange, fill = log2FoldChange < 0)) + geom_bar(stat="identity") + scale_fill_manual(values = c("red", "blue")) + theme_bw() + theme(panel.grid.major = element_blank(), panel.grid.minor = element_blank()) + labs(y = "") + theme(legend.position="none") + theme(axis.text.x = element_text(face="bold", size=12, color = "black"), axis.text.y = element_text(size=14 , color = "black"), axis.title.x = element_text(size=12, color = "black"), plot.title = element_text(size = 18, face="bold")) + ggtitle("Colon - Control vs. CNCbl [ABP-]") + scale_x_continuous(limits = c(-10,10)) + scale_y_discrete(position = "right")

####### ABPp (EPC experiment) ########

ABPp <- subset_samples(colon.allnaïve.filtered, Microbiome2=="ABPp")

ABPp.glom <- tree_glom(ABPp, 0.05)

ABPp.glom

sample_data(ABPp.glom)$Group2 <- factor(sample_data(ABPp.glom)$Group2, levels = c("Control_ABPp","CNCbl_ABPp"))

ds4b = phyloseq_to_deseq2(ABPp.glom, ~Group2)

ds4b = DESeq(ds4b)

res8 = results(ds4b, contrast=c("Group2", "CNCbl_ABPp", "Control_ABPp"), alpha=alpha)

res8 = res8[order(res8$pvalue, na.last=NA), ]

res8 = cbind(as(res8, "data.frame"), as(tax_table(ABPp.glom)[rownames(res8), ], "matrix"))

res_sig8 = res8[(res8$pvalue < alpha), ]

df1 <- data.matrix(row.names(res_sig8))

colnames(df1) <- c("ASV")

fix(df1)

res_sig8 <- cbind(res_sig8, df1)

res_sig8$ID = paste(res_sig8$ASV, res_sig8$Phylum, res_sig8$Family, res_sig8$Genus, res_sig8$Species, sep="|")

ggplot(data = res_sig8, aes(y= reorder(ID, log2FoldChange), x=log2FoldChange, fill = log2FoldChange < 0)) + geom_bar(stat="identity") + scale_fill_manual(values = c("red", "blue")) + theme_bw() + theme(panel.grid.major = element_blank(), panel.grid.minor = element_blank()) + labs(y = "") + theme(legend.position="none") + theme(axis.text.x = element_text(face="bold", size=12, color = "black"), axis.text.y = element_text(size=14 , color = "black"), axis.title.x = element_text(size=12, color = "black"), plot.title = element_text(size = 18, face="bold")) + ggtitle("Colon - Control vs. CNCbl [ABP+]") + scale_x_continuous(limits = c(-10,10)) + scale_y_discrete(position = "right")

####### Control ABPn VS Control ABPp #########

data <- sample_data(ABPpABPn)

fix(data)

ABPpABPn <- subset_samples(colon.allnaïve.filtered, Treatment=="Control")

ABPpABPn.glom <- tree_glom(ABPpABPn, 0.1)

ABPpABPn.glom

sample_data(ABPpABPn.glom)$Group2 <- factor(sample_data(ABPpABPn.glom)$Group2, levels = c("Control_ABPp", "Control_ABPn"))

ds4c = phyloseq_to_deseq2(ABPpABPn.glom, ~Group2)

ds4c = DESeq(ds4c)

res9 = results(ds4c, contrast=c("Group2", "Control_ABPp", "Control_ABPn"), alpha=alpha)

res9 = res9[order(res9$pvalue, na.last=NA), ]

res9

res9 = cbind(as(res9, "data.frame"), as(tax_table(ABPpABPn.glom)[rownames(res9), ], "matrix"))

res_sig9 = res9[(res9$pvalue < alpha), ]

res_sig9

df1 <- data.matrix(row.names(res_sig9))

colnames(df1) <- c("ASV")

fix(df1)

res_sig9 <- cbind(res_sig9, df1)

res_sig9$ID = paste(res_sig9$ASV, res_sig9$Phylum, res_sig9$Family, res_sig9$Genus, res_sig9$Species, sep="|")

fix(res_sig9)

ggplot(data = res_sig9, aes(y= reorder(ID, log2FoldChange), x=log2FoldChange, fill = log2FoldChange < 0)) + geom_bar(stat="identity") + scale_fill_manual(values = c("blue", "blue")) + theme_bw() + theme(panel.grid.major = element_blank(), panel.grid.minor = element_blank()) + labs(y = "") + theme(legend.position="none") + theme(axis.text.x = element_text(face="bold", size=12, color = "black"), axis.text.y = element_text(size=14 , color = "black"), axis.title.x = element_text(size=12, color = "black"), plot.title = element_text(size = 18, face="bold")) + ggtitle("Colon - Control [ABP-] vs. Control [ABP+]") + scale_x_continuous(limits = c(-20,20)) + scale_y_discrete(position = "right")

**RNAseq cecal microbiota (SAMSA2 filtered ribosomal data)**

**Qiime2-Deblur**

physeq.rm <- filter_taxa(physeq, function(x){sum(x > 0) > 1}, prune = T)

physeq.clean <- subset_taxa(physeq.rm, (Genus!="Chloroplast") | is.na(Genus))

physeq.clean <- subset_taxa(physeq.clean, (Genus!="Mitochondria") | is.na(Genus))

physeq.clean <- subset_taxa(physeq.clean, (Phylum!="NA"))

physeq.filtered <- filter_taxa(physeq.clean, function(x) sum(x > 3) > (0.2*length(x)), TRUE )

min(sample_sums(physeq.filtered)) ###1247061

physeq.rarefied <- rarefy_even_depth(physeq.filtered, sample.size = min(sample_sums(physeq.filtered)), rngseed=1)

**Beta Diversity**

**Unweighted UniFrac:**

p1 <- ordinate(physeq.rarefied, "PCoA", "unifrac", weighted=F)

p1 <- plot_ordination(physeq.rarefied, p1, color = "Treatment", axes=c(1,2), shape= "Treatment") + scale_colour_manual(name = "Treatment",values = c("blue", "red", "dodgerblue2", "violetred3"), limits = c("Control","CNCbl","Control_inf","CNCbl_inf")) + scale_shape_manual(name = "Treatment",values = c(16,16,8,8), limits = c("Control","CNCbl","Control_inf","CNCbl_inf"))+ stat_ellipse(type = "t", linetype = 1, size = .5, level =.95) + ggtitle("RNAseq - Unweighted UniFrac") + geom_point(size=4) + theme_bw() + theme(panel.grid.major = element_blank(), panel.grid.minor = element_blank()) + theme(plot.title = element_text(size=18, face= "bold", hjust=0.5)) + theme(text = element_text(size = 14, face= "bold")) + guides(fill = guide_legend(override.aes = list(linetype = 0)), color = guide_legend(override.aes = list(linetype = 0))) + theme(legend.title=element_blank())

p1 + xlab("PC1 [21.4%]") + ylab("PC2 [14%]") + theme(legend.position =c(.87,.85))

p1a = phyloseq::distance(physeq.rarefied, "unifrac", weight=F)

pairwise.adonis.dm(p1a, phyloseq::sample_data(physeq.rarefied)$Treatment)


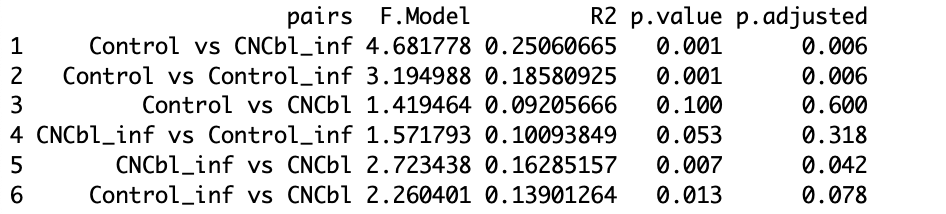


p1aa <- betadisper(p1a, phyloseq::sample_data(physeq.rarefied)$Treatment)

permutest(p1aa, pairwise=T , permutations = 999)

TukeyHSD(p1aa)

**
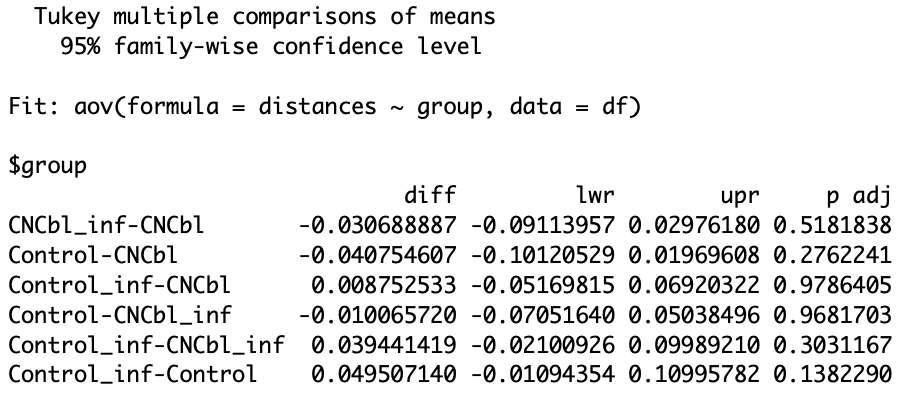
**

**Weighted UniFrac:**

p2 <- ordinate(physeq.rarefied, "PCoA", "unifrac", weighted=T)

p2 <- plot_ordination(physeq.rarefied, p2, color = "Treatment", axes=c(1,2), shape= "Treatment") + scale_colour_manual(name = "Treatment",values = c("blue", "red", "dodgerblue2", "violetred3"), limits = c("Control","CNCbl","Control_inf","CNCbl_inf")) + scale_shape_manual(name = "Treatment",values = c(16,16,8,8), limits = c("Control","CNCbl","Control_inf","CNCbl_inf"))+ stat_ellipse(type = "t", linetype = 1, size = .5, level =.95) + ggtitle("Cecum - Weighted UniFrac") + geom_point(size=4) + theme_bw() + theme(panel.grid.major = element_blank(), panel.grid.minor = element_blank()) + theme(plot.title = element_text(size=18, face= "bold", hjust=0.5)) + theme(text = element_text(size = 14, face= "bold")) + guides(fill = guide_legend(override.aes = list(linetype = 0)), color = guide_legend(override.aes = list(linetype = 0))) + theme(legend.title=element_blank())

p2 + xlab("PC1 [37.1%]") + ylab("PC2 [19.6%]") + theme(legend.position =c(.87,.85))

p2a = phyloseq::distance(physeq.rarefied, "unifrac", weighted=T)

pairwise.adonis.dm(p2a, phyloseq::sample_data(physeq.rarefied)$Treatment)


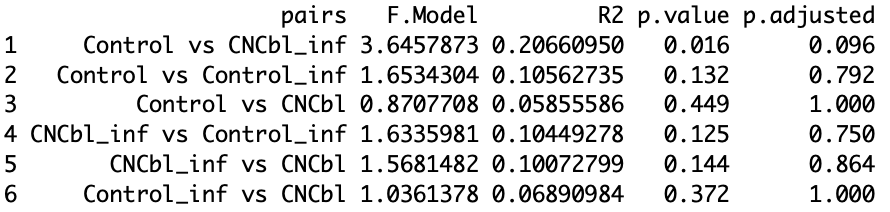


p2aa <- betadisper(p2a, phyloseq::sample_data(physeq.rarefied)$Treatment)

permutest(p2aa, pairwise=T , permutations = 999)

TukeyHSD(p2aa)

**
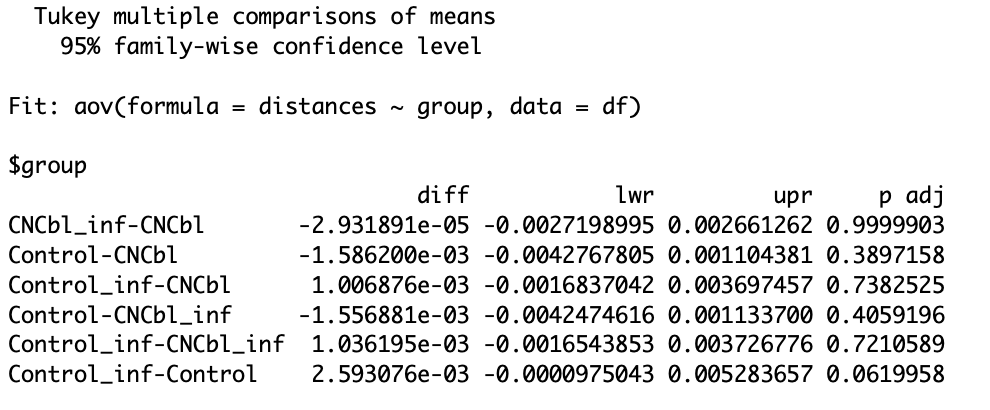
**

**Alpha Diversity:**

estimate_richness(physeq.rarefied)

adiv <- data.frame( "Chao1" = phyloseq::estimate_richness(physeq.rarefied, measures = "Chao1"), "Shannon" = phyloseq::estimate_richness(physeq.rarefied, measures = "Shannon"), "Obversed" = phyloseq::estimate_richness(physeq.rarefied, measures = "Observed"), "PD" = picante::pd(samp = data.frame(t(data.frame(phyloseq::otu_table(physeq.rarefied)))), tree = phyloseq::phy_tree(physeq.rarefied))[,1], "Treatment" = phyloseq::sample_data(physeq.rarefied)$Treatment)

adiv$Treatment = factor(sample_data(physeq.rarefied)$Treatment, levels = c("Control","CNCbl","Control_inf","CNCbl_inf"))

levels(adiv$Treatment)

adiv %>%

gather(key = metric, value = value, c("Observed", "Shannon", "PD")) %>%

mutate(metric = factor(metric, levels = c("Observed", "Shannon", "PD"))) %>%

ggplot(aes(x = Treatment, y = value, colour = Treatment)) + geom_boxplot(outlier.color = NA) + scale_colour_manual(name = "Treatment",values = c("blue", "red", "dodgerblue2", "violetred3"), limits = c("Control","CNCbl","Control_inf","CNCbl_inf")) + scale_shape_manual(name = "Treatment", values = c(16,16,8,8), limits = c("Control","CNCbl","Control_inf","CNCbl_inf")) + geom_jitter(aes(color = Treatment, shape = Treatment), height = 0, width = .2, size = 3) + labs(x = "", y = "") + facet_wrap(~metric, scales = "free_y") + theme_bw() + theme(panel.grid.major = element_blank(), panel.grid.minor = element_blank()) + theme(strip.text.x = element_text(size=16, face="bold")) + theme(axis.text.x = element_blank(), axis.text.y = element_text(face="bold", size=12, color = "black")) + theme(legend.position="none") + scale_y_continuous(expand = expansion(mult = c(0.1,0.3)))

ANOVA = aov(adiv$Observed ~ adiv$Treatment)

TUKEY <- TukeyHSD(ANOVA, 'adiv$Treatment', conf.level=0.95)

TUKEY

**
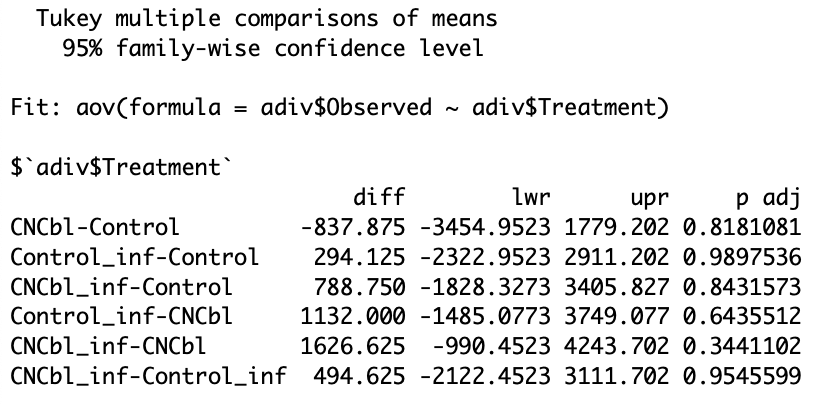
**

ANOVA = aov(adiv$Shannon ~ adiv$Treatment)

TUKEY <- TukeyHSD(ANOVA, 'adiv$Treatment', conf.level=0.95)

TUKEY

**
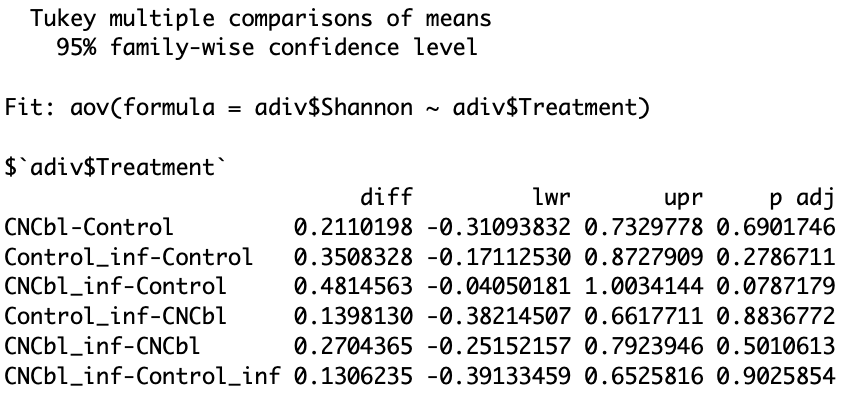
**

ANOVA = aov(adiv$PD ~ adiv$Treatment)

TUKEY <- TukeyHSD(ANOVA, 'adiv$Treatment', conf.level=0.95)

head(TUKEY)

TUKEY

**
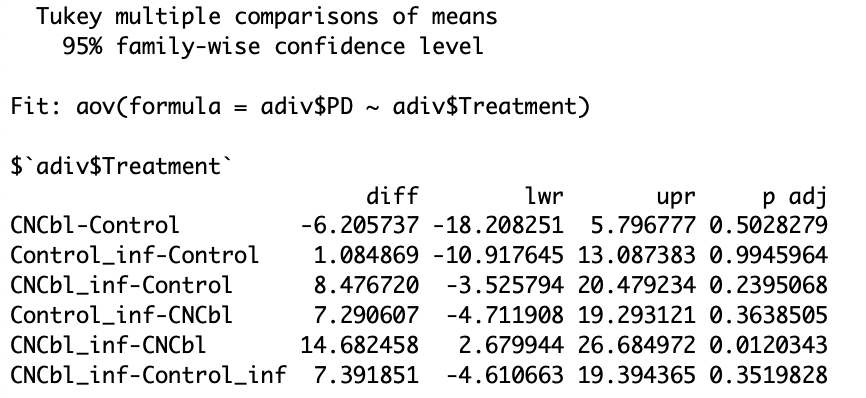
**

**Deseq2 Analysis: See ‘Microbiota Deseq2 Results Tables’ for results**

alpha = 0.05

####### Naïve ########

Naïve <- subset_samples(physeq.filtered, Infection=="N")

Naïve.glom <- tax_glom(Naïve, taxrank="Genus", NArm=F)

sample_data(Naïve.glom)$Treatment <- factor(sample_data(Naïve.glom)$Treatment, levels = c("Control","CNCbl"))

ds1a = phyloseq_to_deseq2(Naïve.glom, ~Treatment)

ds1a = DESeq(ds1a)

res1 = results(ds1a, contrast=c("Treatment", "CNCbl", "Control"), alpha=alpha)

res1 = res1[order(res1$pvalue, na.last=NA), ]

fix(res1)

res1 = cbind(as(res1, "data.frame"), as(tax_table(Naïve.glom)[rownames(res1), ], "matrix"))

res_sig1 = res1[(res1$pvalue < alpha), ]

res_sig1

df1 <- data.matrix(row.names(res_sig1))

colnames(df1) <- c("Barcode")

fix(df1)

res_sig1 <- cbind(res_sig1, df1)

res_sig1$ID = paste(res_sig1$Barcode, res_sig1$Phylum, res_sig1$Family, res_sig1$Genus, res_sig1$Species, sep="|")

fix(res_sig1)

ggplot(data = res_sig1, aes(y= reorder(ID, log2FoldChange), x=log2FoldChange, fill = log2FoldChange < 0)) + geom_bar(stat="identity") + scale_fill_manual(values = c("red","blue")) + theme_bw() + theme(panel.grid.major = element_blank(), panel.grid.minor = element_blank()) + labs(y = "") + theme(legend.position="none") + theme(axis.text.x = element_text(face="bold", size=12, color = "black"), axis.text.y = element_text(size=14, color = "black"), axis.title.x = element_text(size=12, color = "black"), plot.title = element_text(size = 18, face="bold")) + ggtitle("Control vs. CNCbl") + scale_x_continuous(limits = c(-10,10)) + scale_y_discrete(position = "right")

####### Infected #########

Infected <- subset_samples(physeq.filtered, Infection=="Y")

Infected.glom <- tax_glom(Infected, taxrank="Genus", NArm=F)

sample_data(Infected.glom)$Treatment <- factor(sample_data(Infected.glom)$Treatment, levels = c("Control_inf","CNCbl_inf"))

ds1b = phyloseq_to_deseq2(Infected.glom, ~Treatment)

ds1b = DESeq(ds1b)

res2 = results(ds1b, contrast=c("Treatment", "CNCbl_inf", "Control_inf"), alpha=alpha)

res2 = res2[order(res2$pvalue, na.last=NA), ]

fix(res2)

res2 = cbind(as(res2, "data.frame"), as(tax_table(Infected.glom)[rownames(res2), ], "matrix"))

res_sig2 = res2[(res2$pvalue < alpha), ]

df2 <- data.matrix(row.names(res_sig2))

colnames(df2) <- c("Barcode")

fix(df2)

res_sig2 <- cbind(res_sig2, df2)

res_sig2$ID = paste(res_sig2$Barcode, res_sig2$Phylum, res_sig2$Family, res_sig2$Genus, res_sig2$Species, sep="|")

fix(res_sig2)

ggplot(data = res_sig2, aes(y= reorder(ID, log2FoldChange), x=log2FoldChange, fill = log2FoldChange < 0)) + geom_bar(stat="identity") + scale_fill_manual(values = c("violetred3", "dodgerblue2")) + theme_bw() + theme(panel.grid.major = element_blank(), panel.grid.minor = element_blank()) + labs(y = "") + theme(legend.position="none") + theme(axis.text.x = element_text(face="bold", size=12, color = "black"), axis.text.y = element_text(size=14, color = "black"), axis.title.x = element_text(size=12, color = "black"), plot.title = element_text(size = 18, face="bold")) + ggtitle("Control_inf vs. CNCbl_inf") + scale_x_continuous(limits = c(-10,10)) + scale_y_discrete(position = "right")

**Correlation between colon microbiota normalized reads from Deseq2 and measured colon parameters including *C. rodentium* plating counts and inflammatory markers in infected and naïve mice.**

cor.matrix <- cor(x,y, method = "spearman")

pvalue <- corr.test(x,y, method="spearman", adjust="bonferroni", alpha=0.05)
